# Effectiveness, safety, initial optimal dose, and optimal maintenance dose range of basal insulin regimens for type 2 diabetes: A systematic review with meta‐analysis

**DOI:** 10.1111/1753-0407.13381

**Published:** 2023-04-10

**Authors:** Yingying Luo, Jun Xia, Zhan Zhao, Yaping Chang, Yong Mong Bee, Khue Thy Nguyen, Soo Lim, Daisuke Yabe, Margaret McGill, Alice Pik Shan Kong, Siew Pheng Chan, Marisa Deodat, Chaicharn Deerochanawong, Ketut Suastika, Chenchen Xu, Liming Chen, Wei Chen, Xiaoying Li, Weigang Zhao, Xiaomei Yao, Linong Ji

**Affiliations:** ^1^ Department of Endocrinology and Metabolism Peking University People's Hospital Beijing 100044 China; ^2^ Nottingham Ningbo GRADE Centre University of Nottingham Ningbo China Ningbo Zhejiang 315100 China; ^3^ Academic Unit of Lifespan and Population Health, School of Medicine The University of Nottingham Nottingham NG7 2UH UK; ^4^ Tianjin Tiantian Biotechnology Co., Ltd. Tianjin 300000 China; ^5^ OrthoEvidence Inc. Burlington Ontario L7N 3H8 Canada; ^6^ Department of Endocrinology Singapore General Hospital 169608 Singapore Singapore; ^7^ Ho Chi Minh University of Medicine and Pharmacy Medic Medical Center Ho Chi Minh City 700000 Vietnam; ^8^ Department of Internal Medicine Seoul National University College of Medicine and Seoul National University Bundang Hospital Seongnam 13620 South Korea; ^9^ Departments of Diabetes, Endocrinology and Metabolism/Rheumatology and Clinical Immunology Gifu University Graduate School of Medicine Gifu 501‐1194 Japan; ^10^ Center for One Medicine Innovative Translational Research Gifu University Institute for Advanced Study Gifu 501‐1194 Japan; ^11^ Diabetes Centre, Royal Prince Alfred Hospital, Faculty of Medicine and Health University of Sydney Sydney New South Wales 2050 Australia; ^12^ Division of Endocrinology, Department of Medicine and Therapeutics The Chinese University of Hong Kong, Hong Kong Special Administrative Region Hong Kong 999077 China; ^13^ Subang Jaya Medical Centre, Department of Medicine University of Malaya Medical Centre Lembah Pantai Kuala Lumpur 59100 Malaysia; ^14^ Michael G. DeGroote Cochrane Canada and McMaster GRADE Centres McMaster University Hamilton Ontario L8V 5C2 Canada; ^15^ College of Medicine, Rangsit University Bangkok 10400 Thailand; ^16^ Division of Endocrinology and Metabolism, Department of Internal Medicine, Faculty of Medicine Udayana University, Prof. IGNG Ngoerah Hospital Denpasar Bali 80114 Indonesia; ^17^ Chu Hsien‐I Memorial (Metabolic Diseases) Hospital & Tianjin Institute of Endocrinology, Tianjin Medical University Tianjin 300134 China; ^18^ Department of Clinical Nutrition, Department of Health Medicine Chinese Academy of Medical Sciences‐Peking Union Medical College, Peking Union Medical College Hospital Beijing 100730 China; ^19^ Department of Endocrinology and Metabolism, Zhongshan Hospital Fudan University Shanghai 200032 China; ^20^ Department of Endocrinology Peking Union Medical College Hospital Beijing 100730 China; ^21^ Center for Clinical Practice Guideline Conduction and Evaluation Children's Hospital of Fudan University Shanghai 201100 China; ^22^ Department of Health Research Methods, Evidence, and Impact McMaster University Hamilton Ontario L8V 5C2 Canada

**Keywords:** basal insulin, effectiveness, initiation and maintenance dose, safety, systematic review, type 2 diabetes, 基础胰岛素, 有效性, 起始和维持剂量, 安全, 系统评价, 2型糖尿病

## Abstract

**Aims:**

To investigate the effectiveness, safety, optimal starting dose, optimal maintenance dose range, and target fasting plasma glucose of five basal insulins in insulin‐naïve patients with type 2 diabetes mellitus.

**Methods:**

MEDLINE, EMBASE, Web of Science, and the Cochrane Library were searched from January 2000 to February 2022. The Preferred Reporting Items for Systematic Reviews and Meta‐Analyses (PRISMA) guidelines were followed and the Grading of Recommendations, Assessment, Development, and Evaluations (GRADE) approach was adopted. The registration ID is CRD42022319078 in PROSPERO.

**Results:**

Among 11 163 citations retrieved, 35 publications met the planned criteria. From meta‐analyses and network meta‐analyses, we found that when injecting basal insulin regimens at bedtime, the optimal choice in order of most to least effective might be glargine U‐300 or degludec U‐100, glargine U‐100 or detemir, followed by neutral protamine hagedorn (NPH). Injecting glargine U‐100 in the morning may be more effective (ie, more patients archiving glycated hemoglobin < 7.0%) and lead to fewer hypoglycemic events than injecting it at bedtime. The optimal starting dose for the initiation of any basal insulins can be 0.10–0.20 U/kg/day. There is no eligible evidence to investigate the optimal maintenance dose for basal insulins.

**Conclusions:**

The five basal insulins are effective for the target population. Glargine U‐300, degludec U‐100, glargine U‐100, and detemir lead to fewer hypoglycemic events than NPH without compromising glycemic control.

## Graphical abstract

Highlights

This is a systematic review and meta‐analysis. MEDLINE, EMBASE, Web of Science, and the Cochrane Library were searched from January 2000 to February 2022. Among 11,163 citations retrieved, 35 publications met the pre‐planned criteria. The five basal insulins are effective for insulin‐naive adult patients with T2DM. Glargine U‐300, degludec U‐100, glargine U‐100, and detemir lead to fewer hypoglycemic events than NPH without compromising glycemic control.

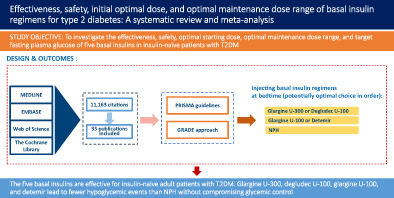

## 摘要

**目的:**探讨5种基础胰岛素治疗在尚未接受胰岛素治疗的2型糖尿病(T2DM)患者的有效性、安全性、最佳起始剂量、最佳维持剂量范围及空腹血糖目标。

**方法:**检索2000年1月至2022年2月MEDLINE、EMBASE、Web of Science、Cochrane Library等数据库。遵循系统综述和*meta*分析的首选报告项目(PRISMA)指南并采用建议、评估、发展和评价的分级(GRADE)方法。在PROSPERO的注册ID为CRD42022319078。

**结果:**共检索到文献11163篇, 符合纳入标准的文献35篇。从*meta*分析和网络*meta*分析中, 我们发现, 在睡前注射基础胰岛素方案时, 最优选择可能是甘精胰岛素U‐300或德谷胰岛素U‐100、甘精胰岛素U‐100或地特胰岛素, 其次是低精蛋白锌胰岛素(NPH)。与睡前注射相比, 早晨注射甘精胰岛素U‐100可能更有效(即更多患者能够实现HbA_1c_<7.0%), 且低血糖事件更少。任何基础胰岛素起始的最佳起始剂量可为0.10‐0.20 U/kg/天。没有合适的证据来研究基础胰岛素的最佳维持剂量。

**结论:**5种基础胰岛素对目标人群有效。与NPH相比, 甘精胰岛素U‐300、德谷胰岛素U‐100、甘精胰岛素U‐100和地特胰岛素在不影响血糖控制的情况下可减少低血糖事件。

## 1 INTRODUCTION

Type 2 diabetes mellitus (T2DM) is a disease associated with a state of chronic hyperglycemia that results in a significant increase in the risk of microvascular and macrovascular complications, and eventually, leads to diabetes‐related mortality.^1^ About 537 million individuals globally were affected by diabetes mellitus in 2021, and T2DM accounted for over 90% of these cases.^2^ Thus, appropriately controlling glucose and at the same time avoiding hypoglycemia is essential for patients with T2DM, as it is linked to their quality of life in the long run. When hyperglycemia cannot be properly controlled, injectable glucose‐lowering therapy such as glucagon‐like peptide‐1 receptor agonist or basal insulin is added. To date, there are five common basal insulins available for use: glargine U‐300, degludec U‐100, glargine U‐100, detemir, and neutral protamine hagedorn (NPH) insulin. After a literature search for English publications, there is no systematic‐review‐based clinical practice guideline to date on these five basal insulin regimens for adult T2DM insulin‐naïve patients with inadequately controlled glucose (ie, glycated hemoglobin [HbA1c] > 7.0%) treated with one or more oral glucose‐lowering drugs in the Asian‐Pacific region. Therefore, the authors aim to fill this knowledge gap by focusing on answering the following questions:

Q1. What are the differences in the effectiveness and safety among five basal insulin regimens after the initiation of insulin therapy in the target population?

Q2. What are the optimal starting dose (U/kg/day) and time of administration (morning vs. bedtime) of each basal insulin?

Q3. Among the target population, what is the optimal maintenance dose (U/kg/day) that achieves target fasting plasma glucose (FPG)?

Q4. After initiation of any of the five basal insulins, what range of target FPG can lead to the ideal HbA1c level (ie, <7.0%) in the target population?

After a quick PubMed search, we found three relevant systematic reviews.^3^, ^4^, ^5^ However, none of them answered all four questions. Therefore, a new systematic review was worthwhile.

## 2 METHODS

### 2.1 Search strategy and selection criteria

This systematic review followed the Preferred Reporting Items for Systematic Reviews and Meta‐Analyses (PRISMA) reporting checklist.^6^ MEDLINE, EMBASE, Web of Science, and the Cochrane Library were searched from January 2000 to February 2022. Search terms included the names of the five basal insulins, T2DM, randomized controlled trials (RCTs), and combinations with their alternatives, respectively (Supplementary Table S1). One reviewer screened the references retrieved. A second reviewer audited and resolved any disagreements with a third reviewer. The protocol was registered in PROSPERO (CRD42022319078**)**.

#### 2.1.1 Inclusion Criteria

The study recruited adult insulin‐naïve patients with T2DM who required basal insulin therapy and had ≥1 oral glucose lowering drug; was an RCT; had a treatment duration of ≥12 weeks; reported any of the following outcomes: HbA1c, incidence of hypoglycemia, incidence of severe hypoglycemia, incidence of nocturnal hypoglycemia, time in range, FPG, cost‐effectiveness, weight gain, patient‐reported outcomes (eg, quality of life); and was published after 1 January 2019 for a conference abstract.

#### 2.1.2 Exclusion Criteria

The study focused on pregnant women, investigated basal insulin that was initiated twice daily, was published in a language other than English, or was a non‐RCT.

### 2.2 Data analysis

One reviewer extracted data, which was subsequently audited by an independent auditor. The risk of bias per outcome for each included study was assessed using the Cochrane Collaboration Risk of Bias 2.0 tools.^7^

We used RevMan 5.3 to analyze the data. For binary outcomes, we calculated the risk ratio (RR) and its 95% confidence interval (CI). For continuous outcomes, where possible, we calculated mean difference (MD) with its 95% CI. When clinically and methodologically homogeneous results from two or more studies were available, a meta‐analysis was conducted. We employed I^2^ > 50% as a general guide to identify statistical heterogeneity in the pooled analysis.

The certainty of the evidence per outcome for each comparison, considering the risk of bias, inconsistency, indirectness, imprecision, and publication bias, was assessed using the Grading of Recommendations, Assessment, Development, and Evaluation (GRADE) approach.^8^

## 3 RESULTS

### 3.1 Literature search

**FIGURE 1:**
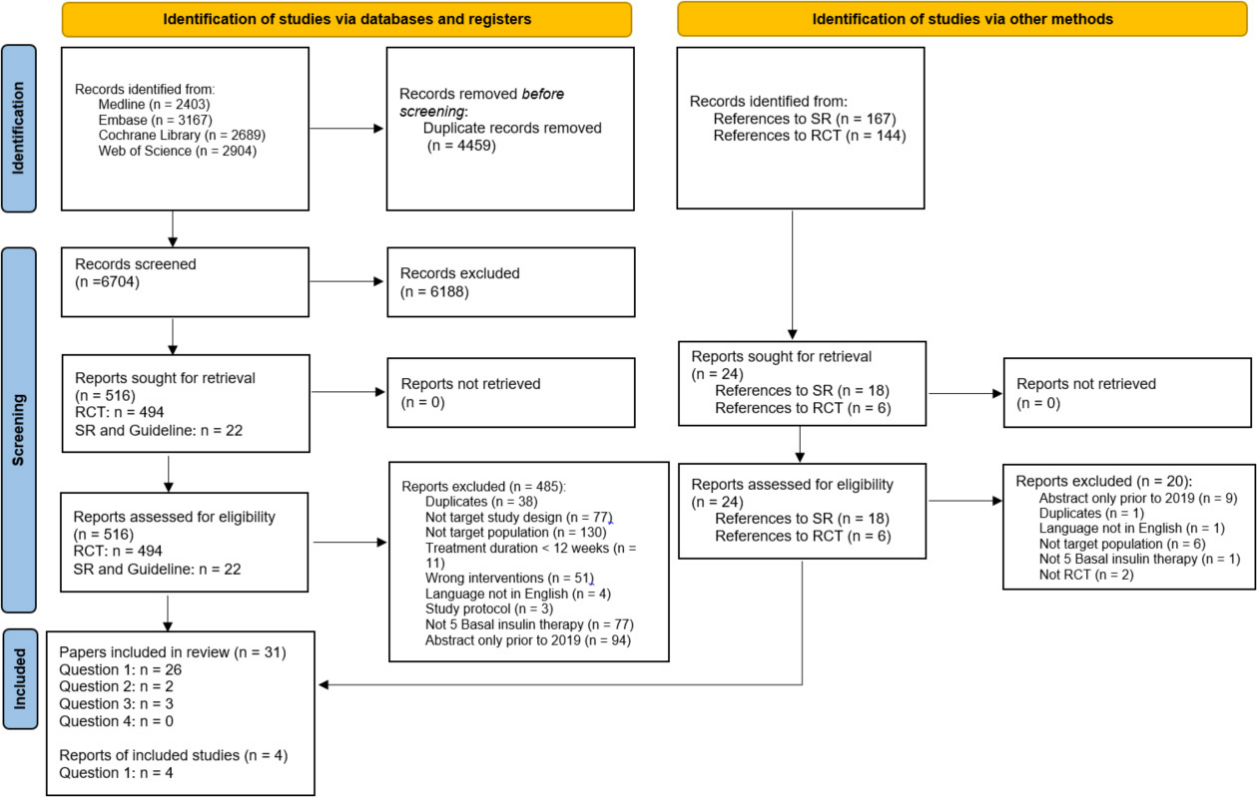
PRISMA flow diagram. PRISMA, Preferred Reporting Items for Systematic Reviews and Meta‐Analyses; RCT, randomized controlled trial; SR, systematic review.

Overall, we retrieved 11 163 publications. After reviewing titles and abstracts, 515 references were deemed eligible for full‐text screening. Eventually, 35 publications (31 RCTs) met the planned inclusion criteria.^9^, ^10^, ^11^, ^12^, ^13^, ^14^, ^15^, ^16^, ^17^, ^18^, ^19^, ^20^, ^21^, ^22^, ^23^, ^24^, ^25^, ^26^, ^27^, ^28^, ^29^, ^30^, ^31^, ^32^, ^33^, ^34^, ^35^, ^36^, ^37^, ^38^, ^39^, ^40^, ^41^, ^42^, ^43^ Among them, 30 papers answered Q1,^11^, ^13^, ^14^, ^15^, ^16^, ^17^, ^18^, ^19^, ^20^, ^21^, ^22^, ^23^, ^24^, ^26^, ^27^, ^28^, ^29^, ^30^, ^31^, ^33^, ^34^, ^35^, ^36^, ^37^, ^38^, ^39^, ^40^, ^41^, ^42^, ^43^ 2 papers answered Q2,^10^, ^12^ and 3 papers answered Q4.^9^, ^25^, ^32^ There were no eligible studies answering Q3 directly. The characteristics of these included studies are listed in Tables 1, 2, 3 for Q1, Q2, and Q4, respectively. A PRISMA flow diagram with reasons for study exclusion is shown in Figure 1.^6^

**TABLE 1:** The trials and patients' characteristics for Research Question 1.

Author year (Trial name if available); Country	Intervention	Comparator	Mean/median (±SD/range) Age (years)	Renal function eGFR (mL/min/1.73 m^2^)	I (N)/ C(N)	Trial Duration (weeks)	Mean/median (±SD/range) Duration of Diabetes (years)	Mean/median (±SD/range) HbA1c (%)	Mean/median (±SD/range) BMI (Kg/m^2^)	Mean/median (±SD/range) FPG (mmol/L)	Mean/median (±SD/range) body weight (kg)
1. One insulin bedtime vs. another insulin bedtime injection											
(1) Glargine U‐300 vs. degludec U‐100											
Bolli 2021, Cheng 2020, Rosenstock 2018, Haluzik 2020 (BRIGHT)^15^, ^16^, ^18^, ^21^; USA, Bulgaria, Croatia, Czechia, Denmark, France, Greece, Hungary, Israel, Italy, Romania, Serbia, Slovakia, Sweden, Switzerland, and UK	Glargine U‐300	Degludec U‐100	I: 60.6 ± 6.4; C: 60.5 ± 6.3	I: 92.4 ± 26.8; C: 90.8 ± 26.0	I: 466; C: 463	12 weeks; 24 weeks	I: 10.5 ± 6.1; C: 10.7 ± 6.5	I: 8.7 ± 0.8; C: 8.6 ± 0.8	I: 31.7 ± 4.3; C: 31.3 ± 4.4	NR	I: 90.6 ± 16.1; C: 88.7 ± 15.9
(2) Degludec U‐100 vs. glargine U‐100											
Onishi 2013^34^; Hong Kong, Japan, Malaysia, South Korea, Taiwan, and Thailand	Degludec U‐100	Glargine U‐100	I: 58.8 ± 9.8; C: 58.1 ± 10.1	Serum creatinine (mol/L): I: 78 ± 18; C: 77 ± 18	I: 289; C: 146	26 weeks	I: 11.8 ± 6.5; C: 11.1 ± 6.5	I: 8.4 ± 0.8; C: 8.5 ± 0.8	I: 24.6 ± 3.4; C: 25.8 ± 3.7	I: 8.4 ± 2.1; C: 8.6 ± 1.9	I: 64.9 ± 11.5; C: 67.4 ± 11.6
Pan 2016^37^; Brazil, Canada, China, South Africa, Ukraine, and USA	Degludec U‐100	Glargine U‐100	I: 55.9 ± 9.7; C: 56.6 ± 9.2	NR	I: 555; C: 278	26 weeks	I: 7.6 ± 5.3; C: 8.3 ± 5.5	I: 8.3 ± 0.9; C: 8.3 ± 0.8	I: 27.4 ± 4.7; C: 27.0 ± 4.6	I: 9.4 ± 2.4; C: 9.4 ± 2.5	I: 75.5 ± 15.6; C: 73.8 ± 16.1
Zinman 2012 (BEGIN Once Long)^22^; Austria, Belgium, Canada, Czech Republic, Denmark, Finland, France, Germany, Norway, Serbia and Montenegro, Spain, and USA	Degludec U‐100	Glargine U‐100	I: 59.3 ± 9.7; C: 58.7 ± 9.9	NR	I: 773; C: 257	52 weeks	I: 9.4 ± 6.3; C: 8.6 ± 5.7	I: 8.2 ± 0.8; C: 8.2 ± 0.8	I: 30.9 ± 4.8; C: 31.6 ± 4.4	I: 9.6 ± 2.6; C: 9.7 ± 2.6	I: 89.4 ± 17.7; C: 91.8 ± 15.8
(3) Glargine U‐300 vs. glargine U‐100											
Bolli 2015, Bolli 2017 (EDITION 3)^20^, ^35^; North America, Europe, and Japan	Glargine U‐300	Glargine U‐100	I: 58.2 ± 9.9; C: 57.2 ± 10.3	I: 81.3 ± 19.6; C: 80.7 ± 19.9	I: 439; C: 439	24 weeks; 48 weeks	I: 10.1 ± 6.5; C: 9.6 ± 6.2	I: 8.5 ± 1.0; C: 8.6 ± 1.1	I: 32.8 ± 6.9; C: 33.2 ± 6.6	I: 9.9 ± 2.9; C: 10.2 ± 2.9	NR
Ji 2020 (EDITION AP)^17^; China, South Korea, and Taiwan	Glargine U‐300	Glargine U‐100	I: 58.5 ± 9.6; C: 57.9 ± 10.2	I: 90.4 ± 20.5; C: 88.7 ± 20.7	I: 401; C: 203	26 weeks	I: 10.7 ± 6.4; C: 10.5 ± 5.8	I: 8.6 ± 0.9; C: 8.5 ± 1.0	I: 25.2 ± 3.2; C: 25.3 ± 3.2	I: 9.9 ± 2.3; C: 9.7 ± 2.2	NR
(4) Glargine U‐300 vs. NPH											
Ling 2021^11^; Hong Kong and China	Glargine U‐300	NPH	I: 57.5 ± 10.9； C: 59.2 ± 13.8	I: 76 ± 27; C: 86 ± 20	I: 24; C: 25	24 weeks	I: 14 ± 8; C: 13 ± 5	I: 8.9 ± 1.0； C: 9.0 ± 1.1	I: 25.6 ± 4.3; C: 24.1 ± 3.5	I: 9.71 ± 2.58; C: 9.40 ± 1.64	I: 68.4 ± 12.6; C: 63.6 ± 12.9
(5) Glargine U‐100 vs. Detemir											
Rosenstock 2008^38^; Europe and USA	Glargine U‐100	Detemir	I: 59.4 ± 9.6; C: 58.4 ± 10.2	NR	I: 291; C: 291	52 weeks	I: 9.1 ± 6.4; C: 9.1 ± 6.1	I: 8.6 ± 0.8; C: 8.6 ± 0.8	I: 30.5 ± 4.6; C: 30.6 ± 4.8	I: 10.8; C: 10.8	I: 87.4 ± 16.6; C: 87.4 ± 17.4
Elisha 2015^30^; Canada	Glargine U‐100	Detemir	I: 60.2 ± 1.6; C: 58.2 ± 3.5	NR	I: 21; C: 21	24 weeks	I: 10.5 ± 5.4; C: 10.2 ± 4.6	I: 9.1 ± 1.0; C: 8.7 ± 0.8	I: 32.8 ± 4.6; C: 31.5 ± 4.7	I: 11.2 ± 3.0; C: 11.1 ± 3.1	I: 91.8 ± 16.3; C: 90.0 ± 17.6
Meneghini 2013; Argentina, India, Republic of Korea, Thailand, and USA	Glargine U‐100	Detemir	I: 57.3 ± 10.3; C: 57.3 ± 10.2	NR	I: 227; C: 226	26 weeks	I: 8.4 ± 6.6; C: 8.0 ± 5.6	I: 7.86 ± 0.58; C: 7.96 ± 0.62	I: 29.1 ± 3.9; C: 28.9 ± 4.0	I: 8.46 ± 2.21; C: 8.66 ± 2.26	I: 81.7 ± 16.2; C: 82.8 ± 17.2
(5.1.) Glargine U‐100 NR vs. detemir NR injection time											
Cander 2014^40^; Turkey	Glargine U‐100	Detemir	I: 57 (41–70); C: 59 (40–69)	NR	I: 22; C: 20	12 weeks	I: 6.5 (1–10); C: 7.0 (1–10)	I: 9.9 (7.7–12.0); C: 9.6 (7.3–12.0)	I: 28.7 (22–40); C: 30.1 (21–37)	NR	I: 79.5 (61–113); C: 75.5 (50–130)
(6) Glargine U‐100 vs. NPH											
Hermanns 2015^31^; Germany	Glargine U‐100	NPH	I: 61.9 ± 8.8; C: 62.7 ± 9.2	NR	I: 176; C: 167	24 weeks	I: 9.6 ± 5.9; C: 9.6 ± 5.9	I: 8.2 ± 0.7; C: 8.1 ± 0.7	I: 30.9 ± 4.5; C: 31.2 ± 4.7	I: 9.2 ± 2.2; C: 9.5 ± 2.2	I: 90.1 ± 15.8; C: 91.1 ± 15.1
Yki‐Jarvinen 2006^39^; Finland and UK	Glargine U‐100	NPH	I: 56 ± 1(SE); C: 57 ± 1(SE)	NR	I: 61; C: 49	36 weeks	I: 9 ± 1 (SE); C: 9 ± 1 (SE)	I: 9.5 ± 0.1 (SE); C: 9.6 ± 0.1 (SE)	I: 31.3 ± 0.7 (SE); C: 32.0 ± 0.8 (SE)	I: 13.0 ± 0.3 (SE); C: 12.9 ± 0.3 (SE)	I: 92.0 ± 2.4; C: 94.4 ± 2.6
Pan 2007^14^; China, Hong Kong, Indonesia, South Korea, Malaysia, Pakistan, Philippines, Taiwan, Thailand, and Singapore	Glargine U‐100	NPH 24	I: 55.6 ± 8.4; C: 56.6 ± 8.7	NR	I: 220; C: 233	24 weeks	I: 10.3 ± 6.3; C: 10.0 ± 5.4	I: 9.0 ± 0.9; C: 9.0 ± 0.8	I: 24.8 ± 3.1; C: 25.1 ± 3.3	mg/dL: I: 226 ± 51; C: 223 ± 53	NR
Forst 2010^19^; Germany	Glargine U‐100	NPH	I: 66.9 ± 6.2; C: 58.0 ± 8.5	NR	I: 15; C: 15	12 weeks	I: 11.6 ± 7.5; C: 8.6 ± 4.7	I: 7.1 ± 0.6; C: 7.1 ± 0.4	I: 30.0 ± 3.7; C: 31.5 ± 4.9	NR	NR
Riddle 2003^23^; USA and Canada	Glargine U‐100	NPH	I: 55 ± 9.5; C: 56 ± 8.9	NR	I: 367; C: 389	24 weeks	I: 8.4 ± 5.6; C: 9.0 ± 5.6	I: 8.6 ± 0.9; C: 8.6 ± 0.9	I: 32.5 ± 4.6; C: 32.2 ± 4.8	mg/dL: I: 198 ± 49; C: 194 ± 47	NR
Fritsche 2003^26^; European countries	Glargine U‐100	NPH	I: 60 ± 9; C: 62 ± 9	NR	I: 229; C: 234	24 weeks	I: 8.2 (1–51); C: 9.3 (1–39)	I: 9.1 ± 1.0; C: 9.1 ± 1.1	I: 28.7 ± 3.9; C: 28.9 ± 3.9	I: 12.0 ± 2.9; C: 12.2 ± 3.2	I: 82.1 ± 13.6; C: 81.0 ± 14.9
Eliaschewitz 2006^27^; Argentina, Brazil, Chile, Colombia, Guatemala, Mexico, Paraguay, Peru, Urugua,y and Venezuela	Glargine U‐100	NPH	I: 56.1 ± 9.9; C: 57.1 ± 9.6	NR	I: 231; C: 250	24 weeks	I: 10.3 ± 6.4; C: 10.8 ± 6.4	I: 9.1 ± 1.0; C: 9.2 ± 0.9	I: 27.3 ± 3.7; C: 27.2 ± 4.0	I: 11.4 ± 3.2; C: 10.7 ± 3.1	NR
Oikonomou 2014^29^; Germany	Glargine U‐100	NPH	I: 60.1 ± 7.3; C: 61.5 ± 5.0	Serum creatinine (mg/dl): I: 0.9 ± 0.2; C: 0.8 ± 0.2	I: 20; C: 22	16 weeks	I: 8.7 ± 6.6; C: 9.8 ± 7.2	I: 7.3 ± 0.9; C: 7.5 ± 0.7	I: 32.7 ± 6.0; C: 31.8 ± 5.2	mg/dL: I: 166.8 ± 50.1; C: 165.9 ± 38.9	NR
Benedetti 2003^36^; European countries and South Africa	Glargine U‐100	NPH	I: 59.3 ± 9.3; C: 59.3 ± 9.0	NR	I: 222; C: 204	52 weeks	I: 9.9 ± 6.1; C: 10.2 ± 6.2	I: 9.1 ± 1.2; C: 8.9 ± 1.1	I: 29.3 ± 4.4; C: 28.5 ± 4.1	I: 12.9 ± 3.2; C: 13.1 ± 2.8	NR
Yki‐Jarvinen 2000^24^; Germany and Finland	Glargine U‐100	NPH	I: 59 ± 1(40–80); C: 59 ± 1(40–80)	NR	I: 214; C: 208	52 weeks	I: 10.0 ± 1.0; C: 10.0 ± 1.0	I: 9.1 ± 0.1; C: 8.9 ± 0.1	I: 29.3 ± 0.3; C: 28.5 ± 0.3	NR	NR
Hsia 2011^42^; USA	Glargine U‐100	NPH	I: 50.3 ± 11.2; C: 53.2 ± 7.7	NR	I: 30; C: 30	26 weeks	I: 9.0 ± 5.9; C: 7.8 ± 4.2	I: 9.2 ± 1.3; C: 9.3 ± 1.6	I: 31.6 ± 5.0; C: 32.1 ± 6.0	mg/dL: I: 189 ± 60; C: 175 ± 48	I: 85.0 ± 15.0; C: 82.6 ± 18.1
Home 2015^41^; NCT00949442; Europe, Asia, the Middle East, and South America	Glargine U‐100	NPH	I: 57.3 ± 8.3; C: 57.2 ± 7.8	NR	I: 352; C: 349	36 weeks	I: 9.1 ± 5.5; C: 9.4 ± 5.7	I: 8.2 ± 0.8; C: 8.2 ± 0.9	I: 29.7 ± 4.5; C: 30.1 ± 4.5	I: 9.2 ± 2.1; C: 9.0 ± 2.0	I: 81.2 ± 16.0; C: 82.7 ± 15.5
(7) Detemir vs. NPH											
Tsimikas 2006^28^; Denmark, France, Italy, The Netherlands, Norway, Spain, and USA	Detemir	NPH	I: 58.7 ± 10.2; C: 58.4 ± 11.0	NR	I: 169; C: 164	20 weeks	I: 10.5 ± 7.0; C: 10 ± 6.9	I: 8.9 ± 1.0; C: 9.2 ± 1.0	I: 29.7 ± 5.1; C: 30.4 ± 4.8	I: 10.8 ± 2.8; C: 11.5 ± 2.9	NR
2 One insulin morning vs. another insulin morning injection											
(1) Degludec U‐100 vs. glarginge U‐100											
Aso 2017^13^; Japan	Degludec U‐100	Glargine U‐100	I: 64.0 ± 13.6; C: 64.7 ± 15.7	NR	I: 32; C: 12	24 weeks	I: 10.0 (6.0, 20.0); C: 13.0 (7.0, 25.0)	I: 8.9 ± 1.5; C: 8.8 ± 1.5	I: 24.8 ± 4.1; C: 24.3 ± 4.8	NR	I: 62.2 ± 11.7; C: 60.3 ± 12.3
(2) Detemir vs. NPH											
NR (The 3 L study)^33^; France and UK	Detemir	NPH	I: 77.6 ± 5.5; C: 76.1 ± 4.9	NR	I: 38; C: 48	28 weeks	I: 14.1 (0–42); C: 14.1 (0–42)	I: 9.3 ± 0.9; C: 9.1 ± 0.8	I: 29.1 ± 4.6; C: 29.8 ± 5.5	NR	I: 77.4 ± 15.9; C: 80.4 ± 16.4
Hsia 2011^42^; USA	Glargine U‐100	NPH	I: 53.0 ± 8.6; C: 53.2 ± 7.7	NR	I: 25; C: 30	26 weeks	I: 9.5 ± 5.2; C: 7.8 ± 4.2	I: 9.6 ± 1.2; C: 9.3 ± 1.6	I: 31.1 ± 5.2; C: 32.1 ± 6.0	mg/dL: I: 174 ± 59; C: 175 ± 48	I: 82.7 ± 14.3; C: 82.6 ± 18.1
3. One insulin morning vs another insulin bedtime injection											
(1) Detemir morning time vs. NPH bedtime											
Tsimikas 2006^28^; Denmark, France, Italy, The Netherlands, Norway, Spain, and USA	Detemir	NPH	I: 58.3 ± 10.4; C: 58.4 ± 11	NR	I: 165; C: 164	20 weeks	I: 10.5 ± 7.6; C: 10 ± 6.9	I: 9.1 ± 1.0; C: 9.2 ± 1.0	I: 29.8 ± 5.0; C: 30.4 ± 4.8	I: 11.5 ± 2.7; C: 11.5 ± 2.9	NR
(2) Glargine U‐100 morning time vs. NPH bedtime											
Fritsche 2003^26^; European countries	Glargine U‐100	NPH	I: 61 ± 9; C: 62 ± 9	NR	I: 237; C: 234	24 weeks	I: 9.0 (0–38); C: 9.3 (1–39)	I: 9.1 ± 1.0; C: 9.1 ± 1.1	I: 28.6 ± 4.5; C: 28.9 ± 3.9	I: 12.1 ± 3.0; C: 12.2 ± 3.2	I: 80.7 ± 15.8; C: 81.0 ± 14.9
4 One insulin morning time vs. its bedtime injection											
(1) Detemir											
Tsimikas 2006^28^; Denmark, France, Italy, The Netherlands, Norway, Spain, and USA	Detemir	Detemir	I: 58.3 ± 10.4; C: 58.7 ± 10.2	NR	I: 165; C: 169	20 weeks	I: 10.5 ± 7.6; C: 10.5 ± 7.0	I: 9.1 ± 1.0; C: 8.9 ± 1.0	I: 29.8 ± 5.0; C: 29.7 ± 5.1	I: 11.5 ± 2.7; C: 10.8 ± 2.8	NR
(2) Glargine U‐100											
Fritsche 2003^26^; European countries	Glargine U‐100	Glargine U‐100	I: 61 ± 9; C: 60 ± 9	NR	I: 237; C: 229	24 weeks	I: 9.0 (0–38); C: 8.2 (1–51)	I: 9.1 ± 1.0; C: 9.1 ± 1.0	I: 28.6 ± 4.5; C: 28.7 ± 3.9	I: 12.1 ± 3.0; C: 12.0 ± 2.9	I: 81.0 ± 14.9; C: 82.1 ± 13.6
Hsia 2011^42^; USA	Glargine U‐100	Glargine U‐100	I: 53.0 ± 8.6; C: 50.3 ± 11.2	NR	I: 25; C: 30	26 weeks	I: 9.5 ± 5.2; C: 9.0 ± 5.9	I: 9.6 ± 1.2; C: 9.2 ± 1.3	I: 31.1 ± 5.2; C: 31.6 ± 5.0	mg/dL: I: 174 ± 59; C: 189 ± 60	I: 82.7 ± 14.3; C: 85.0 ± 15.0

Abbreviations: BMI, body mass index; C, comparator; eGFR, estimated glomerular filtration rate; FPG, fasting plasma glucose; HbA1c, glycated hemoglobin; I, intervention; NPH, insulin neutral protamine hagedorn; NR, not reported.

**TABLE 2:** The trials and patients' characteristics for Research Question 2.

Author year (Trial name if available); Country	Mean ± SD age (years)	Intervention	Comparator	Renal function	Intervention (N)/comparator (N)	Trial duration (weeks)	Mean ± SD duration of diabetes (years)	Mean/median (±SD/range) HbA1c (%)	Mean/median (±SD/range) BMI (Kg/m^2^)	Mean/median (±SD/range) FPG (mmol/L)	Mean/median (±SD/range) body weight (kg)
Detemir injection time unknown											
Cander 2014^10^; Turkey	I: 56.2 ± 7.1; C: 54.8 ± 8.0	0.12 U/kg Bid	0.12 U/kg/day	NR	I: 30; C: 30	12 weeks	I: 6.7 ± 2.6; C: 7.2 ± 3.1	I: 9.8 ± 1.3%; C: 9.3 ± 1.2%	I: 29.4 ± 5.0; C: 28.5 ± 3.8	NR	I: 78.3 ± 16.6; C: 83.2 ± 12.1;
Initial dose of Glargine U‐100 injected at bedtime											
Ji 2020^12^; (BEYOND VII); Mainland China	I: 53.2 ± 9.6; C: 51.7 ± 9.7	0.2 U/kg/day	0.3 U/kg/day	NR	I: 444; C: 448	16 weeks	I: 17.9 ± 4.5; C: 7.3 ± 4.4	I: 8.8 ± 1.0%; C: 8.8 ± 1.0%	I: 27.8 ± 2.7; C:27.8 ± 2.6	I: 11.6 ± 2.7; C: 11.5 ± 2.8	I: 76.7 ± 11.3; C: 77.5 ± 11.2

Abbreviations: Bid, twice daily; BMI, body mass index; C, comparator; FPG, fasting plasma glucose; HbA1c, glycated hemoglobin; I, intervention; NR, not reported.

**TABLE 3:** The trials and patients' characteristics for Research Question 4.

Author year (trial name if available); country	Meanm/Median (±SD/range) Age (years)	Renal function eGFR (mL/min/1.73 m^2^)	I (N)/ C(N)	Trial duration (weeks)	Mean ± SD duration of diabetes years/months)	Mean/median (±SD/range) HbA1c (%)	Mean/median (±SD/range) BMI (Kg/m^2^)	Mean/median (±SD/range) FPG (mmol/L)	Mean/median (±SD/range) body weight (kg)
Glargine U‐100 injected at bedtime or unknown (3.9 < FPG ≤5.6 mmol/L vs. 3.9 < FPG ≤6.1 mmol/L)									
Yuan 2021^9^; China	I: 56.5 ± 8.9; C: 57.9 ± 4.7	Creatinine (mmol/L): I: 62.5 ± 13.0; C: 59.0 ± 14.7	I: 10; C: 26	24 weeks	I: 103.1 ± 66.4 months; C: 113.6 ± 62.8 months	I: 139.4 ± 10.5; C: 143.6 ± 10.0	I: 25.7 ± 3.1; C: 24.6 ± 3.0	I: 8.3 ± 0.9; C: 8.6 ± 1.9	I: 68.8 ± 10.2; C: 65.2 ± 12.6
Yang 2019^25^; China	I: 54.1 ± 7.2; C: 54.2 ± 7.4	NR	I: 136; C: 405	24 weeks	I: 8.2 ± 5.5; C: 8.0 ± 4.7	I: 8.50 ± 0.91; C: 8.63 ± 0.92	I: 25.5 ± 3.0; C: 25.6 ± 3.0	I: 10.4 ± 2.2; C: 10.6 ± 2.2	I: 69.6 ± 11.6; C: 70.5 ± 11.7
Glargine U‐100 injected at bedtime or unknown (3.9 < FPG ≤6.1 mmol/L vs. 3.9 < FPG ≤7.0 mmol/L)									
Yuan 2021^9^; China	I: 57.9 ± 4.7; C: 53.8 ± 7.3	Creatinine (mmol/L): I: 59.0 ± 14.7; C: 65.0 ± 12.2	I: 26; C: 35	24 weeks	I: 113.6 ± 62.8 months; C: 107.9 ± 65.1 months	I: 143.6 ± 10.0; C: 144.0 ± 11.3	I: 24.6 ± 3.0; C: 25.6 ± 2.4	I: 8.6 ± 1.9; C: 8.3 ± 1.6	I: 65.2 ± 12.6; C: 70.9 ± 9.5
Yang 2019^25^; China	I: 54.2 ± 7.4; C: 53.5 ± 7.4	NR	I: 405; C: 406	24 weeks	I: 8.0 ± 4.7; C: 7.8 ± 4.8	I: 8.6 ± 0.9; C: 8.6 ± 0.9	I: 25.6 ± 3.0; C: 25.6 ± 3.2	I: 10.6 ± 2.2; C: 10.5 ± 2.3	I: 70.5 ± 11.7; C: 70.1 ± 11.2
Glargine U‐100 injected at bedtime or unknown (3.9 < FPG ≤5.6 mmol/L vs. 3.9 < FPG ≤7.0 mmol/L)									
Yuan 2021^9^; China	I: 56.5 ± 8.9; C: 53.8 ± 7.3	Creatinien (mmol/L): I: 62.5 ± 13.0; C: 65.0 ± 12.2	I: 10; C: 35	24 weeks	I: 103.1 ± 66.4 months; C: 107.9 ± 65.1 months	I: 139.4 ± 10.5; C: 144.0 ± 11.3	I: 25.7 ± 3.1; C: 25.6 ± 2.4	I: 8.3 ± 0.9; C: 8.3 ± 1.6	I: 68.8 ± 10.2; C: 70.9 ± 9.5
Yang 2019^25^; China	I: 54.1 ± 7.2; C: 53.5 ± 7.4	NR	I: 136; C: 406	24 weeks	I: 8.2 ± 5.5; C: 7.8 ± 4.8	I: 8.50 ± 0.91; C: 8.57 ± 0.94	I: 25.5 ± 3.0; C: 25.6 ± 3.2	I: 10.4 ± 2.2; C: 10.5 ± 2.3	I: 69.6 ± 11.6; C: 70.1 ± 11.2
Detemir injected at bedtime (3.9 < FPG ≤5.0 mmol/L vs. 4.4 < FPG ≤6.1 mmol/L)									
Blonde 2009^32^; (TITRATE™); USA	I: 56.6; C: 57.2	NR	I: 122; C: 122	20 weeks	I: 7.9; C: 9.0	I: 8.0; C: 7.9	I: 33; C: 33.6	I: 9.1; C: 9.1	I: 95.9; C: 98.6

Abbreviations: BMI, body mass index; C, comparator; eGFR, estimated glomerular filtration rate; FBG, fasting blood glucose; FPG, fasting plasma glucose; HbA1c, glycated hemoglobin; I, intervention; NR, not reported.

### 3.2 Risk of bias assessment and evidence certainty

Results of the risk of bias assessment for the 35 studies are shown in Supplementary Table S2. Overall, the risk of bias ranged from “some concerns” to “high risk” in the analysis in Q1 and Q4, and “some concerns” in Q2. The certainty of evidence for each comparison of interventions was high to low after considering four other factors from the GRADE approach (Supplementary Table S3). The clinical thresholds defined as small, moderate, and large effects for relevant outcomes were made by the working group members based on their clinical experience for judgment of imprecision of certainty of evidence and balancing the effect magnitudes of desirable and undesirable outcomes based on the GRADE approach (listed in Supplementary Table S4).^44^

## 4 Q1. EFFECTIVENESS AND SAFETY AMONG FIVE BASAL INSULIN REGIMENS

Thirty papers (26 RCTs) were eligible for this research question.^11^, ^13^, ^14^, ^15^, ^16^, ^17^, ^18^, ^19^, ^20^, ^21^, ^22^, ^23^, ^24^, ^26^, ^27^, ^28^, ^29^, ^30^, ^31^, ^33^, ^34^, ^35^, ^36^, ^37^, ^38^, ^39^, ^40^, ^41^, ^42^, ^43^ Based on different administration times of basal insulins, we classified these RCTs into four categories: (1) one basal insulin injected at bedtime versus another basal insulin injected at bedtime; (2) one basal insulin injected at morning time versus another basal insulin injected at morning time. (3) one basal insulin injected at morning time versus another basal insulin injected at bedtime; and (4) one basal insulin injected at morning time versus the same insulin injected at bedtime. All results of the relative and absolute effects between any comparison are shown in Appendix Table S3.

### 4.1 One basal insulin injected at bedtime versus another basal insulin injected at bedtime

#### 4.1.1 Glargine U‐300 versus Degludec U‐100

The BRIGHT trial with 924 intention‐to‐treat patients provided data for this comparison.^18^, ^21^ Evidence on the following critical outcomes slightly favored glargine U‐300, including a higher percentage of patients with HbA1c < 7.0% at 6 months (40 more/1000 patients, 95% CI 22 fewer−111 more); a lower percentage of patients experienced hypoglycemia <3.0 mmol/L at 3 months (39 fewer/1000, 95% CI 64 fewer–0 fewer) and at 6 months 37 (fewer per 1000, 95% CI 74 fewer–13 fewer); and hypoglycemia <3.9 mmol/L at 3 months (71 fewer/1000, 95% CI 125 fewer−5 fewer). But nocturnal hypoglycemia <3.9 mmol/L at 3 months (36 more/1000, 95% CI 11 fewer−100 more) favored degludec U‐100. Except for these outcomes, the results of other critical outcomes at three or 6 months are similar between two insulins, such as HbA1c change and nocturnal hypoglycemia <3.0 mmol/L at 3 months; severe hypoglycemia, nocturnal hypoglycemia <3.0 mmol/L, and nocturnal hypoglycemia <3.9 mmol/L at 6 months, etc.

Subgroup analyses of the BRIGHT trial by age (<65 years and ≥ 65 years) showed consistent results as described,^15^ whereas subgroup analyses by renal function (<60, ≥60 but <90, ≥90 mL/min/1.73 m^2^) showed heterogeneity of treatment effects.^16^ For patients with estimated glomerular filtration rate (eGFR)≥90 mL/min/1.73 m^2^, the results from critical outcomes were consistent with the overall results in the BRIGHT trial. Degludec U‐100 induced less harm in patients with eGFR 60–90 mL/min/1.73 m^2^ (*n* = 365) on nocturnal hypoglycemia <3.0 mmol/L at 6 months (28 more/1000, 95% CI 12 fewer−125 more), and nocturnal hypoglycemia <3.9 mmol/L at 6 months (64 more/1000, 95% CI 24 fewer−186 more) with similar results of HbA1c. The data from patients with eGFR <60 mL/min/1.73 m^2^ indicated that the incidence of nocturnal hypoglycemia slightly favored degludec U‐100 but the results of event rate/patient‐year for nocturnal hypoglycemia slightly favored glargine U‐300, and HbA1c change at 6 months was 0.43% lower (95% CI 0.74% lower to 0.12% lower) favored glargine U‐300.

#### 4.1.2 Degludec U‐100 versus Glargine U‐100

Three RCTs with 2298 patients provided data for this comparison.^22^, ^34^, ^37^ The main evidence favored degludec U‐100 for less severe hypoglycemia at 12 months (17 fewer/1000, 95% CI 19 fewer –6 fewer), less nocturnal hypoglycemia <3 mmol/L at 6 months (24 fewer/1000, 95% CI 54 fewer−17 more), and less hypoglycemia <3.0 mmol/L at 6 months (41 fewer/1000, 95% CI 89 fewer−11 more). The evidence favored glargine U‐100 for HbA1c<7.0% at 6 months (20 fewer/1000, 95% CI 116 fewer−459 more) and HbA1c<7.0% at 12 months (22 fewer/1000, 95% CI 87 fewer−49 more).

#### 4.1.3 Glargine U‐300 versus Glargine U‐100

Three RCTs with 1482 patients provided data for this comparison.^17^, ^20^, ^35^ Evidence on the following outcomes favored glargine U‐300 including HbA1c < 7.0% at 12 months (54 more/1000, 95% CI 5 fewer –127 more), less hypoglycemia <3.0 mmol/L at 12 months (82 fewer/1000, 95% CI 117 fewer –35 fewer), less hypoglycemia <3.0 mmol/L at 3 months (118 fewer/1000, 95% CI 187 fewer−41 fewer), less nocturnal hypoglycemia <3.9 mmol/L at 3 months (110 fewer/1000, 95% CI 163 fewer−40 fewer), less hypoglycemia <3.9 mmol/L at 6 months (53 fewer/1000, 95% CI 94 fewer−6 fewer), less nocturnal hypoglycemia <3.9 mmol/L at 6 months (61 fewer/1000, 95% CI 96 fewer−19 fewer), and less nocturnal hypoglycemia <3.9 mmol/L at 12 months (41 fewer/1000, 95% CI 90 fewer−20 more). But glargine U‐100 produced greater benefit on nocturnal hypoglycemia <3.0 mmol/L at 3 months (5 more/1000, 95% CI 14 fewer−60 more) relative to glargine U‐300.

#### 4.1.4 Glargine U—300 versus NPH

One RCT with 50 patients was included in this comparison.^11^ Evidence on the following outcomes favored glargine: U‐300 including less hypoglycemia <3.0 mmol/L at 6 months (174 fewer/1000, 95% CI 211 fewer−126 more), less hypoglycemia <3.9 mmol/L at 3 months (436 fewer/1000, 95% CI 584 fewer−148 fewer), less hypoglycemia <3.9 mmol/L at 6 months (480 fewer/1000, 95% CI 613 fewer−200 fewer), less nocturnal hypoglycemia <3.0 mmol/L at 6 months (155 fewer/1000, 95% CI 172 fewer−165 more), less nocturnal hypoglycemia <3.9 mmol/L at 3 months (413 fewer/1000, 95% CI 100 fewer−∞), and less nocturnal hypoglycemia <3.9 mmol/L at 6 months (348 fewer/1000, 95% CI 383 fewer−74 fewer). None of the observed summary effects favored NPH.

#### 4.1.5 Glargine U‐100 versus Detemir

Three RCTs with 1077 patients reported different outcomes.^30^, ^38^, ^43^ One RCT with 42 patients compared glargine U‐100 with detemir but did not report administration time.^40^ Evidence from the following outcomes favored glargine U‐100, including HbA1c < 7.0% at 6 months (142 more/1000, 95% CI 38 more−268 more), HbA1c change at 12 months (MD of 0.42% lower, 95% CI 1.11 lower−0.27 higher), and less nocturnal hypoglycemia <3.0 mmol/L at 6 months (45 fewer/1000, 95% CI 80 fewer−17 more). The evidence favored detemir for hypoglycemia <3.0 mmol/L at 6 months (79 more patients in glargine U‐100/1000, 95% CI 5 fewer−189 more) and hypoglycemia <3.0 mmol/L at 12 months (56 more patients in glargine U‐100/1000, 95% CI 23 fewer−148 more).

#### 4.1.6 Glargine U‐100 versus NPH

Twelve RCTs with 4233 patients were included under this comparison.^14^, ^19^, ^23^, ^24^, ^26^, ^27^, ^29^, ^31^, ^36^, ^39^, ^41^, ^42^ Evidence from the following outcomes favored glargine U‐100, including HbA1c < 7.0% at 6 months (31 more/1000, 95% CI 4 fewer−63 more), less hypoglycemia <3.0 mmol/L at 3 months (52 fewer/1000, 95% CI 250 fewer−405 more), less nocturnal hypoglycemia <3.9 mmol/L at 6 months (143 fewer/1000, 95% CI 180 fewer−92 fewer), and less nocturnal hypoglycemia <3.0 mmol/L at 12 months (139 fewer/1000, 95% CI 168 fewer−101 fewer). NPH appears superior to glargine U‐100 on hypoglycemia <3.0 mmol/L at 6 months (32 more patients in glargine U‐100/1000, 95% CI 38 fewer−144 more).

#### 4.1.7 Detemir versus NPH

One RCT with 333 patients met the inclusion criteria for this comparison.^28^ Detemir appears to be more beneficial than NPH on the following outcomes: hypoglycemia <3.0 mmol/L at 6 months (165 fewer/1000, 95% CI 217 fewer−81 fewer) and nocturnal hypoglycemia <3.0 mmol/L at 6 months (87 fewer/1000, 95% CI 113 fewer−31 fewer). NPH, however, produced more desirable HbA1c change (%) at 6 months (MD of 0.26% higher) but did not reach the small clinical threshold (MD of 0.40% in Appendix Table S4).

### 4.2 One basal insulin injected at morning time versus another basal insulin injected at morning time

#### 4.2.1 Degludec U‐100 versus glargine U‐100

One RCT with 44 patients provided data for this comparison. Degludec U‐100 injected in the morning led to more optimal for FPG change at 6 months (MD of 1.97 mmol/L lower, 95% CI 2.66 lower−1.28 lower),^13^ whereas glargine U‐100 produced better HbA1c change at 6 months (MD of 0.2% higher, 95% CI 0.35 lower−0.75 higher).

#### 4.2.2 Detemir versus NPH

One RCT with 86 patients met inclusion criteria.^33^ Patients receiving detemir had less overall hypoglycemic episodes (rate/week) at 7 months: 0.079 ± 0.359 versus 0.146 ± 0.743, fewer minor hypoglycemic episodes: 0 versus 0.125 ± 0.733. None of the observed summary effects favored NPH.

### 4.3 One insulin injected in the morning versus another insulin injected at bedtime

#### 4.3.1 Detemir injected in the morning versus NPH injected at bedtime

One RCT with 329 patients met the inclusion criteria.^28^ Detemir group appears superior to NPH group on the following outcomes: hypoglycemia <3.0 mmol/L at 6 months (129 fewer/1000, 95% CI 191 fewer−39 fewer) and less nocturnal hypoglycemia <3.0 mmol/L at 6 months (110 fewer/1000, 95% CI 126 fewer−66 fewer). The evidence favored NPH for HbA1c change at 6 months (MD of 0.16% higher, 95% CI 0.07 lower−0.39 higher).

#### 4.3.2 Glargine U‐100 injected in the morning versus NPH injected at bedtime

Two RCTs with 526 patients met the inclusion criteria.^26^, ^42^ Evidence from the following outcomes favored glargine U‐100: HbA1c < 7.0% at 6 months (105 more/1000, 95% CI 22 more−213 more), HbA1c change at 6 months (MD of 0.47% lower, 95% CI 1.18 lower−0.23 higher), less severe hypoglycemia at 6 months (4 fewer/1000, 95% CI 17 fewer−38 more), and less nocturnal hypoglycemia <3.9 mmol/L at 6 months (219 fewer/1000, 95% CI 265 fewer−153 fewer). NPH at bedtime appears superior to glargine U‐100 on weight change at 6 months (MD of 1.93 kg higher, 95% CI 0.28 lower−4.14 higher), and weight end point at 6 months (MD of 3.4 kg higher, 95% CI 5.44 lower−12.2 higher).

### 4.4 One insulin injected in the morning versus itself injected at bedtime

#### 4.4.1 Detemir

One RCT with 334 patients met the inclusion criteria.^28^ Detemir morning injection appears better than bedtime injection on nocturnal hypoglycemia <3.0 mmol/L at 6 months (23 fewer/1000, 95% CI 40 fewer−32 more), whereas detemir bedtime injection was better than morning injection on less hypoglycemia <3.0 mmol/L at 6 months (34 more/1000, 95% CI 38 fewer−149 more).

#### 4.4.2 Glargine U‐100

Two RCTs with 521 patients met the inclusion criteria.^26^, ^42^ Glargine U‐100 morning injection appears more beneficial than bedtime injection on HbA1c < 7.0% at 6 months (96 more/1000, 95% CI 13 more−201 more), HbA1c change at 6 months (MD of 0.54% lower, 95% CI 1.16 lower−0.09 higher), and less nocturnal hypoglycemia <3.9 mmol/L at 6 months (64 fewer/1000, 95% CI 115 fewer−11 more). However, the glargine U‐100 bedtime injection group had less severe hypoglycemia at 6 months than morning injection group (3 more/1000, 95% CI 10 fewer−53 more).

The results of direct head‐to‐head comparison among five basal insulins are summarized in Table 4.

**TABLE 4:** Results of direct head‐to‐head comparison among five basal insulins.

Direct head‐to‐head comparison	May favor basal insulin (after considering benefits and harms from key evidence for the results with the point estimate of the critical outcome at least over the small effect thresholds)
One insulin bedtime vs. another insulin bedtime injection	
(1) Glargine U‐300 vs. degludec U‐100	Either way
(2) Degludec U‐100 vs. glargine U‐100	Degludec U‐100
(3) Glargine U‐300 vs. glargine U‐100	Glargine U‐300
(4) Glargine U‐300 vs. NPH	Glargine U‐300
(5) Glargine U‐100 vs. detemir	Either way
(6) Glargine U‐100 vs. NPH	Glargine U‐100
(7) Detemir vs NPH	Detemir
One insulin morning vs. another insulin morning injection	
(3) Degludec U‐100 morning time vs. glargine U‐100 morning time	No conclusion (sample size too small^a^)
(4) Detemir morning time vs. NPH morning time	No conclusion (sample size too small^a^)
One insulin morning vs. another insulin bedtime injection	
(3) Detemir morning time vs. NPH bedtime	Detemir morning time injection
(4) Glargine U‐100 morning time vs. NPH bedtime	Glargine U‐100 morning time injection
One insulin morning time vs. its bedtime injection	
(3) Detemir morning time vs. detemir bedtime	Either way
(4) Glargine U‐100 morning time vs. glargine U‐100 bedtime	Glargine U‐100 morning time injection

Abbreviation: NPH, insulin neutral protamine hagedorn.

^a^ We defined the small sample size as <100.

### 4.5 Patient‐reported outcomes

Ten RCTs reported patient‐reported outcomes (Supplementary Table S5).^13^, ^20^, ^22^, ^27^, ^31^, ^35^, ^37^, ^41^, ^42^, ^43^ Overall, these patient‐reported outcomes exhibited a consistent trend in direction of benefit as those of critical outcomes.

### 4.6 Network meta‐analyses

We performed network meta‐analyses for the critical outcomes. The statistically significant results are shown in Table 5. Six comparisons showed statistically significant results (ie, *p* value ≤.05) that are consistent with the results from the direct paired comparison in Supplementary Table S3.

**TABLE 5:** Statistically significant results in the network meta‐analysis.

(1) HbA1c < 7.0% at 6 months					
DegludecBed	0.6 (0.4, 1.0)	1.1 (0.9, 1.4)	1.6 (1.0, 2.6)	1.1 (0.9, 1.5)	1.0 (0.7, 1.4)
1.6 (1.0, 2.8)	DetemirBed	1.8 (1.1, 2.8)	2.7 (1.5, 5.0)	1.8 (1.1, 3.1)	1.6 (1.0, 2.7)
0.9 (0.7, 1.2)	0.6 (0.4, 0.9)	GlargineU100Bed	1.5 (1.0, 2.3)	1.0 (0.8, 1.3)	0.9 (0.7, 1.1)
0.6 (0.4, 1.0)	0.4 (0.2, 0.7)	0.7 (0.4, 1.0)	GlargineU100Mor	0.7 (0.4, 1.1)	0.6 (0.4, 0.9)
0.9 (0.7, 1.2)	0.5 (0.3, 0.9)	1.0 (0.8, 1.2)	1.5 (0.9, 2.3)	GlargineU300Bed	0.9 (0.7, 1.2)
1.0 (0.7, 1.4)	0.6 (0.4, 1.0)	1.1 (0.9, 1.4)	1.7 (1.1, 2.5)	1.2 (0.9, 1.5)	NPHBed

(2) Nocturnal hypoglycemia (defined as blood glycemia <3.9 mmol/L) at 12 months				
DegludecBed	1.5 (0.4, 7.5)	1.0 (0.2, 8.6)	1.0 (0.3, 3.3)	3.4 (1.0, 25.3)
0.7 (0.1, 2.3)	GlargineU100Bed	0.7 (0.2, 2.4)	0.66 (0.2, 1.2)	2.29 (1.2, 6.3)
1.0 (0.1, 4.4)	1.4 (0.4, 4.2)	GlargineU100Mor	0.95 (0.2, 2.8)	3.3 (1.2, 11.7)
1.0 (0.3, 3.4)	1.5 (0.8, 4.2)	1.1 (0.4, 5.3)	GlargineU300Bed	3.47 (1.7, 14.4)
0.3 (0.0, 1.0)	0.4 (0.2, 0.8)	0.3 (0.1, 0.9)	0.3 (0.1, 0.6)	NPHBed

Abbreviations: Bed, injection at bedtime; HbA1c, glycated hemoglobin; Mor, injection at morning time.

## 5 Q2. INITIAL OPTIMAL DOSE (U/KG/DAY) FOR INITIATION OF THE FIVE BASAL INSULIN

### 5.1 Glargine U‐300, degludec U‐100, and NPH

No eligible RCT compared the different initial starting doses for glargine U‐300, degludec U‐100, and NPH. Among the 35 included RCTs, 8 indicated that the initial dose for glargine U‐300 was 0.20 U/kg/day,^11^, ^15^, ^16^, ^17^, ^18^, ^20^, ^21^, ^35^ from 0.10 U/kg/day to 0.20 U/kg/day for degludec U‐100,^13^, ^15^, ^16^, ^18^, ^21^, ^22^, ^34^, ^37^ from 0.11 U/kg/day to 0.23 U/kg/day for NPH,^11^, ^14^, ^26^, ^31^, ^33^, ^39^, ^41^, ^42^ respectively in Supplementary Table S6.

### 5.2 Detemir

One RCT with 60 patients reported that, compared with twice daily, injecting detemir 0.12 U/kg/day once daily led to a 0.3% lower MD (95% CI 1.26 lower−0.66 higher) on change of HbA1c at 3 months, but more patients reached HbA1c < 7% at 3 months in the twice‐daily group (81 fewer, 95% CI 207 fewer−266 more).^10^ Six of 35 RCTs indicated that the range of the initial dose for detemir was from 0.10 U/kg/day to 0.20 U/kg/day in Supplementary Table S6.^30^, ^32^, ^33^, ^38^, ^40^, ^43^

### 5.3 Glargine U‐100

One RCT with 892 patients reported that an initial dose of 0.2 U/kg/day for glargine U‐100 resulted in fewer patients with hypoglycemia <3.9 mmol/L at 4 months and nocturnal hypoglycemia <3.9% at 3 months than glargine U‐100 0.3 U/kg/day, and two different initial doses had trivial or no differences for the percentage of patients reaching HbA1c < 7% at 4 months and the HbA1c change at 3 months.^12^ Among the 35 included RCTs, the range of the initial dose for glargine U‐100 was from 0.10 U/kg/day to 0.20 U/kg/day across 17 RCTs (Supplementary Table S6).^9^, ^10^, ^13^, ^14^, ^20^, ^22^, ^25^, ^30^, ^31^, ^34^, ^35^, ^37^, ^38^, ^39^, ^41^, ^42^, ^43^

## 6 Q3. OPTIMAL DOSE RANGE (U/KG/DAY) OF THE BASAL INSULIN POST INITIATION

There is no eligible RCT investigating the optimal end point dose for any basal insulin to maintain satisfactory control of FPG. Among the 35 included RCTs, 7 RCTs indicated that the range of the end point dose for glargine U‐300 was between 0.34 and 0.62 U/kg/day,^15^, ^16^, ^17^, ^18^, ^20^, ^21^, ^35^ and seven RCTs indicated the range of the end point dose for degludec U‐100 was between 0.28 and 0.59 U/kg/day.^15^, ^16^, ^18^, ^21^, ^22^, ^34^, ^37^ Seven RCTs indicated that the range of the end point dose for detemir was between 0.19 and 0.78 U/kg/day.^10^, ^28^, ^30^, ^32^, ^38^, ^40^, ^43^ Because detemir was allowed to be injected twice per day when needed, we assumed that twice‐daily injection was adopted when the end point dose was≥0.6 U/kg/day. There were 20 RCTs that indicated the range of end point dose for glargine U‐100 was between 0.34 and 0.62 U/kg/day^9^, ^12^, ^17^, ^20^, ^22^, ^23^, ^24^, ^25^, ^26^, ^30^, ^31^, ^34^, ^35^, ^37^, ^38^, ^39^, ^40^, ^41^, ^42^, ^43^ and eight RCTs indicated the range of the end point dose for NPH was from 0.19 to 0.66 U/kg/day in Supplementary Table S7.^23^, ^24^, ^26^, ^28^, ^31^, ^39^, ^41^, ^42^

## 7 Q4. RANGE OF TARGET FPG CAN LEAD TO THE IDEAL HBA1C LEVEL

Three RCTs met inclusion criteria.^9^, ^25^, ^32^ One RCT with 244 patients treated with detemir reported that when compared with the range of 4.4 < FPG≤6.1 mmol/L, the range of 3.9 < FPG≤5.0 mmol/L led to more hypoglycemia (111 more/1000, 95% CI 12 fewer–275 more) and nocturnal hypoglycemia (100 more/1000, 95% CI 8 fewer–270 more) at 6 months but led to more patients reaching HbA1c < 7% (103 more/1000, 95% CI 16 fewer–254 more).^15^

The other two RCTs with 1018 patients focused on glargine U‐100.^9^, ^25^ Compared with the range of 3.9 < FPG≤6.1 mmol/L, the range of 3.9 < FPG≤5.6 mmol/L led to more hypoglycemia (115 more/1000, 95% CI 22 more–236 more) and nocturnal hypoglycemia (92 more/1000, 95% CI 19 more–205 more) and resulted in fewer patients reaching HbA1c < 7% (AE 59 fewer/1000, 95% CI 244 fewer–293 more) at 6 months. Compared with the range of 3.9 < FPG≤7.0 mmol/L, the range of 3.9 < FPG≤6.1 mmol/L led to more hypoglycemia at≤3.9 mmol/L (42 more/1000, 95% CI 16 fewer–116 more) and nocturnal hypoglycemia at ≤3.9 mmol/L (21 more/1000, 95% CI 16 fewer–78 more) but resulted in more patients reaching HbA1c < 7% (87 more/1000, 95% CI 18 more–162 more) and fewer patients having hypoglycemia at <3.0 (17 fewer/1000, 95% CI 29 fewer–9 more) mmol/L at 6 months.

## 8 DISCUSSION

As demonstrated by the synthesized results of a direct comparison between the five basal insulins in Table 4 and the network meta‐analyses in Table 5, we found that when injecting basal insulin at bedtime, the optimal choice in descending order might be glargine U‐300 or degludec U‐100, glargine U‐100 or detemir, and lastly NPH. Injecting glargine U‐100 in the morning may be more effective and lead to fewer hypoglycemic events than injecting it at bedtime. However, future high‐quality research is needed to confirm these because of the low quality of the evidence.

The current evidence shows the starting dose for initiation of any of the five basal insulins to be from 0.10 U/kg/day to 0.20 U/kg/day according to the individual patient's characteristics, such as age, weight, morbidities, kidney function, etc. It appears that an FPG range of 3.9–6.1 mmol/L for any basal insulin may be an acceptable range for achieving a target HbA1c level of <7%. However, for individuals at high risk of hypoglycemia, such as with serious diseases that affect life, severe hypoglycemia history, acute cerebrovascular disease, or severe chronic renal failure leading increased risk of hypoglycemia, the target range of FPG should be higher.

This study is the first systematic review that defined thresholds for outcomes that correspond to trivial/none, small, moderate, or large effects and used them to rate the imprecision domain when assessing quality of evidence and balancing the magnitudes of the desirable and undesirable outcomes' effects according to the GRADE approach in the diabetes community. Thus, this paper provides the references for future researchers to set up clinical thresholds for these outcomes in their own research according to their individual experiences and different contexts.

It should be noted that statistically significant and clinically significant findings are different.^45^ For example, when degludec U‐100 was compared with glargine U‐100, there were 41 fewer patients/1000 who had hypoglycemic events (<3.0 mmol/L) at 6 months (95% CI, 89 fewer−11 more) in the degludec U‐100 group, which showed there was no statistical significance. However, the point estimate of 41 patients is higher than our clinical threshold of the small effect (20 patients/1000 in Supplementary Table S4). The upper boundary of 95% CI was 11 patients, which did not reach the small clinical threshold (20 patients/1000), and the lower boundary of 95% CI was higher than our clinical threshold of a large effect (>80 patients/1000) to favor degludec U‐100. Hence, we can consider that the result may be clinically significant favoring degludec U‐100. Setting up clinical thresholds instead of only considering statistical significance is very important in conducting systematic reviews, as it helps clinicians make appropriate clinical decisions. This point is emphasized in the 2022 version of the *Cochrane Handbook* for Systematic Reviews of Interventions.^46^

This systematic review has some limitations. First, the literature search was restricted to English language publications, which can potentially lead to missing references published in other languages. Second, we included only RCTs. There might be moderate‐quality nonrandomized studies beyond our search that could potentially answer some of our research questions.

Among the included studies, there is no subgroup analysis by ethnicity, which should be considered by investigators in their future research. Finally, individualized patient care is the key in clinical practice, and so, treatment plans should always be discussed in consideration of the individual patient's values and preferences.

## AUTHOR CONTRIBUTIONS

Linong Ji, Yingying Luo, Jun Xia, and Xiaomei Yao conceived and designed this study. Zhan Zhao and Chenchen Xu conducted the database search and reviewed the reference lists of articles included in screening. Zhan Zhao and Chenchen Xuperformed initial screening and review of full texts for eligibility. Zhan Zhao and Chenchen Xu extracted the data and completed quality assessment. Xiaomei Yao resolved any conflicts in quality assessment. Yaping Chang and Zhan Zhao prepared the tables and figures, and conducted the data analysis. Yingying Luo, Jun Xia, Xiaomei Yao, and Linong Ji conducted data interpretation and drafted the first draft of the manuscript. All authors approved the project plan, and reviewed and revised the final manuscript before submission.

## FUNDING INFORMATION

Sponsored by the Chinese Geriatric Endocrine Society. The Chinese Geriatric Endocrine Society is a not‐for‐profit organization that accepts donations and support from the community and industry. All work done by the authors is editorially independent of the Chinese Geriatric Endocrine Society.

## CONFLICT OF INTEREST STATEMENT

Within the past 4 years, Yong Mong Bee and Daisuke Yabe have received consulting remuneration from a commercial entity or other organization with an interest related to the subjectiveness of the meeting or work; Siew Pheng Chan, Margaret McGill, Daisuke Yabe, or their research teams have received support from a commercial entity or other organization with an interest related to the subjectiveness of the meeting or work respectively; Siew Pheng Chan also has received nonmonetary support valued at more than US $1000 (including equipment, facilities, research assistants, paid travel meetings, etc.). Ketut Suastika received honoraria for a scientific symposium or webinar on basal insulin from several pharmaceutical companies, and almost all the events were in collaboration with Indonesia Society of Endocrinology. Khue Nguyen Thy received an honorarium for the chair in scientific meetings from Servier, Boehringer Ingelheim and Eisai. Soo Lim received research funding from MSD and CKD, and honoraria for lectures from Novo Nordisk, Sanofi, Boehringer Ingelheim, AstraZeneca, and MSD. Linong Ji has received consulting and lecture fees from Eli Lilly, Novo Nordisk, Merck, Bayer, Sanofi‐Aventis, Roche, MSD, Metronics AstraZeneca, Boehinger Ingelheim, and Abbott. Other authors declare no competing interest.

## Supporting information


**Table S1.** Search strategy.
**Table S2.** Risk of bias assessments for individual studies.
**Table S3.** Grading of Recommendations, Assessment, Development, and Evaluations (GRADE) summary of findings tables.
**Table S4.** The clinical thresholds of trial, small, moderate, and large effects for relevant outcomes.
**Table S5.** Patient‐reported outcomes.
**Table S6.** The summarized initial dose for the five types of basal insulins from 35 included studies.
**Table S7.** The summarized end point insulin dose for the five types of basal insulins from 35 included studies.

## Data Availability

Most of the systematic review data are available in the supplementary materials. Additional requests can be provided by contacting authors.
