# Supplementary material for: Effectiveness, safety, initial optimal dose, and optimal maintenance dose range of basal insulin regimens for type 2 diabetes: A systematic review with meta‐analysis
Source: J Diabetes. 2023 Apr 10;15(5):419–35. doi: 10.1111/1753-0407.13381 (PMC10172019; doi:10.1111/1753-0407.13381)
Supplement: Supplementary file 1 — Table S1. Search strategy. Table S2. Risk of bias assessments for individual studies. Table S3. Grading of Recommendations, Assessment, Development, and Evaluations (GRADE) summary of findings tables. Table S4. The clinical thresholds of trial, small, moderate, and large effects for relevant outcomes. Table S5. Patient‐reported outcomes. Table S6. The summarized initial dose for the five types of basal insulins from 35 included studies. Table S7. The summarized end point insulin dose for the five types of basal insulins from 35 included studies. [file JDB-15-419-s001.doc]

# Appendix Table S1. Search strategy (performed on Feb 17, 2022)

MEDLINE (PubMed)

(("Diabetes Mellitus, Type 2"[Mesh] OR "type 2 diabetes"[tw] OR "type ii diabetes"[tw] OR "type two diabetes"[tw] OR "type 2 diabet*"[tw] OR "type ii diabet*"[tw] OR "type two diabet*"[tw] OR "T2DM"[tw] OR "Adult Onset Diabet*"[tw] OR "Adult Onset Diabetes"[tw] OR "NIDDM"[tw] OR "Ketosis Resistant Diabet*"[tw] OR "Ketosis Resistant Diabetes"[tw] OR "Non Insulin Dependent Diabet*"[tw] OR "Non Insulin Dependent Diabetes"[tw] OR "Noninsulin Dependent Diabet*"[tw] OR "Noninsulin Dependent Diabetes"[tw] OR "Slow Onset Diabet*"[tw] OR "Slow Onset Diabetes"[tw] OR "Stable Diabet*"[tw] OR "Stable Diabetes"[tw]) AND ("insulin degludec"[Supplementary Concept] OR "insulin degludec"[tw] OR "Degludec"[tw] OR "Insulin Glargine"[Mesh] OR "Insulin Glargine"[tw] OR "Insulin Glargine U-300"[tw] OR "Insulin glargine U-100"[tw] OR "Glargine"[tw] OR "Lantus"[tw] OR "Basaglar"[tw] OR "HOE 901"[tw] OR "Insulin Detemir"[Mesh] OR "Insulin Detemir"[tw] OR "Detemir"[tw] OR "Levemir"[tw] OR "NN 304"[tw] OR "Insulin, Isophane"[Mesh] OR "Hagedorn"[tw] OR "Isophane Insulin"[tw] OR "Neutral Protamine Hagedorn Insulin"[tw] OR "NPH Insulin"[tw] OR "NPH"[tw] OR "Protamine Hagedorn Insulin"[tw] OR "Protamine Zinc Insulin"[tw])) NOT ("comment"[pt] OR "letter"[pt] OR "editorial"[pt] OR "note"[ti] OR "Published Erratum"[pt] OR "short survey"[ti] OR "News"[pt] OR "newspaper article"[pt] OR "Patient Education Handout"[pt] OR "case reports"[pt] OR "historical article"[pt] OR "case study"[ti] OR "comment"[ti] OR "letter"[ti] OR "editorial"[ti] OR "Erratum"[ti] OR "corrigendum"[ti] OR "Patient Education Handout"[ti] OR "case report"[ti] OR "historical article"[ti]) NOT ("animals"[mesh] NOT "Humans"[mesh]) AND (english[la])

Embase (OVID)

((exp *"non insulin dependent diabetes mellitus"/ OR "type 2 diabetes".ti,ab OR "type ii diabetes".ti,ab OR "type two diabetes".ti,ab OR "type 2 diabet*".ti,ab OR "type ii diabet*".ti,ab OR "type two diabet*".ti,ab OR "T2DM".ti,ab OR "Adult Onset Diabet*".ti,ab OR "Adult Onset Diabetes".ti,ab OR "NIDDM".ti,ab OR "Ketosis Resistant Diabet*".ti,ab OR "Ketosis Resistant Diabetes".ti,ab OR "Non Insulin Dependent Diabet*".ti,ab OR "Non Insulin Dependent Diabetes".ti,ab OR "Noninsulin Dependent Diabet*".ti,ab OR "Noninsulin Dependent Diabetes".ti,ab OR "Slow Onset Diabet*".ti,ab OR "Slow Onset Diabetes".ti,ab OR "Stable Diabet*".ti,ab OR "Stable Diabetes".ti,ab) AND (exp *"insulin degludec"/ OR "insulin degludec".ti,ab OR "Degludec".ti,ab OR exp *"Insulin Glargine"/ OR "Insulin Glargine".ti,ab OR "Insulin Glargine U-300".ti,ab OR "Insulin glargine U-100".ti,ab OR "Glargine".ti,ab OR "Lantus".ti,ab OR "Basaglar".ti,ab OR "HOE 901".ti,ab OR exp *"Insulin Detemir"/ OR "Insulin Detemir".ti,ab OR "Detemir".ti,ab OR "Levemir".ti,ab OR "NN 304".ti,ab OR exp *"Isophane Insulin"/ OR "Hagedorn".ti,ab OR "Isophane Insulin".ti,ab OR "Neutral Protamine Hagedorn Insulin".ti,ab OR "NPH Insulin".ti,ab OR "NPH".ti,ab OR "Protamine Hagedorn Insulin".ti,ab OR "Protamine Zinc Insulin".ti,ab)) NOT (exp "letter"/ OR exp "editorial"/ OR "note"/ OR "note".ti OR exp "Erratum"/ OR exp "short survey"/ OR "short survey".ti OR "News".ti OR "newspaper article".ti OR exp "case report"/ OR exp "case study"/ OR "case study".ti OR "comment".ti OR "letter".ti OR "editorial".ti OR "Erratum".ti OR "corrigendum".ti OR "Patient Education Handout".ti OR "case report".ti OR "historical article".ti) NOT (exp "animals"/ NOT exp "Humans"/) AND (english.la)

Web of Science

TS=(("non insulin dependent diabetes mellitus" OR "type 2 diabetes" OR "type ii diabetes" OR "type two diabetes" OR "type 2 diabet*" OR "type ii diabet*" OR "type two diabet*" OR "T2DM" OR "Adult Onset Diabet*" OR "Adult Onset Diabetes" OR "NIDDM" OR "Ketosis Resistant Diabet*" OR "Ketosis Resistant Diabetes" OR "Non Insulin Dependent Diabet*" OR "Non Insulin Dependent Diabetes" OR "Noninsulin Dependent Diabet*" OR "Noninsulin Dependent Diabetes" OR "Slow Onset Diabet*" OR "Slow Onset Diabetes" OR "Stable Diabet*" OR "Stable Diabetes") AND ("insulin degludec" OR "insulin degludec" OR "Degludec" OR "Insulin Glargine" OR "Insulin Glargine" OR "Insulin Glargine U-300" OR "Insulin glargine U-100" OR "Glargine" OR "Lantus" OR "Basaglar" OR "HOE 901" OR "Insulin Detemir" OR "Insulin Detemir" OR "Detemir" OR "Levemir" OR "NN 304" OR "Isophane Insulin" OR "Hagedorn" OR "Isophane Insulin" OR "Neutral Protamine Hagedorn Insulin" OR "NPH Insulin" OR "NPH" OR "Protamine Hagedorn Insulin" OR "Protamine Zinc Insulin")) NOT TI=("comment" OR "letter" OR "editorial" OR "note" OR "Published Erratum" OR "short survey" OR "News" OR "newspaper article" OR "Patient Education Handout" OR "case reports" OR "historical article" OR "case study" OR "comment" OR "letter" OR "editorial" OR "Erratum" OR "corrigendum" OR "Patient Education Handout" OR "case report" OR "historical article") NOT ti=("veterinary" OR "rabbit" OR "rabbits" OR "animal" OR "animals" OR "mouse" OR "mice" OR "rodent" OR "rodents" OR "rat" OR "rats" OR "pig" OR "pigs" OR "porcine" OR "horse" OR "horses" OR "equine" OR "cow" OR "cows" OR "bovine" OR "goat" OR "goats" OR "sheep" OR "ovine" OR "canine" OR "dog" OR "dogs" OR "feline" OR "cat" OR "cats") AND la=(english)

Cochrane Library

(("non insulin dependent diabetes mellitus" OR "type 2 diabetes" OR "type ii diabetes" OR "type two diabetes" OR "type 2 diabet*" OR "type ii diabet*" OR "type two diabet*" OR "T2DM" OR "Adult Onset Diabet*" OR "Adult Onset Diabetes" OR "NIDDM" OR "Ketosis Resistant Diabet*" OR "Ketosis Resistant Diabetes" OR "Non Insulin Dependent Diabet*" OR "Non Insulin Dependent Diabetes" OR "Noninsulin Dependent Diabet*" OR "Noninsulin Dependent Diabetes" OR "Slow Onset Diabet*" OR "Slow Onset Diabetes" OR "Stable Diabet*" OR "Stable Diabetes") AND ("insulin degludec" OR "insulin degludec" OR "Degludec" OR "Insulin Glargine" OR "Insulin Glargine" OR "Insulin Glargine U-300" OR "Insulin glargine U-100" OR "Glargine" OR "Lantus" OR "Basaglar" OR "HOE 901" OR "Insulin Detemir" OR "Insulin Detemir" OR "Detemir" OR "Levemir" OR "NN 304" OR "Isophane Insulin" OR "Hagedorn" OR "Isophane Insulin" OR "Neutral Protamine Hagedorn Insulin" OR "NPH Insulin" OR "NPH" OR "Protamine Hagedorn Insulin" OR "Protamine Zinc Insulin")):ti,ab,kw

MEDLINE (PubMed):

Guidelines

("Guidelines as Topic"[Mesh] OR "Guideline"[Publication Type] OR "Guidelines"[tw] OR "Guideline"[tw]) AND ("2000/01/01"[PDAT] : "3000/12/31"[PDAT])

Systematic Reviews

("systematic"[sb]) AND ("2016/01/01"[PDAT] : "3000/12/31"[PDAT])

RCTs

(randomized controlled trial[pt] OR controlled clinical trial[pt] OR randomized[tiab] OR placebo[tiab] OR drug therapy[sh] OR randomly[tiab] OR trial[tiab] OR groups[tiab] NOT (animals [mh] NOT humans [mh])) AND ("2000/01/01"[PDAT] : "3000/12/31"[PDAT])

Observational Studies

("Observational Study"[Publication Type] OR "Observational Studies as Topic"[Mesh] OR "Cohort Studies"[Mesh] OR "Case-Control Studies"[Mesh] OR "Cross-Sectional Studies"[Mesh] OR Observational Stud*[tiab] OR Cohort[tiab] OR "Follow-Up"[tiab] OR Longitudinal*[tiab] OR Prospectiv*[tiab] OR Retrospectiv*[tiab] OR "Case-Control"[tiab] OR "Cross-Sectional"[tiab] OR "case series"[tiab] OR "single arm"[tiab]) NOT ("Animals"[Mesh] NOT ("Humans"[Mesh] AND "Animals"[Mesh])) AND ("2000/01/01"[PDAT] : "3000/12/31"[PDAT])

Embase (OVID):

Guidelines

(exp "Practice Guideline"/ OR "Guidelines".mp OR "Guideline".mp) AND 2000:2023.(sa_year)

Systematic Reviews

("systematic review"/ OR "systematic review".ti,ab) AND 2016:2023.(sa_year)

RCTs

(exp "randomized controlled trial"/ OR exp "controlled clinical trial"/ OR randomized.ti,ab OR placebo.ti,ab OR randomly.ti,ab OR trial.ti,ab OR groups.ti,ab NOT (exp "animals"/ NOT exp "humans"/)) AND 2000:2023.(sa_year)

Observational Studies

(exp "Observational Study"/ OR exp "Cohort Analysis"/ OR exp "Case Control Study"/ OR exp "Cross-Sectional Study"/ OR Observational Stud*.mp OR Cohort.mp OR "Follow-Up".mp OR Longitudinal*.mp OR Prospectiv*.mp OR Retrospectiv*.mp OR "Case-Control".mp OR "Cross-Sectional".mp OR "case series".mp OR "single arm".mp) NOT (exp "Animals"/ NOT (exp "Humans"/ AND exp "Animals"/)) AND 2000:2023.(sa_year)

Web of Science:

Guidelines

TS=("Practice Guideline" OR "Guidelines" OR "Guideline") AND PY=(2000 OR 2001 OR 2002 OR 2003 OR 2004 OR 2005 OR 2006 OR 2007 OR 2008 OR 2009 OR 2010 OR 2011 OR 2012 OR 2013 OR 2014 OR 2015 OR 2016 OR 2017 OR 2018 OR 2019 OR 2020 OR 2021 or 2022 OR 2023)

Systematic Reviews

TS=("systematic review") AND PY=(2016 OR 2017 OR 2018 OR 2019 OR 2020 OR 2021 or 2022 OR 2023)

RCTs

TS=("randomized controlled trial" OR "controlled clinical trial" OR randomized OR placebo OR drug therapy OR randomly OR trial OR groups NOT ("animals" NOT "humans")) AND PY=(2000 OR 2001 OR 2002 OR 2003 OR 2004 OR 2005 OR 2006 OR 2007 OR 2008 OR 2009 OR 2010 OR 2011 OR 2012 OR 2013 OR 2014 OR 2015 OR 2016 OR 2017 OR 2018 OR 2019 OR 2020 OR 2021 or 2022 OR 2023)

Observational Studies

TS=("Observational Study" OR "Cohort Analysis" OR "Case Control Study" OR "Cross-Sectional Study" OR Observational Stud* OR Cohort OR "Follow-Up" OR Longitudinal* OR Prospectiv* OR Retrospectiv* OR "Case-Control" OR "Cross-Sectional" OR "case series" OR "single arm") AND PY=(2000 OR 2001 OR 2002 OR 2003 OR 2004 OR 2005 OR 2006 OR 2007 OR 2008 OR 2009 OR 2010 OR 2011 OR 2012 OR 2013 OR 2014 OR 2015 OR 2016 OR 2017 OR 2018 OR 2019 OR 2020 OR 2021 or 2022 OR 2023)

# Appendix Table S2. Risk of bias assessments for individual studies

| **Study** | | **Domain 1: Randomization Process** | **Domain 2: Deviation from Intervention** | **Domain 3:**  **Missing Outcome Data** | **Domain 4:**  **Measurement of Outcome** | **Domain 5: Reported Results** | **Overall** |
| --- | --- | --- | --- | --- | --- | --- | --- |
|  |  |  |  |  |  |  |  |
| **Question 1. What are the differences in the effectiveness and safety among five basal insulin regimens after the initiation of insulin therapy in adult patients with T2DM?** | | | | | | | |
| Ling 2021 | HbA1c control | Low risk | Some concerns | Low risk | Low risk | Some concerns | Some concerns |
|  | Incidence of hypoglycemia | Low risk | Some concerns | Low risk | Low risk | Some concerns | Some concerns |
|  | Body weight change | Low risk | Some concerns | Low risk | Low risk | Some concerns | Some concerns |
|  | Time in range | Low risk | Some concerns | Low risk | Low risk | Some concerns | Some concerns |
| Bolli 2021 | Incidence of hypoglycemia | Some concerns | Some concerns | Low risk | Low risk | Low risk | Some concerns |
| Ji 2020 | HbA1c control | Some concerns | Some concerns | Low risk | Low risk | Low risk | Some concerns |
|  | Incidence of hypoglycemia | Some concerns | Some concerns | Low risk | Low risk | Low risk | Some concerns |
|  | Body weight change | Some concerns | Some concerns | Low risk | Low risk | Low risk | Some concerns |
| Cheng 2020 | HbA1c control | Some concerns | Some concerns | Low risk | Low risk | Low risk | Some concerns |
|  | Incidence of hypoglycemia | Some concerns | Some concerns | Low risk | Low risk | Low risk | Some concerns |
|  | Body weight change | Some concerns | Some concerns | Low risk | Low risk | Low risk | Some concerns |
| Haluzik 2020 | Incidence of hypoglycemia | Some concerns | Some concerns | Low risk | Low risk | Low risk | Some concerns |
| Rosenstock 2018 | HbA1c control | Some concerns | Some concerns | Low risk | Low risk | Low risk | Some concerns |
|  | Incidence of hypoglycemia | Some concerns | Some concerns | Low risk | Low risk | Low risk | Some concerns |
|  | Body weight change | Some concerns | Some concerns | Low risk | Low risk | Low risk | Some concerns |
| Aso 2017 | Incidence of hypoglycemia | Some concerns | Some concerns | Low risk | Low risk | Low risk | Some concerns |
| Bolli 2017 | HbA1c control | Some concerns | Some concerns | Low risk | Low risk | Low risk | Some concerns |
|  | Incidence of hypoglycemia | Some concerns | Some concerns | Low risk | Low risk | Low risk | Some concerns |
|  | Body weight change | Some concerns | Some concerns | Low risk | Low risk | Low risk | Some concerns |
| Pan 2016 | HbA1c control | Some concerns | Some concerns | Low risk | Low risk | Low risk | Some concerns |
|  | Incidence of hypoglycemia | Some concerns | Some concerns | Low risk | Low risk | Low risk | Some concerns |
|  | Body weight change | Some concerns | Some concerns | Low risk | Low risk | Low risk | Some concerns |
| Bolli 2015 | HbA1c control | Some concerns | Some concerns | Low risk | Low risk | Low risk | Some concerns |
|  | FPG control | Some concerns | Some concerns | Low risk | Low risk | Low risk | Some concerns |
|  | Incidence of hypoglycemia | Some concerns | Some concerns | Low risk | Low risk | Low risk | Some concerns |
|  | Body weight change | Some concerns | Some concerns | Low risk | Low risk | Low risk | Some concerns |
| Elisha 2015 | Body weight change | Some concerns | Some concerns | Low risk | Low risk | Low risk | Some concerns |
| Hermanns 2015 | HbA1c control | Some concerns | Some concerns | Low risk | Low risk | Low risk | Some concerns |
|  | Incidence of hypoglycemia | Some concerns | Some concerns | Low risk | Low risk | Low risk | Some concerns |
|  | Body weight change | Some concerns | Some concerns | Low risk | Low risk | Low risk | Some concerns |
| Home 2015 | HbA1c control | Low risk | Some concerns | Low risk | Low risk | Low risk | Some concerns |
|  | FPG control | Low risk | Some concerns | Low risk | Low risk | Low risk | Some concerns |
|  | Incidence of hypoglycemia | Low risk | Some concerns | Low risk | Low risk | Low risk | Some concerns |
|  | Body weight change | Low risk | Some concerns | Low risk | Low risk | Low risk | Some concerns |
| Cander 2014 | Body weight change | Some concerns | Some concerns | Low risk | Low risk | Some concerns | Some concerns |
| Oikonomou 2014 | HbA1c control | Low risk | Low risk | Low risk | Low risk | Some concerns | Some concerns |
|  | Body weight change | Low risk | Low risk | Low risk | Low risk | Some concerns | Some concerns |
| Meneghini 2013 | HbA1c control | Some concerns | Low risk | Low risk | Low risk | Low risk | Some concerns |
|  | FPG control | Some concerns | Low risk | Low risk | Low risk | Low risk | Some concerns |
|  | Incidence of hypoglycemia | Some concerns | Low risk | Low risk | Low risk | Low risk | Some concerns |
|  | Body weight change | Some concerns | Low risk | Low risk | Low risk | Low risk | Some concerns |
| Onishi 2013 | HbA1c control | Some concerns | Some concerns | Low risk | Low risk | Low risk | Some concerns |
|  | Incidence of hypoglycemia | Some concerns | Some concerns | Low risk | Low risk | Low risk | Some concerns |
|  | Body weight change | Some concerns | Some concerns | Low risk | Low risk | Low risk | Some concerns |
| Zinman 2012 | HbA1c control | Some concerns | Some concerns | Low risk | Low risk | Low risk | Some concerns |
|  | Incidence of hypoglycemia | Some concerns | Some concerns | Low risk | Low risk | Low risk | Some concerns |
|  | Body weight change | Some concerns | Some concerns | Low risk | Low risk | Low risk | Some concerns |
| Hsia 2011 | HbA1c control | High risk | Some concerns | Low risk | Low risk | Low risk | High risk |
|  | FPG control | High risk | Some concerns | Low risk | Low risk | Low risk | High risk |
|  | Incidence of hypoglycemia | High risk | Some concerns | Low risk | Low risk | Low risk | High risk |
|  | Body weight change | High risk | Some concerns | Low risk | Low risk | Low risk | High risk |
| Forst 2010 | Incidence of hypoglycemia | Some concerns | Some concerns | Low risk | Low risk | Low risk | Some concerns |
| Rosenstock 2008 | HbA1c control | Low risk | Some concerns | Low risk | Low risk | Low risk | Some concerns |
|  | FPG control | Low risk | Some concerns | Low risk | Low risk | Low risk | Some concerns |
|  | Incidence of hypoglycemia | Low risk | Some concerns | Low risk | Low risk | Low risk | Some concerns |
|  | Body weight change | Low risk | Some concerns | Low risk | Low risk | Low risk | Some concerns |
| Pan 2007 | HbA1c control | Some concerns | Some concerns | Low risk | Low risk | Low risk | Some concerns |
|  | FPG control | Some concerns | Some concerns | Low risk | Low risk | Low risk | Some concerns |
|  | Incidence of hypoglycemia | Some concerns | Some concerns | Low risk | Low risk | Low risk | Some concerns |
| Eliaschewitz 2006 | HbA1c control | Some concerns | Some concerns | Low risk | Low risk | Some concerns | Some concerns |
|  | FPG control | Some concerns | Some concerns | Low risk | Low risk | Some concerns | Some concerns |
|  | Incidence of hypoglycemia | Some concerns | Some concerns | Low risk | Low risk | Some concerns | Some concerns |
| Yki-Jarvinen 2006 | HbA1c control | Low risk | Some concerns | Low risk | Low risk | Low risk | Some concerns |
|  | FPG control | Low risk | Some concerns | Low risk | Low risk | Low risk | Some concerns |
|  | Incidence of hypoglycemia | Low risk | Some concerns | Low risk | Low risk | Low risk | Some concerns |
|  | Body weight change | Low risk | Some concerns | Low risk | Low risk | Low risk | Some concerns |
| Tsimikas 2006 | Incidence of hypoglycemia | Low risk | Some concerns | Low risk | Low risk | Some concerns | Some concerns |
|  | Body weight change | Low risk | Some concerns | Low risk | Low risk | Some concerns | Some concerns |
| Benedetti 2003 | FPG control | Low risk | Some concerns | Low risk | Low risk | Low risk | Some concerns |
|  | Incidence of hypoglycemia | Low risk | Some concerns | Low risk | Low risk | Low risk | Some concerns |
| Fritsche 2003 | HbA1c control | Some concerns | Some concerns | Low risk | Low risk | Some concerns | Some concerns |
|  | Incidence of hypoglycemia | Some concerns | Some concerns | Low risk | Low risk | Some concerns | Some concerns |
|  | Body weight change | Some concerns | Some concerns | Low risk | Low risk | Some concerns | Some concerns |
| Riddle 2003 | HbA1c control | Some concerns | Some concerns | Low risk | Low risk | Low risk | Some concerns |
|  | FPG control | Some concerns | Some concerns | Low risk | Low risk | Low risk | Some concerns |
|  | Incidence of hypoglycemia | Some concerns | Some concerns | Low risk | Low risk | Low risk | Some concerns |
|  | Body weight change | Some concerns | Some concerns | Low risk | Low risk | Low risk | Some concerns |
| Yki-Jarvinen 2000 | Incidence of hypoglycemia | Some concerns | Some concerns | Low risk | Low risk | Some concerns | Some concerns |
|  | Body weight change | Some concerns | Some concerns | Low risk | Low risk | Some concerns | Some concerns |
| NCT00506662 | Incidence of hypoglycemia | Some concerns | Some concerns | Low risk | Low risk | Low risk | Some concerns |
| **Question 2. What is the optimal starting dose (U/kg/day) for initiation of the five basal insulins and optimal time of injection (morning versus bedtime administration) to control glycemia with acceptable side effects for the target population?** | | | | | | | |
| Ji 2020 | HbA1c control | Some concerns | Some concerns | Low risk | Low risk | Low risk | Some concerns |
|  | FPG control | Some concerns | Some concerns | Low risk | Low risk | Low risk | Some concerns |
|  | Incidence of hypoglycemia | Some concerns | Some concerns | Low risk | Low risk | Low risk | Some concerns |
|  | Body weight change | Some concerns | Some concerns | Low risk | Low risk | Low risk | Some concerns |
| Cander 2014 | HbA1c control | Some concerns | Some concerns | Low risk | Low risk | Low risk | Some concerns |
|  | Incidence of hypoglycemia | Some concerns | Some concerns | Low risk | Low risk | Low risk | Some concerns |
|  | Body weight change | Some concerns | Some concerns | Low risk | Low risk | Low risk | Some concerns |
| **Question 4. After initiation of any of the five basal insulins, what range of target FPG can lead to the ideal HbA1c level (i.e., < 7.0%) in the target population?** | | | | | | | |
| Yuan 2021 | HbA1c control | Some concerns | Some concerns | Low risk | Low risk | Low risk | Some concerns |
|  | Incidence of hypoglycemia | Some concerns | Some concerns | Low risk | Low risk | Low risk | Some concerns |
| Yang 2019 | HbA1c control | Some concerns | High risk | Low risk | Low risk | Low risk | High risk |
|  | Incidence of hypoglycemia | Some concerns | High risk | Low risk | Low risk | Low risk | High risk |
| Blonde 2009 | HbA1c control | Some concerns | Some concerns | Low risk | Low risk | Low risk | Some concerns |
|  | Incidence of hypoglycemia | Some concerns | Some concerns | Low risk | Low risk | Low risk | Some concerns |
| FPG: fasting plasma glucose; HbA1c: Glycosylated Hemoglobin, Type A1c. | | | | | | | |

# Appendix Table S3. GRADE summary of findings tables

# Q1. What are the differences in the effectiveness and safety among five basal insulin regimens after the initiation of insulin therapy in adult patients with T2DM?

## 1. One basal insulin injected at bedtime versus another basal insulin injected at bedtime

### Glargine U-300 vs. Degludec U-100

| **Certainty assessment** | | | | | | | **№ of patients** | | **Effect** | | **Certainty** |
| --- | --- | --- | --- | --- | --- | --- | --- | --- | --- | --- | --- |
| **№ of studies** | **Study design** | **Risk of bias** | **Inconsistency** | **Indirectness** | **Imprecision** | **Other considerations** | **Glargine U-300 Bedtime** | **Degludec U-100 Bedtime** | **Relative**  **(95% CI)** | **Absolute**  **(95% CI)** |
| **HbA1c＜7.0 % Three months, favor Degludec U-100 Bed** | | | | | | | | | | | |
| 1 | randomised trials | not serious^a^ | not serious | not serious | serious^b^ | none | 160/462 (34.6%) | 167/462 (36.1%) | **RR 0.96**  (0.80 to 1.14) | **14 fewer per 1,000**  (from 72 fewer to 51 more) | ⨁⨁⨁◯  Moderate |
| **HbA1c＜7.0 % Six months, favor Glargine U-300 Bed** | | | | | | | | | | | |
| 1 | randomised trials | not serious^a^ | not serious | not serious | serious^b^ | none | 225/462 (48.7%) | 206/462 (44.6%) | **RR 1.09**  (0.95 to 1.25) | **40 more per 1,000**  (from 22 fewer to 111 more) | ⨁⨁⨁◯  Moderate |
| **HbA1c Change,% Three months, favor Degludec U-100 Bed** | | | | | | | | | | | |
| 1 | randomised trials | not serious^a^ | not serious | not serious | not serious | none | 462 | 462 | - | MD **0.02 higher**  (0.09 lower to 0.13 higher) | ⨁⨁⨁⨁  High |
| **HbA1c Change,% Six months, favor Glargine U-300 Bed** | | | | | | | | | | | |
| 1 | randomised trials | not serious^a^ | not serious | not serious | not serious | none | 462 | 462 | - | MD **0.05 lower**  (0.16 lower to 0.06 higher) | ⨁⨁⨁⨁  High |
| **Hypoglycemia<3.0 Three months, favor Glargine U-300 Bed** | | | | | | | | | | | |
| 1 | randomised trials | not serious^a^ | not serious | not serious | very serious^c^ | none | 36/462 (7.8%) | 54/462 (11.7%) | **RR 0.67**  (0.45 to 1.00) | **39 fewer per 1,000**  (from 64 fewer to 0 fewer) | ⨁⨁◯◯  Low |
| **Hypoglycemia<3.0 Six months, favor Glargine U-300 Bed** | | | | | | | | | | | |
| 1 | randomised trials | not serious^a^ | not serious | not serious | very serious^d^ | none | 68/426 (16.0%) | 85/462 (18.4%) | **RR 0.80**  (0.60 to 1.07) | **37 fewer per 1,000**  (from 74 fewer to 13 more) | ⨁⨁◯◯  Low |
| **Hypoglycemia<3.9 Three months, favor Glargine U-300 Bed** | | | | | | | | | | | |
| 1 | randomised trials | not serious^a^ | not serious | not serious | serious^b^ | none | 219/462 (47.4%) | 251/462 (54.3%) | **RR 0.87**  (0.77 to 0.99) | **71 fewer per 1,000**  (from 125 fewer to 5 fewer) | ⨁⨁⨁◯  Moderate |
| **Hypoglycemia<3.9 Six months, favor Glargine U-300 Bed** | | | | | | | | | | | |
| 1 | randomised trials | not serious^a^ | not serious | not serious | not serious | none | 307/462 (66.5%) | 319/462 (69.0%) | **RR 0.96**  (0.88 to 1.05) | **28 fewer per 1,000**  (from 83 fewer to 35 more) | ⨁⨁⨁⨁  High |
| **Severe Hypoglycemia Six mon** | | | | | | | | | | | |
| 1 | randomised trials | not serious^a^ | not serious | not serious | very serious^c^ | none | 1/462 (0.2%) | 0/462 (0.0%) | **RR 3.00**  (0.12 to 73.45) | **0 fewer per 1,000**  (from 0 fewer to 0 fewer) | ⨁⨁◯◯  Low |
| **FPG Change,mmol/L Three months, favor Degludec U-100 Bed** | | | | | | | | | | | |
| 1 | randomised trials | not serious^a^ | not serious | not serious | serious^e^ | none | 462 | 462 | - | MD **0.25 higher**  (0.03 lower to 0.53 higher) | ⨁⨁⨁◯  Moderate |
| **FPG Change,mmol/L Six months, favor Degludec U-100 Bed** | | | | | | | | | | | |
| 1 | randomised trials | not serious^a^ | not serious | not serious | serious^e^ | none | 462 | 462 | - | MD **0.43 higher**  (0.13 higher to 0.73 higher) | ⨁⨁⨁◯  Moderate |
| **Nocturnal hypoglycemia<3.0,Three months, favor Glargine U-300 Bed** | | | | | | | | | | | |
| 1 | randomised trials | not serious^a^ | not serious | not serious | very serious^c^ | none | 13/462 (2.8%) | 16/462 (3.5%) | **RR 0.81**  (0.40 to 1.67) | **7 fewer per 1,000**  (from 21 fewer to 23 more) | ⨁⨁◯◯  Low |
| **Nocturnal hypoglycemia<3.0,Six months** | | | | | | | | | | | |
| 1 | randomised trials | not serious^a^ | not serious | not serious | very serious^c^ | none | 28/462 (6.1%) | 28/462 (6.1%) | **RR 1.00**  (0.60 to 1.66) | **0 fewer per 1,000**  (from 24 fewer to 40 more) | ⨁⨁◯◯  Low |
| **Nocturnal hypoglycemia<3.9,Three months, favor Degludec U-100 Bed** | | | | | | | | | | | |
| 1 | randomised trials | not serious^a^ | not serious | not serious | very serious^d^ | none | 87/462 (18.8%) | 70/462 (15.2%) | **RR 1.24**  (0.93 to 1.66) | **36 more per 1,000**  (from 11 fewer to 100 more) | ⨁⨁◯◯  Low |
| **Nocturnal hypoglycemia<3.9,Six months, favor Glargine U-300 Bed** | | | | | | | | | | | |
| 1 | randomised trials | not serious^a^ | not serious | not serious | serious^b^ | none | 132/462 (28.6%) | 133/462 (28.8%) | **RR 0.99**  (0.81 to 1.22) | **3 fewer per 1,000**  (from 55 fewer to 63 more) | ⨁⨁⨁◯  Moderate |
| **Weight change, kg Three months(Glargine U-300 Bed vs. Degludec U-100 Bed: 1.3 vs. 1.3)** | | | | | | | | | | | |
| 1 | randomised trials | not serious^a^ | not serious | not serious | very serious^f^ | none | 462 | 462 | - | MD **0**  (0.48 lower to 0.48 higher) | ⨁⨁◯◯  Low |
| **Weight change, kg Six months, favor Glargine U-300 Bed (Glargine U-300 Bed vs. Degludec U-100 Bed: 2 vs. 2.3)** | | | | | | | | | | | |
| 1 | randomised trials | not serious^a^ | not serious | not serious | very serious^f^ | none | 462 | 462 | - | MD **0.3 lower**  (0.78 lower to 0.18 higher) | ⨁⨁◯◯  Low |

**CI:** confidence interval; **MD:** mean difference; **RR:** risk ratio.

**Explanations**

a. Downgraded by 0.5 level due to ROB: there are some concerning risks of biases in 2 domains.

b. Downgraded by 1 level due to ROB and imprecision: downgraded by 0.5 due to ROB: there are some concerning risk of biases in 2 domains, downgraded by 0.5 due to imprecision: low event rate.

c. Downgraded by two levels due to imprecision: wide confidence interval.

d. Downgraded by 2 levels levels due to ROB and imprecision: downgraded by 0.5 due to ROB: there are some concerns about the risk of bias2 domains,downgraded by 1.5 due to imprecision: wide confidence interval and low event rate.

e. Downgraded by one level due to imprecision: confidence interval goes through 0.5.

f. Downgraded by two-levels due to imprecision: confidence interval goes through 2%/3% and 5%.

#### (1.1) Renal function subgroup

| **Certainty assessment** | | | | | | | **№ of patients** | | **Effect** | | **Certainty** |
| --- | --- | --- | --- | --- | --- | --- | --- | --- | --- | --- | --- |
| **№ of studies** | **Study design** | **Risk of bias** | **Inconsistency** | **Indirectness** | **Imprecision** | **Other considerations** | **Glargine U-300 Bedtime** | **Degludec U-100 Bedtime Renal function subgroup** | **Relative**  **(95% CI)** | **Absolute**  **(95% CI)** |
| **Hypoglycemia<3.0 - eGFR≥90 mL/min/1.73m2, favor Glargine U-300 Bedtime** | | | | | | | | | | | |
| 1 | randomised trials | not serious^a^ | not serious | not serious | very serious^b^ | none | 29/246 (11.8%) | 35/221 (15.8%) | **RR 0.74**  (0.47 to 1.18) | **41 fewer per 1,000**  (from 84 fewer to 29 more) | ⨁⨁◯◯  Low |
| **Hypoglycemia<3.0 - eGFR:60-90 mL/min/1.73m2, favor Glargine U-300 Bedtime** | | | | | | | | | | | |
| 1 | randomised trials | not serious^a^ | not serious | not serious | very serious^b^ | none | 27/172 (15.7%) | 36/193 (18.7%) | **RR 0.84**  (0.53 to 1.33) | **30 fewer per 1,000**  (from 88 fewer to 62 more) | ⨁⨁◯◯  Low |
| **Hypoglycemia<3.0 - eGFR<60 mL/min/1.73m2, favor Glargine U-300 Bedtime** | | | | | | | | | | | |
| 1 | randomised trials | not serious^a^ | not serious | not serious | very serious^b^ | none | 13/47 (27.7%) | 14/49 (28.6%) | **RR 0.97**  (0.51 to 1.84) | **9 fewer per 1,000**  (from 140 fewer to 240 more) | ⨁⨁◯◯  Low |
| **Hypoglycemia<3.9 - eGFR≥90 mL/min/1.73m2, favor Glargine U-300 Bedtime** | | | | | | | | | | | |
| 1 | randomised trials | not serious^a^ | not serious | not serious | serious^c^ | none | 147/246 (59.8%) | 144/221 (65.2%) | **RR 0.92**  (0.80 to 1.06) | **52 fewer per 1,000**  (from 130 fewer to 39 more) | ⨁⨁⨁◯  Moderate |
| **Hypoglycemia<3.9 - eGFR:60-90 mL/min/1.73m2, favor Degludec U-100 Bedtime** | | | | | | | | | | | |
| 1 | randomised trials | not serious^a^ | not serious | not serious | serious^c^ | none | 123/172 (71.5%) | 137/193 (71.0%) | **RR 1.01**  (0.88 to 1.15) | **7 more per 1,000**  (from 85 fewer to 106 more) | ⨁⨁⨁◯  Moderate |
| **Hypoglycemia<3.9 - eGFR<60 mL/min/1.73m2, favor Degludec U-100 Bedtime** | | | | | | | | | | | |
| 1 | randomised trials | not serious^a^ | not serious | not serious | serious^c^ | none | 38/47 (80.9%) | 39/49 (79.6%) | **RR 1.02**  (0.83 to 1.24) | **16 more per 1,000**  (from 135 fewer to 191 more) | ⨁⨁⨁◯  Moderate |
| **Nocturnal Hypoglycemia<3.0 - eGFR≥90 mL/min/1.73m2, favor Glargine U-300 Bedtime** | | | | | | | | | | | |
| 1 | randomised trials | not serious^a^ | not serious | not serious | very serious^b^ | none | 8/246 (3.3%) | 13/221 (5.9%) | **RR 0.55**  (0.23 to 1.31) | **26 fewer per 1,000**  (from 45 fewer to 18 more) | ⨁⨁◯◯  Low |
| **Nocturnal Hypoglycemia<3.0 - eGFR:60-90 mL/min/1.73m2, favor Degludec U-100 Bedtime** | | | | | | | | | | | |
| 1 | randomised trials | not serious^a^ | not serious | not serious | very serious^b^ | none | 12/172 (7.0%) | 8/193 (4.1%) | **RR 1.68**  (0.70 to 4.02) | **28 more per 1,000**  (from 12 fewer to 125 more) | ⨁⨁◯◯  Low |
| **Nocturnal Hypoglycemia<3.0 - eGFR<60 mL/min/1.73m2, favor Degludec U-100 Bedtime** | | | | | | | | | | | |
| 1 | randomised trials | not serious^a^ | not serious | not serious | very serious^b^ | none | 8/47 (17.0%) | 7/49 (14.3%) | **RR 1.19**  (0.47 to 3.03) | **27 more per 1,000**  (from 76 fewer to 290 more) | ⨁⨁◯◯  Low |
| **Nocturnal Hypoglycemia<3.9 - eGFR≥90 mL/min/1.73m^2^, favor Glargine U-300 Bedtime** | | | | | | | | | | | |
| 1 | randomised trials | not serious^a^ | not serious | not serious | very serious^d^ | none | 63/246 (25.6%) | 71/221 (32.1%) | **RR 0.80**  (0.60 to 1.06) | **64 fewer per 1,000**  (from 129 fewer to 19 more) | ⨁⨁◯◯  Low |
| **Nocturnal Hypoglycemia<3.9 - eGFR:60-90 mL/min/1.73m2, favor Degludec U-100 Bedtime** | | | | | | | | | | | |
| 1 | randomised trials | not serious^a^ | not serious | not serious | very serious^d^ | none | 52/172 (30.2%) | 46/193 (23.8%) | **RR 1.27**  (0.90 to 1.78) | **64 more per 1,000**  (from 24 fewer to 186 more) | ⨁⨁◯◯  Low |
| **Nocturnal Hypoglycemia<3.9 - eGFR<60 mL/min/1.73m2, favor Degludec U-100 Bedtime** | | | | | | | | | | | |
| 1 | randomised trials | not serious^a^ | not serious | not serious | very serious^b^ | none | 18/47 (38.3%) | 16/49 (32.7%) | **RR 1.17**  (0.68 to 2.02) | **56 more per 1,000**  (from 104 fewer to 333 more) | ⨁⨁◯◯  Low |

**CI:** confidence interval; **RR:** risk ratio.

**Explanations**

a. Downgraded by 0.5 level due to ROB: there are some concerning risks of biases in 2 domains.

b. Downgraded by two levels due to imprecision: wide confidence interval.

c. Downgraded by 1 level due to ROB and imprecision: downgraded by 0.5 due to ROB: there are some concerning risks of biases in 2 domains, downgraded by 0.5 due to imprecision: low event rate.

d. Downgraded by 2 levels due to ROB and imprecision: downgraded by 0.5 due to ROB: there are some concerning risks of biases in 2 domains, downgraded by 1.5 due to imprecision: wide confidence interval and low event rate.

#### (1.2) Age subgroup

| **Certainty assessment** | | | | | | | **№ of patients** | | **Effect** | | **Certainty** |
| --- | --- | --- | --- | --- | --- | --- | --- | --- | --- | --- | --- |
| **№ of studies** | **Study design** | **Risk of bias** | **Inconsistency** | **Indirectness** | **Imprecision** | **Other considerations** | **Glargine U-300 Bedtime** | **Degludec U-100 Bedtime Age subgroup** | **Relative**  **(95% CI)** | **Absolute**  **(95% CI)** |
| **HbA1c endpoint,% Age<65 -Three months, favor Degludec U-100 Bedtime** | | | | | | | | | | | |
| 1 | randomised trials | not serious^a^ | not serious | not serious | very serious^b^ | none | 295 | 297 | - | MD **0.14 higher**  (2.19 lower to 2.47 higher) | ⨁⨁◯◯  Low |
| **HbA1c endpoint,% Age≥65 - Three months, favor Degludec U-100 Bedtime** | | | | | | | | | | | |
| 1 | randomised trials | not serious^a^ | not serious | not serious | very serious^b^ | none | 167 | 165 | - | MD **0.02 higher**  (2.06 lower to 2.1 higher) | ⨁⨁◯◯  Low |
| **HbA1c endpoint,% Age<65 - Six months, favor Degludec U-100 Bedtime** | | | | | | | | | | | |
| 1 | randomised trials | not serious^a^ | not serious | not serious | very serious^b^ | none | 295 | 297 | - | MD **0.03 higher**  (2.2 lower to 2.26 higher) | ⨁⨁◯◯  Low |
| **HbA1c endpoint,% Age≥65 -Six months, favor Glargine U-300 Bedtime** | | | | | | | | | | | |
| 1 | randomised trials | not serious^a^ | not serious | not serious | very serious^b^ | none | 167 | 165 | - | MD **0.06 lower**  (2.07 lower to 1.95 higher) | ⨁⨁◯◯  Low |
| **Hypoglycemia<3.0 Age<65 -Three months, favor Glargine U-300 Bedtime** | | | | | | | | | | | |
| 1 | randomised trials | not serious^a^ | not serious | not serious | very serious^c^ | none | 24/295 (8.1%) | 33/297 (11.1%) | **RR 0.73**  (0.44 to 1.21) | **30 fewer per 1,000**  (from 62 fewer to 23 more) | ⨁⨁◯◯  Low |
| **Hypoglycemia<3.0 Age<65 -Six months, favor Glargine U-300 Bedtime** | | | | | | | | | | | |
| 1 | randomised trials | not serious^a^ | not serious | not serious | very serious^d^ | none | 42/295 (14.2%) | 58/297 (19.5%) | **RR 0.73**  (0.51 to 1.05) | **53 fewer per 1,000**  (from 96 fewer to 10 more) | ⨁⨁◯◯  Low |
| **Hypoglycemia<3.0 Age ≥65 -Three months, favor Glargine U-300 Bedtime** | | | | | | | | | | | |
| 1 | randomised trials | not serious^a^ | not serious | not serious | very serious^c^ | none | 12/167 (7.2%) | 21/165 (12.7%) | **RR 0.56**  (0.29 to 1.11) | **56 fewer per 1,000**  (from 90 fewer to 14 more) | ⨁⨁◯◯  Low |
| **Hypoglycemia<3.0 Age ≥65 -Six months, favor Glargine U-300 Bedtime** | | | | | | | | | | | |
| 1 | randomised trials | not serious^a^ | not serious | not serious | very serious^c^ | none | 26/167 (15.6%) | 27/165 (16.4%) | **RR 0.95**  (0.58 to 1.56) | **8 fewer per 1,000**  (from 69 fewer to 92 more) | ⨁⨁◯◯  Low |
| **Hypoglycemia<3.9 Age<65 -Three months, favor Glargine U-300 Bedtime** | | | | | | | | | | | |
| 1 | randomised trials | not serious^a^ | not serious | not serious | very serious^d^ | none | 131/295 (44.4%) | 150/297 (50.5%) | **RR 0.88**  (0.74 to 1.04) | **61 fewer per 1,000**  (from 131 fewer to 20 more) | ⨁⨁◯◯  Low |
| **Hypoglycemia<3.9 Age ≥65 -Three months, favor Glargine U-300 Bedtime** | | | | | | | | | | | |
| 1 | randomised trials | not serious^a^ | not serious | not serious | very serious^d^ | none | 88/167 (52.7%) | 101/165 (61.2%) | **RR 0.86**  (0.71 to 1.04) | **86 fewer per 1,000**  (from 178 fewer to 24 more) | ⨁⨁◯◯  Low |
| **Hypoglycemia<3.9 Age<65 -Six months, favor Glargine U-300 Bedtime** | | | | | | | | | | | |
| 1 | randomised trials | not serious^a^ | not serious | not serious | serious^e^ | none | 181/295 (61.4%) | 195/297 (65.7%) | **RR 0.93**  (0.83 to 1.06) | **46 fewer per 1,000**  (from 112 fewer to 39 more) | ⨁⨁⨁◯  Moderate |
| **Hypoglycemia<3.9 Age ≥65 -Six months, favor Degludec U-100 Bedtime** | | | | | | | | | | | |
| 1 | randomised trials | not serious^a^ | not serious | not serious | serious^e^ | none | 126/167 (75.4%) | 124/165 (75.2%) | **RR 1.00**  (0.89 to 1.14) | **0 fewer per 1,000**  (from 83 fewer to 105 more) | ⨁⨁⨁◯  Moderate |
| **Serve Hypoglycemia Age <65 -Six months, favor Degludec U-100 Bedtime** | | | | | | | | | | | |
| 1 | randomised trials | not serious^a^ | not serious | not serious | very serious^c^ | none | 1/297 (0.3%) | 0/295 (0.0%) | **RR 2.98**  (0.12 to 72.85) | **0 fewer per 1,000**  (from 0 fewer to 0 fewer) | ⨁⨁◯◯  Low |
| **Nocturnal hypoglycemia<3.9 Age <65 -Three months, favor Glargine U-300 Bedtime** | | | | | | | | | | | |
| 1 | randomised trials | not serious^a^ | not serious | not serious | very serious^d^ | none | 43/295 (14.6%) | 52/297 (17.5%) | **RR 0.83**  (0.57 to 1.21) | **30 fewer per 1,000**  (from 75 fewer to 37 more) | ⨁⨁◯◯  Low |
| **Nocturnal hypoglycemia<3.9 Age ≥65 -Three months, favor Glargine U-300 Bedtime** | | | | | | | | | | | |
| 1 | randomised trials | not serious^a^ | not serious | not serious | very serious^c^ | none | 27/167 (16.2%) | 35/165 (21.2%) | **RR 0.76**  (0.48 to 1.20) | **51 fewer per 1,000**  (from 110 fewer to 42 more) | ⨁⨁◯◯  Low |
| **Nocturnal hypoglycemia<3.9 Age <65 -Six months, favor Glargine U-300 Bedtime** | | | | | | | | | | | |
| 1 | randomised trials | not serious^a^ | not serious | not serious | very serious^d^ | none | 79/295 (26.8%) | 85/297 (28.6%) | **RR 0.94**  (0.72 to 1.21) | **17 fewer per 1,000**  (from 80 fewer to 60 more) | ⨁⨁◯◯  Low |
| **Nocturnal hypoglycemia<3.9 Age ≥65 -Six months, favor Degludec U-100 Bedtime** | | | | | | | | | | | |
| 1 | randomised trials | not serious^a^ | not serious | not serious | very serious^d^ | none | 53/167 (31.7%) | 48/165 (29.1%) | **RR 1.09**  (0.79 to 1.51) | **26 more per 1,000**  (from 61 fewer to 148 more) | ⨁⨁◯◯  Low |

**CI:** confidence interval; **MD:** mean difference; **RR:** risk ratio.

**Explanations**

a. Downgraded by 0.5 level due to ROB: there are some concerning risks of biases in 2 domains.

b. Downgraded by 2 levels due to imprecision: confidencenterval go through 0.4 and 1.

c. Downgraded by two levels due to imprecision: wide confidence interval.

d. Downgraded by 2 levels due to ROB and imprecision: downgraded by 0.5 due to ROB: there are some concerning risks of biases in 2 domains, downgraded by 1.5 due to imprecision: wide confidence interval and low event rate.

e. Downgraded by 1 level due to ROB and imprecision: downgraded by 0.5 due to ROB: there are some concerning risks of biases in 2 domains, downgraded by 0.5 due to imprecision: low event rate.

### Degludec U-100 vs. Glargine U-100

| **Certainty assessment** | | | | | | | **№ of patients** | | **Effect** | | **Certainty** |
| --- | --- | --- | --- | --- | --- | --- | --- | --- | --- | --- | --- |
| **№ of studies** | **Study design** | **Risk of bias** | **Inconsistency** | **Indirectness** | **Imprecision** | **Other considerations** | **Degludec U-100 Bedtime** | **Glargine U-100 Bedtime** | **Relative**  **(95% CI)** | **Absolute**  **(95% CI)** |
| **HbA1c＜7.0 % Six months, favor Glargine U-100 Bed** | | | | | | | | | | | |
| 2 | randomised trials | not serious^a^ | serious^b^ | not serious | serious^c^ | none | 419/844 (49.6%) | 214/424 (50.5%) | **RR 0.96**  (0.77 to 1.91) | **20 fewer per 1,000**  (from 116 fewer to 459 more) | ⨁⨁◯◯  Low |
| **HbA1c＜7.0 % Twelve months, favor Glargine U-100 Bed** | | | | | | | | | | | |
| 1 | randomised trials | not serious^a^ | not serious | not serious | not serious | none | 400/773 (51.7%) | 139/257 (54.1%) | **RR 0.96**  (0.84 to 1.09) | **22 fewer per 1,000**  (from 87 fewer to 49 more) | ⨁⨁⨁⨁  High |
| **HbA1c Change,% Six months, favor Degludec U-100 Bed** | | | | | | | | | | | |
| 2 | randomised trials | not serious^a^ | serious^b^ | not serious | not serious | none | 844 | 424 | - | MD **0.01 lower**  (0.21 lower to 0.2 higher) | ⨁⨁⨁◯  Moderate |
| **HbA1c Change,% Twelve months, favor Glargine U-100 Bed** | | | | | | | | | | | |
| 1 | randomised trials | not serious^a^ | not serious | not serious | not serious | none | 773 | 257 | - | MD **0.13 higher**  (0.01 lower to 0.27 higher) | ⨁⨁⨁⨁  High |
| **Hypoglycemia<3.0 Six months, favor Degludec U-100 Bed** | | | | | | | | | | | |
| 2 | randomised trials | not serious^a^ | not serious | not serious | not serious | none | 270/837 (32.3%) | 157/424 (37.0%) | **RR 0.89**  (0.76 to 1.03) | **41 fewer per 1,000**  (from 89 fewer to 11 more) | ⨁⨁⨁⨁  High |
| **Hypoglycemia<3.0 Twelve mon** | | | | | | | | | | | |
| 1 | randomised trials | not serious^a^ | not serious | not serious | not serious | none | 356/766 (46.5%) | 119/257 (46.3%) | **RR 1.00**  (0.86 to 1.17) | **0 fewer per 1,000**  (from 65 fewer to 79 more) | ⨁⨁⨁⨁  High |
| **Severe Hypoglycemia Six months, favor Degludec U-100 Bed** | | | | | | | | | | | |
| 2 | randomised trials | not serious^a^ | not serious | not serious | very serious^d^ | none | 2/837 (0.2%) | 3/424 (0.7%) | **RR 0.38**  (0.07 to 1.99) | **4 fewer per 1,000**  (from 7 fewer to 7 more) | ⨁⨁◯◯  Low |
| **Severe Hypoglycemia Twelve months, favor Degludec U-100 Bed** | | | | | | | | | | | |
| 1 | randomised trials | not serious^a^ | not serious | not serious | very serious^e^ | none | 2/766 (0.3%) | 5/257 (1.9%) | **RR 0.13**  (0.03 to 0.69) | **17 fewer per 1,000**  (from 19 fewer to 6 fewer) | ⨁⨁◯◯  Low |
| **FPG Change,mmol/L Three months, favor Degludec U-100 Bed** | | | | | | | | | | | |
| 1 | randomised trials | not serious^a^ | not serious | not serious | not serious | none | 773 | 257 | - | MD **0.5 lower**  (1 lower to 0 ) | ⨁⨁⨁⨁  High |
| **FPG Change,mmol/L Six months, favor Degludec U-100 Bed** | | | | | | | | | | | |
| 2 | randomised trials | not serious^a^ | not serious | not serious | not serious | none | 844 | 424 | - | MD **0.03 lower**  (0.32 lower to 0.26 higher) | ⨁⨁⨁⨁  High |
| **FPG Change,mmol/L Twelve months, favor Degludec U-100 Bed** | | | | | | | | | | | |
| 1 | randomised trials | not serious^a^ | not serious | not serious | not serious | none | 762 | 256 | - | MD **0.46 lower**  (0.87 lower to 0.05 lower) | ⨁⨁⨁⨁  High |
| **Nocturnal hypoglycemia<3.0 Six months, favor Degludec U-100 Bed** | | | | | | | | | | | |
| 2 | randomised trials | not serious^a^ | not serious | not serious | very serious^e^ | none | 98/837 (11.7%) | 60/424 (14.2%) | **RR 0.83**  (0.62 to 1.12) | **24 fewer per 1,000**  (from 54 fewer to 17 more) | ⨁⨁◯◯  Low |
| **Nocturnal hypoglycemia<3.0 Twelve months, favor Degludec U-100 Bed** | | | | | | | | | | | |
| 1 | randomised trials | not serious^a^ | not serious | not serious | very serious^d^ | none | 106/766 (13.8%) | 39/257 (15.2%) | **RR 0.91**  (0.65 to 1.28) | **14 fewer per 1,000**  (from 53 fewer to 42 more) | ⨁⨁◯◯  Low |
| **Weight change,kg Six months, favor Glargine U-100 Bed (1.Gough 2013-Degludec U-100 Bed vs. Glargine U-100 Bed: 1.9 vs. 1.5; 2. Onishi 2013-Degludec U-100 Bed vs. Glargine U-100 Bed: 1.3 vs. 1.4; 3. Pan 2016-Degludec U-100 Bed vs. Glargine U-100 Bed: 2.2 vs. 1.8)** | | | | | | | | | | | |
| 2 | randomised trials | not serious^a^ | not serious | not serious | very serious^f^ | none | 842 | 424 | - | MD **0.21 higher**  (0.27 lower to 0.68 higher) | ⨁⨁◯◯  Low |
| **Weight change,kg Twelve months, favor Glargine U-100 Bed (Degludec U-100 Bed vs. Glargine U-100 Bed: 2.4 vs. 2.1)** | | | | | | | | | | | |
| 1 | randomised trials | not serious^a^ | not serious | not serious | very serious^f^ | none | 766 | 257 | - | MD **0.3 higher**  (0.29 lower to 0.89 higher) | ⨁⨁◯◯  Low |

**CI:** confidence interval; **MD:** mean difference; **RR:** risk ratio.

**Explanations**

a. Downgraded by 0.5 level due to ROB: there are some concerning risks of biases in 2 domains.

b. Downgraded by 1 level due to ROB and imprecision: downgraded by 0.5 due to ROB: there are some concerning risks of biases in 2 domains, downgraded by 0.5 level due to heterogeneity. I^2^= 60%-80%.

c. Downgraded by one level due to imprecision: wide confidence interval.

d. Downgraded by two levels due to imprecision: wide confidence interval.

e. Downgraded by 2 levels due to ROB and imprecision: downgraded by 0.5 due to ROB: there are some concerning risks of biases in 2 domains, downgraded by 1.5 due to imprecision: wide confidence interval and low event rate.

f. Downgraded by 2 levels due to imprecision: confidence interval goes through 2%/3%/5%.

### Glargine U-300 vs. Glargine U-100

| **Certainty assessment** | | | | | | | **№ of patients** | | **Effect** | | **Certainty** |
| --- | --- | --- | --- | --- | --- | --- | --- | --- | --- | --- | --- |
| **№ of studies** | **Study design** | **Risk of bias** | **Inconsistency** | **Indirectness** | **Imprecision** | **Other considerations** | **Glargine U-300 Bedtime** | **Glargine U-100 Bedtime** | **Relative**  **(95% CI)** | **Absolute**  **(95% CI)** |
| **HbA1c＜7.0 % Six months, favor Glargine U-300 Bed** | | | | | | | | | | | |
| 2 | randomised trials | not serious^a^ | not serious | not serious | not serious | none | 389/829 (46.9%) | 296/631 (46.9%) | **RR 1.00**  (0.90 to 1.12) | **0 fewer per 1,000**  (from 47 fewer to 56 more) | ⨁⨁⨁⨁  High |
| **HbA1c＜7.0 % Twelve months, favor Glargine U-300 Bed** | | | | | | | | | | | |
| 1 | randomised trials | not serious^a^ | not serious | not serious | very serious^b^ | none | 125/432 (28.9%) | 101/430 (23.5%) | **RR 1.23**  (0.98 to 1.54) | **54 more per 1,000**  (from 5 fewer to 127 more) | ⨁⨁◯◯  Low |
| **HbA1c Change,% Six months, favor Glargine U-100 Bed** | | | | | | | | | | | |
| 2 | randomised trials | not serious^a^ | not serious | not serious | not serious | none | 829 | 631 | - | MD **0.03 higher**  (0.09 lower to 0.16 higher) | ⨁⨁⨁⨁  High |
| **HbA1c Change,% Twelve months, favor Glargine U-300 Bed** | | | | | | | | | | | |
| 1 | randomised trials | not serious^a^ | not serious | not serious | not serious | none | 439 | 439 | - | MD **0.08 lower**  (0.22 lower to 0.06 higher) | ⨁⨁⨁⨁  High |
| **Hypoglycemia<3.0 Twelve months, favor Glargine U-300 Bed** | | | | | | | | | | | |
| 1 | randomised trials | not serious^a^ | not serious | not serious | very serious^c^ | none | 60/432 (13.9%) | 95/430 (22.1%) | **RR 0.63**  (0.47 to 0.84) | **82 fewer per 1,000**  (from 117 fewer to 35 fewer) | ⨁⨁◯◯  Low |
| **Hypoglycemia<3.9 Six months, favor Glargine U-300 Bed** | | | | | | | | | | | |
| 2 | randomised trials | not serious^a^ | not serious | not serious | not serious | none | 471/832 (56.6%) | 376/639 (58.8%) | **RR 0.91**  (0.84 to 0.99) | **53 fewer per 1,000**  (from 94 fewer to 6 fewer) | ⨁⨁⨁⨁  High |
| **Hypoglycemia<3.9 Twelve months, favor Glargine U-300 Bed** | | | | | | | | | | | |
| 1 | randomised trials | not serious^a^ | not serious | not serious | not serious | none | 242/432 (56.0%) | 262/430 (60.9%) | **RR 0.92**  (0.82 to 1.03) | **49 fewer per 1,000**  (from 110 fewer to 18 more) | ⨁⨁⨁⨁  High |
| **Severe Hypoglycemia Six months** | | | | | | | | | | | |
| 1 | randomised trials | not serious^a^ | not serious | not serious | very serious^c^ | none | 4/435 (0.9%) | 4/438 (0.9%) | **RR 1.01**  (0.25 to 4.00) | **0 fewer per 1,000**  (from 7 fewer to 27 more) | ⨁⨁◯◯  Low |
| **Severe Hypoglycemia Twelve months, favor Glargine U-300 Bed** | | | | | | | | | | | |
| 1 | randomised trials | not serious^a^ | not serious | not serious | very serious^c^ | none | 6/432 (1.4%) | 9/430 (2.1%) | **RR 0.66**  (0.24 to 1.85) | **7 fewer per 1,000**  (from 16 fewer to 18 more) | ⨁⨁◯◯  Low |
| **FPG<5.6 Six months, favor Glargine U-100 Bed** | | | | | | | | | | | |
| 1 | randomised trials | not serious^a^ | not serious | not serious | very serious^b^ | none | 113/432 (26.2%) | 127/430 (29.5%) | **RR 0.89**  (0.71 to 1.10) | **32 fewer per 1,000**  (from 86 fewer to 30 more) | ⨁⨁◯◯  Low |
| **FPG≤6.7 Six months, favor Glargine U-100 Bed** | | | | | | | | | | | |
| 1 | randomised trials | not serious^a^ | not serious | not serious | serious^d^ | none | 217/432 (50.2%) | 231/430 (53.7%) | **RR 0.94**  (0.82 to 1.06) | **32 fewer per 1,000**  (from 97 fewer to 32 more) | ⨁⨁⨁◯  Moderate |
| **FPG Change,mmol/L Six months, favor Glargine U-100 Bed** | | | | | | | | | | | |
| 2 | randomised trials | not serious^a^ | not serious | not serious | not serious | none | 817 | 626 | - | MD **0.24 higher**  (0.09 higher to 0.38 higher) | ⨁⨁⨁⨁  High |
| **FPG Change,mmol/L Twelve months, favor Glargine U-100 Bed** | | | | | | | | | | | |
| 1 | randomised trials | not serious^a^ | not serious | not serious | not serious | none | 439 | 439 | - | MD **0.07 higher**  (0.26 lower to 0.4 higher) | ⨁⨁⨁⨁  High |
| **Nocturnal hypoglycemia<3.9 Six months, favor Glargine U-300 Bed** | | | | | | | | | | | |
| 2 | randomised trials | not serious^a^ | not serious | not serious | very serious^b^ | none | 244/832 (29.3%) | 204/639 (31.9%) | **RR 0.81**  (0.70 to 0.94) | **61 fewer per 1,000**  (from 96 fewer to 19 fewer) | ⨁⨁◯◯  Low |
| **Nocturnal hypoglycemia<3.9,Twelve months, favor Glargine U-300 Bed** | | | | | | | | | | | |
| 1 | randomised trials | not serious^a^ | not serious | not serious | very serious^b^ | none | 108/432 (25.0%) | 125/430 (29.1%) | **RR 0.86**  (0.69 to 1.07) | **41 fewer per 1,000**  (from 90 fewer to 20 more) | ⨁⨁◯◯  Low |
| **Weight change,kg Six months, favor Glargine U-300 Bed (1. Bolli 2015: Glargine U-300 Bed vs. Glargine U-100 Bed: 0.49 vs. 0.71; 2. Ji 2020: Glargine U-300 Bed vs. Glargine U-100 Bed: 1.75 vs 1.69)** | | | | | | | | | | | |
| 2 | randomised trials | not serious^a^ | not serious | not serious | very serious^e^ | none | 829 | 631 | - | MD **0.06 lower**  (0.38 lower to 0.26 higher) | ⨁⨁◯◯  Low |
| **Weight change,kg Twelve months, favor Glargine U-300 Bed (Bolli 2017-Glargine U-300 Bed vs. Glargine U-100 Bed: 0.97 vs. 1.2)** | | | | | | | | | | | |
| 1 | randomised trials | not serious^a^ | not serious | not serious | very serious^e^ | none | 432 | 430 | - | MD **0.23 lower**  (0.80 lower to 0.34 higher) | ⨁⨁◯◯  Low |
| **HbA1c Change,% Three months** | | | | | | | | | | | |
| 1 | randomised trials | not serious^a^ | not serious | not serious | serious^f^ | none | 397 | 201 | - | MD **0**  (0 to 0 ) | ⨁⨁⨁◯  Moderate |
| **Hypoglycemia<3.0 Three months, favor Glargine U-300** | | | | | | | | | | | |
| 1 | randomised trials | not serious^a^ | not serious | not serious | very serious^g^ | none | 228/397 (57.4%) | 139/201 (69.2%) | **RR 0.83**  (0.73 to 0.94) | **118 fewer per 1,000**  (from 187 fewer to 41 fewer) | ⨁⨁◯◯  Low |
| **Hypoglycemia<3.0 Six months, favor Glargine U-300** | | | | | | | | | | | |
| 1 | randomised trials | not serious^a^ | not serious | not serious | serious^h^ | none | 291/397 (73.3%) | 160/201 (79.6%) | **RR 0.92**  (0.84 to 1.01) | **64 fewer per 1,000**  (from 127 fewer to 8 more) | ⨁⨁⨁◯  Moderate |
| **Hypoglycemia<3.9 Three months, favor Glargine U-300** | | | | | | | | | | | |
| 1 | randomised trials | not serious | not serious | not serious | very serious^b^ | none | 197/397 (49.6%) | 118/201 (58.7%) | **RR 0.85**  (0.73 to 0.98) | **88 fewer per 1,000**  (from 159 fewer to 12 fewer) | ⨁⨁◯◯  Low |
| **FPG Change,mmol/L Three months, favor Glargine U-100** | | | | | | | | | | | |
| 1 | randomised trials | not serious | not serious | not serious | very serious^i^ | none | 385 | 196 | - | MD **0.2 higher**  (0.2 lower to 0.6 higher) | ⨁⨁◯◯  Low |
| **Nocturnal hypoglycemia<3.0,Three months, favor Glargine U-100** | | | | | | | | | | | |
| 1 | randomised trials | not serious | not serious | not serious | very serious^j^ | none | 12/397 (3.0%) | 5/201 (2.5%) | **RR 1.22**  (0.43 to 3.40) | **5 more per 1,000**  (from 14 fewer to 60 more) | ⨁⨁◯◯  Low |
| **Nocturnal hypoglycemia<3.9,Three months, favor Glargine U-300** | | | | | | | | | | | |
| 1 | randomised trials | not serious | not serious | not serious | very serious^b^ | none | 89/397 (22.4%) | 67/201 (33.3%) | **RR 0.67**  (0.51 to 0.88) | **110 fewer per 1,000**  (from 163 fewer to 40 fewer) | ⨁⨁◯◯  Low |

**CI:** confidence interval; **MD:** mean difference; **RR:** risk ratio.

**Explanations**

a. Downgraded by 0.5 level due to ROB: there are some concerning risks of biases in 2 domains.

b. Downgraded by 2 levels due to ROB and imprecision: downgraded by 0.5 due to ROB: there are some concerning risks of biases in 2 domains, downgraded by 1.5 due to imprecision: wide confidence interval and low event rate.

c. Downgraded by two levels due to imprecision: wide confidence interval.

d. Downgraded by 1 level due to ROB and imprecision: downgraded by 0.5 due to ROB: there are some concerning risks of biases in 2 domains, downgraded by 0.5 due to imprecision: low event rate.

e. Downgraded by 2 levels due to imprecision: confidence interval goes through 2%/3%/5%.

f. Downgraded by 1 level due to ROB and imprecision: downgraded by 0.5 due to ROB: there are some concerning risks of biases in 2 domains, downgraded by 0.5 due to imprecision: small sample size (n = 598).

g. Downgraded by 2 levels due to ROB and imprecision: downgraded by 0.5 due to ROB: there are some concerning risks of biases in 2 domains, downgraded by 1.5 due to imprecision: wide confidence interval and low event rate.

h. Downgraded by 1 level due to ROB and imprecision: downgraded by 0.5 due to ROB: there are some concerning risks of biases in 2 domains, downgraded by 0.5 due to imprecision: low event rate.

i. Downgraded by 2 levels due to ROB and imprecision: downgraded by 0.5 due to ROB: there are some concerning risks of biases in 2 domains, downgraded by 1.5 due to imprecision: confidence interval go through 0.5 and small sample size (n = 598).

j. Downgraded by two levels due to imprecision: wide confidence interval.

### Glargine U - 300 vs. NPH

| **Certainty assessment** | | | | | | | **№ of patients** | | **Effect** | | **Certainty** |
| --- | --- | --- | --- | --- | --- | --- | --- | --- | --- | --- | --- |
| **№ of studies** | **Study design** | **Risk of bias** | **Inconsistency** | **Indirectness** | **Imprecision** | **Other considerations** | **Glargine U-300 Bedtime** | **NPH Bedtime** | **Relative**  **(95% CI)** | **Absolute**  **(95% CI)** |
| **HbA1c＜7.0 % Six months** | | | | | | | | | | | |
| 1 | randomised trials | not serious^a^ | not serious | not serious | very serious^b^ | none | 6/23 (26.1%) | 6/23 (26.1%) | **RR 1.00**  (0.38 to 2.65) | **0 fewer per 1,000**  (from 162 fewer to 430 more) | ⨁⨁◯◯  Low |
| **HbA1c Endpoint,% Six months, favor NPH Bed** | | | | | | | | | | | |
| 1 | randomised trials | not serious^a^ | not serious | not serious | very serious^c^ | none | 23 | 23 | - | MD **0.01 higher**  (0.38 lower to 0.4 higher) | ⨁⨁◯◯  Low |
| **Hypoglycemia<3.0 Six months, favor Glargine U-300 Bed** | | | | | | | | | | | |
| 1 | randomised trials | not serious^a^ | not serious | not serious | very serious^b^ | none | 1/23 (4.3%) | 5/23 (21.7%) | **RR 0.20**  (0.03 to 1.58) | **174 fewer per 1,000**  (from 211 fewer to 126 more) | ⨁⨁◯◯  Low |
| **Hypoglycemia<3.9 Three months, favor Glargine U-300 Bed** | | | | | | | | | | | |
| 1 | randomised trials | not serious^a^ | not serious | not serious | very serious^d^ | none | 7/23 (30.4%) | 17/23 (73.9%) | **RR 0.41**  (0.21 to 0.80) | **436 fewer per 1,000**  (from 584 fewer to 148 fewer) | ⨁⨁◯◯  Low |
| **Hypoglycemia<3.9 Six months, favor Glargine U-300 Bed** | | | | | | | | | | | |
| 1 | randomised trials | not serious^a^ | not serious | not serious | very serious^d^ | none | 6/23 (26.1%) | 17/23 (73.9%) | **RR 0.35**  (0.17 to 0.73) | **480 fewer per 1,000**  (from 613 fewer to 200 fewer) | ⨁⨁◯◯  Low |
| **Severe Hypoglycemia Six months** | | | | | | | | | | | |
| 1 | randomised trials | not serious^a^ | not serious | not serious |  | none | 0/23 (0.0%) | 0/23 (0.0%) | not estimable |  | - |
| **Nocturnal hypoglycemia<3.0 Six months, favor Glargine U-300 Bed** | | | | | | | | | | | |
| 1 | randomised trials | not serious^a^ | not serious | not serious | very serious^b^ | none | 0/23 (0.0%) | 4/23 (17.4%) | **RR 0.11**  (0.01 to 1.95) | **155 fewer per 1,000**  (from 172 fewer to 165 more) | ⨁⨁◯◯  Low |
| **Nocturnal hypoglycemia<3.9 Three months, favor Glargine U-300 Bed** | | | | | | | | | | | |
| 1 | randomised trials | not serious^a^ | not serious | not serious | very serious^d^ | none | 0/23 (0.0%) | 10/23 (43.5%) | **RR 0.05**  (0.00 to 0.77) | **413 fewer per 1,000**  (from 100 fewer to --) | ⨁⨁◯◯  Low |
| **Nocturnal hypoglycemia<3.9 Six months, favor Glargine U-300 Bed** | | | | | | | | | | | |
| 1 | randomised trials | not serious^a^ | not serious | not serious | very serious^d^ | none | 1/23 (4.3%) | 9/23 (39.1%) | **RR 0.11**  (0.02 to 0.81) | **348 fewer per 1,000**  (from 383 fewer to 74 fewer) | ⨁⨁◯◯  Low |
| **FPG Endpoint,mmol/L Six months, favor Glargine U-300 Bed** | | | | | | | | | | | |
| 1 | randomised trials | not serious^a^ | not serious | not serious | very serious^c^ | none | 23 | 23 | - | MD **0.44 lower**  (1.61 lower to 0.73 higher) | ⨁⨁◯◯  Low |
| **Weight change,kg Six months, favor NPH Bed (Glargine U-300 Bed vs. NPH Bed: 1.5 vs. 1.2)** | | | | | | | | | | | |
| 1 | randomised trials | not serious^a^ | not serious | not serious | very serious^e^ | none | 23 | 23 | - | MD **0.3 higher**  (1.14 lower to 1.74 higher) | ⨁⨁◯◯  Low |
| **Time in range Endpoint, % Six months, favor Glargine U-300 Bed** | | | | | | | | | | | |
| 1 | randomised trials | not serious^a^ | not serious | not serious | very serious^f^ | none | 23 | 23 | - | MD **2.2 lower**  (10.8 lower to 6.4 higher) | ⨁⨁◯◯  Low |
| **Time in range change, % Six months, favor NPH Bed** | | | | | | | | | | | |
| 1 | randomised trials | not serious^a^ | not serious | not serious | very serious^f^ | none | 23 | 23 | - | MD **0.5 higher**  (7.13 lower to 8.13 higher) | ⨁⨁◯◯  Low |

**CI:** confidence interval; **MD:** mean difference; **RR:** risk ratio.

**Explanations**

a. Downgraded by 0.5 level due to ROB: there are some concerning risks of biases in 2 domains.

b. Downgraded by two levels due to imprecision: wide confidence interval.

c. Downgraded by two levels due to imprecision: very small sample size (n = 46).

d. Downgraded by 2 levels due to ROB and imprecision: downgraded by 0.5 due to ROB: there are some concerning risks of biases in 2 domains,downgraded by 1.5 due to imprecision: wide confidence interval and low event rate.

e. Downgraded by 2 levels due to imprecision: confidence interval goes through 2%/3%/5%.

f. Downgraded by 2 levels due to ROB and imprecision: downgraded by 0.5 due to ROB: there are some concerning risks of biases in 2 domains,downgraded by 1.5 due to imprecision: confidence interval go through 0 and low sample size.

### Glargine U-100 vs. Detemir

| **Certainty assessment** | | | | | | | **№ of patients** | | **Effect** | | **Certainty** |
| --- | --- | --- | --- | --- | --- | --- | --- | --- | --- | --- | --- |
| **№ of studies** | **Study design** | **Risk of bias** | **Inconsistency** | **Indirectness** | **Imprecision** | **Other considerations** | **Glargine U-100 Bedtime** | **Detemir Bedtime** | **Relative**  **(95% CI)** | **Absolute**  **(95% CI)** |
| **HbA1c＜7.0 % Twelve months** | | | | | | | | | | | |
| 1 | randomised trials | not serious^a^ | not serious | not serious | serious^b^ | none | 135/259 (52.1%) | 129/248 (52.0%) | **RR 1.00**  (0.85 to 1.18) | **0 fewer per 1,000**  (from 78 fewer to 94 more) | ⨁⨁⨁◯  Moderate |
| **HbA1c Endpoint,% Twelve months, favor Glargine U-100 Bed** | | | | | | | | | | | |
| 1 | randomised trials | not serious^a^ | not serious | not serious | serious^c^ | none | 275 | 268 | - | MD **0.04 lower**  (0.26 lower to 0.18 higher) | ⨁⨁⨁◯  Moderate |
| **Hypoglycemia<3.0 Twelve months, favor Detemir Bed** | | | | | | | | | | | |
| 1 | randomised trials | not serious^a^ | not serious | not serious | very serious^d^ | none | 151/291 (51.9%) | 135/291 (46.4%) | **RR 1.12**  (0.95 to 1.32) | **56 more per 1,000**  (from 23 fewer to 148 more) | ⨁⨁◯◯  Low |
| **Severe Hypoglycemia Twelve months, favor Detemir Bed** | | | | | | | | | | | |
| 1 | randomised trials | not serious^a^ | not serious | not serious | very serious^e^ | none | 3/291 (1.0%) | 2/291 (0.7%) | **RR 1.50**  (0.25 to 8.91) | **3 more per 1,000**  (from 5 fewer to 54 more) | ⨁⨁◯◯  Low |
| **FPG≤6.0 Twelve months, favor Glargine U-100 Bed** | | | | | | | | | | | |
| 1 | randomised trials | not serious^a^ | not serious | not serious | very serious^d^ | none | 68/272 (25.0%) | 54/268 (20.1%) | **RR 1.24**  (0.91 to 1.70) | **48 more per 1,000**  (from 18 fewer to 141 more) | ⨁⨁◯◯  Low |
| **FPG Endpoint,mmol/L Twelve months, favor Glargine U-100 Bed** | | | | | | | | | | | |
| 1 | randomised trials | not serious^a^ | not serious | not serious | serious^c^ | none | 272 | 268 | - | MD **0.16 lower**  (0.74 lower to 0.42 higher) | ⨁⨁⨁◯  Moderate |
| **Nocturnal hypoglycemia<3.9 Twelve months, favor Glargine U-100 Bed** | | | | | | | | | | | |
| 1 | randomised trials | not serious^a^ | not serious | not serious | serious^c^ | none | 93/291 (32.0%) | 95/291 (32.6%) | **RR 0.98**  (0.77 to 1.24) | **7 fewer per 1,000**  (from 75 fewer to 78 more) | ⨁⨁⨁◯  Moderate |
| **Weight change,kg Six months, favor Detemir Bed** | | | | | | | | | | | |
| 2 | randomised trials | not serious^a^ | serious^f^ | not serious | very serious^e^ | none | 247 | 242 | - | MD **2.29 higher**  (0.27 higher to 4.31 higher) | ⨁◯◯◯  Very Low |
| **Weight change,kg Twelve months, favor Detemir Bed (Glargine U-100 Bed vs. Detemir Bed: 3.9 vs. 3)** | | | | | | | | | | | |
| 1 | randomised trials | not serious^a^ | not serious | not serious | very serious^g^ | none | 252 | 230 | - | MD **0.9 higher**  (0.21 lower to 2.01 higher) | ⨁⨁◯◯  Low |
| **HbA1c＜7.0 % Six month,favour Glargine U-100 Bed** | | | | | | | | | | | |
| 1 | randomised trials | not serious | not serious | not serious | serious^i^ | none | 107/204 (52.5%) | 80/209 (38.3%) | **RR 1.37**  (1.10 to 1.70) | **142 more per 1,000**  (from 38 more to 268 more) | ⨁⨁⨁◯  Moderate |
| **HbA1c Endpoint , % Six mon, favor Glargine U-100 Bed** | | | | | | | | | | | |
| 1 | randomised trials | not serious | not serious | not serious | serious^i^ | none | 227 | 226 | **-** | MD **0.35 lower**  (0.5 lower to 0.2 lower) | ⨁⨁⨁◯  Moderate |
| **HbA1c Change,% Six mon, favor Glargine U-100 Bed** | | | | | | | | | | | |
| 1 | randomised trials | not serious | not serious | not serious | serious^i^ | none | 227 | 226 | **-** | MD **0.26 lower**  (0.42 lower to 0.1 lower) | ⨁⨁⨁◯  Moderate |
| **Hypoglycemia < 3.0 mmol/L, Six month, favor Detemir Bed** | | | | | | | | | | | |
| 1 | randomised trials | not serious | not serious | not serious | serious^i^ | none | 72/227 (31.7%) | 54/226 (23.9%) | **RR 1.33**  (0.98 to 1.79) | **79 more per 1,000**  (from 5 fewer to 189 more) | ⨁⨁⨁◯  Moderate |
| **Severe hypoglycemia, Six month, favor Detemir Bed** | | | | | | | | | | | |
| 1 | randomised trials | not serious | not serious | not serious | very serious^e^ | none | 2/227 (0.9%) | 0/226 (0.0%) | **RR 4.98**  (0.24 to 103.11) | **0 fewer per 1,000**  (from 0 fewer to 0 fewer) | ⨁⨁◯◯  Low |
| **FPG Endpoint,mmol/L Six mon, favor Glargine U-100 Bed** | | | | | | | | | | | |
| 1 | randomised trials | not serious | not serious | not serious | serious^i^ | none | 227 | 226 | **-** | MD **0.13 lower**  (0.52 lower to 0.26 higher) | ⨁⨁⨁◯  Moderate |
| **FPG Change,mmol/L Six mon, favor Detemir Bed** | | | | | | | | | | | |
| 1 | randomised trials | not serious | not serious | not serious | serious^i^ | none | 227 | 226 | **-** | MD **0.08 higher**  (0.43 lower to 0.59 higher) | ⨁⨁⨁◯  Moderate |
| **Nocturnal hypoglycemia < 3.1 mmom/l, Six month, favor Glargine U-100 Bed** | | | | | | | | | | | |
| 1 | randomised trials | not serious | not serious | not serious | very serious^e^ | none | 19/227 (8.4%) | 29/226 (12.8%) | **RR 0.65**  (0.38 to 1.13) | **45 fewer per 1,000**  (from 80 fewer to 17 more) | ⨁⨁◯◯  Low |

**CI:** confidence interval; **MD:** mean difference; **RR:** risk ratio.

**Explanations**

a. Downgraded by 0.5 level due to ROB: there are some concerning risks of biases in 2 domains.

b. Downgraded by 1.5 levels due to imprecision: wide confidence interval and low event rate.

c. Downgraded by 1 level due to ROB and imprecision: downgraded by 0.5 due to ROB: there are some concerning risks of biases in 2 domains, downgraded by 0.5 level due to imprecision: low sample size (n = 543).

d. Downgraded by 2 levels due to ROB and imprecision: downgraded by 0.5 due to ROB: there are some concerning risks of biases in 2 domains,downgraded by 1.5 due to imprecision: wide confidence interval and low event rate.

e. Downgraded by two levels due to imprecision: wide confidence interval.

f. Downgraded by 1 level due to ROB and imprecision: downgraded by 0.5 due to ROB: there are some concern risk of bias in 2 domains, downgraded by 0.5 level due to heterogeneity. I^2^=71%.

g. Downgraded by 2 levels due to imprecision: confidence interval goes through 2%/3%/5%.

h. Downgraded by 1 level due to ROB and imprecision: downgraded by 0.5 due to ROB: there are some concerning risks of biases in 2 domains, downgraded by 0.5 due to imprecision: low event rate.

i. Downgraded by 1 level due to imprecision: wide confidence interval.

#### (5.1) Glargine U-100 NR vs. Detemir NR

| **Certainty assessment** | | | | | | | **№ of patients** | | **Effect** | | **Certainty** |
| --- | --- | --- | --- | --- | --- | --- | --- | --- | --- | --- | --- |
| **№ of studies** | **Study design** | **Risk of bias** | **Inconsistency** | **Indirectness** | **Imprecision** | **Other considerations** | **Glargine U-100 NR** | **Detemir NR** | **Relative**  **(95% CI)** | **Absolute**  **(95% CI)** |
| **HbA1c Change,% Three months, favor Glargine U-100 NR** | | | | | | | | | | | |
| 1 | randomised trials | not serious^a^ | not serious | not serious | very serious^b^ | none | 20 | 22 | - | MD **0.42 lower**  (1.11 lower to 0.27 higher) | ⨁⨁◯◯  Low |
| **FPG Endpoint,mmol/L Three months, favor Detemir NR** | | | | | | | | | | | |
| 1 | randomised trials | not serious^a^ | not serious | not serious | very serious^c^ | none | 20 | 22 | - | MD **0.1 higher**  (1.18 lower to 1.38 higher) | ⨁⨁◯◯  Low |
| **Weight change,kg Three months, favor Glargine U-100 NR (Glargine U-100 NR vs. Detemir NR: 0 vs. 0.74)** | | | | | | | | | | | |
| 1 | randomised trials | not serious^a^ | not serious | not serious | very serious^d^ | none | 20 | 22 | - | MD **0.74 lower**  (2.72 lower to 1.24 higher) | ⨁⨁◯◯  Low |

**CI:** confidence interval; **MD:** mean difference; **RR:** risk ratio.

**Explanations**

a. Downgraded by 0.5 level due to ROB: there are some concerning risks of biases in 4 domains.

b. Downgraded by two levels due to imprecision: small sample size (n = 42).

c. Downgraded by two levels due to imprecision: confidence interval go through 0.5 and 1.

d. Downgraded by two levels due to imprecision: confidence interval go through 2%/3% and 5%.

### Glargine U-100 vs. NPH

| **Certainty assessment** | | | | | | | **№ of patients** | | **Effect** | | **Certainty** |
| --- | --- | --- | --- | --- | --- | --- | --- | --- | --- | --- | --- |
| **№ of studies** | **Study design** | **Risk of bias** | **Inconsistency** | **Indirectness** | **Imprecision** | **Other considerations** | **Glargine U-100 Bedtime** | **NPH Bedtime** | **Relative**  **(95% CI)** | **Absolute**  **(95% CI)** |
| **HbA1c＜7.0 % Six months, favor Glargine U-100 Bed** | | | | | | | | | | | |
| 7 | randomised trials | not serious^a^ | not serious | not serious | serious^b^ | none | 766/1599 (47.9%) | 731/1634 (44.7%) | **RR 1.07**  (0.99 to 1.14) | **31 more per 1,000**  (from 4 fewer to 63 more) | ⨁⨁⨁◯  Moderate |
| **HbA1c Endpoint,% Three months, favor Glargine U-100 Bed** | | | | | | | | | | | |
| 3 | randomised trials | not serious^a^ | not serious | not serious | serious^c^ | none | 64 | 66 | - | MD **0.09 lower**  (0.37 lower to 0.2 higher) | ⨁⨁⨁◯  Moderate |
| **HbA1c Endpoint,% Six months, favor Glargine U-100 Bed** | | | | | | | | | | | |
| 8 | randomised trials | not serious^a^ | not serious^d^ | not serious | not serious | none | 1650 | 1665 | - | MD **0.11 lower**  (0.21 lower to 0.01 lower) | ⨁⨁⨁⨁  High |
| **HbA1c Endpoint,% Twelve months, favor NPH Bed** | | | | | | | | | | | |
| 1 | randomised trials | not serious^a^ | not serious | not serious | not serious | none | 214 | 208 | - | MD **0.1 higher**  (0.08 higher to 0.12 higher) | ⨁⨁⨁⨁  High |
| **Hypoglycemia<3.0 Three months, favor Glargine U-100 Bed** | | | | | | | | | | | |
| 2 | randomised trials | not serious | not serious | not serious | very serious^e^ | none | 28/75 (37.3%) | 25/63 (39.7%) | **RR 0.87**  (0.37 to 2.02) | **52 fewer per 1,000**  (from 250 fewer to 405 more) | ⨁⨁◯◯  Low |
| **Hypoglycemia<3.0 Six months, favor NPH Bed** | | | | | | | | | | | |
| 1 | randomised trials | not serious^f^ | not serious | not serious | very serious^g^ | none | 32/175 (18.3%) | 25/165 (15.2%) | **RR 1.21**  (0.75 to 1.95) | **32 more per 1,000**  (from 38 fewer to 144 more) | ⨁⨁◯◯  Low |
| **Severe Hypoglycemia Six months, favor Glargine U-100 Bed** | | | | | | | | | | | |
| 6 | randomised trials | not serious^a^ | not serious | not serious | very serious^e^ | none | 22/1270 (1.7%) | 25/1299 (1.9%) | **RR 0.92**  (0.51 to 1.64) | **2 fewer per 1,000**  (from 9 fewer to 12 more) | ⨁⨁◯◯  Low |
| **FPG<5.6 Six months, favor Glargine U-100 Bed** | | | | | | | | | | | |
| 2 | randomised trials | not serious^a^ | not serious | not serious | not serious^h^ | none | 229/595 (38.5%) | 232/635 (36.5%) | **RR 1.05**  (0.91 to 1.22) | **18 more per 1,000**  (from 33 fewer to 80 more) | ⨁⨁⨁⨁  High |
| **FPG≤6.7 Six months, favor Glargine U-100 Bed** | | | | | | | | | | | |
| 1 | randomised trials | not serious^f^ | not serious | not serious | serious^i^ | none | 137/220 (62.3%) | 131/223 (58.7%) | **RR 1.06**  (0.91 to 1.23) | **35 more per 1,000**  (from 53 fewer to 135 more) | ⨁⨁⨁◯  Moderate |
| **FPG Endpoint,mmol/L Three months, favor NPH Bed** | | | | | | | | | | | |
| 1 | randomised trials | not serious^f^ | not serious | not serious | very serious^j^ | none | 20 | 22 | - | MD **0.95 higher**  (0.48 lower to 2.38 higher) | ⨁⨁◯◯  Low |
| **FPG Endpoint,mmol/L Six months, favor Glargine U-100 Bed** | | | | | | | | | | | |
| 7 | randomised trials | not serious^a^ | not serious | not serious | not serious | none | 1580 | 1616 | - | MD **0.14 lower**  (0.26 lower to 0.02 lower) | ⨁⨁⨁⨁  High |
| **FPG Endpoint,mmol/L Twelve months, favor NPH Bed** | | | | | | | | | | | |
| 3 | randomised trials | not serious^a^ | serious^k^ | not serious | not serious | none | 491 | 438 | - | MD **0.2 higher**  (0.01 higher to 0.39 higher) | ⨁⨁⨁◯  Moderate |
| **Nocturnal hypoglycemia<3.9 Six months, favor Glargine U-100 Bed** | | | | | | | | | | | |
| 2 | randomised trials | not serious^a^ | not serious | not serious | very serious^g^ | none | 91/458 (19.9%) | 164/482 (34.0%) | **RR 0.58**  (0.47 to 0.73) | **143 fewer per 1,000**  (from 180 fewer to 92 fewer) | ⨁⨁◯◯  Low |
| **Nocturnal hypoglycemia<3.0 Twelve months, favor Glargine U-100 Bed** | | | | | | | | | | | |
| 2 | randomised trials | not serious^a^ | not serious | not serious | very serious^c^ | none | 44/436 (10.1%) | 99/412 (24.0%) | **RR 0.42**  (0.30 to 0.58) | **139 fewer per 1,000**  (from 168 fewer to 101 fewer) | ⨁⨁◯◯  Low |
| **Weight change,kg Six months, favor NPH Bed (1.Fritsche 2002-Glargine U-100 Bed vs. NPH Bed: 3.7 vs. 2.9; 2.Hermanns 2015-Glargine U-100 Bed vs. NPH Bed: 0.4 vs. 0.51; 3.Riddle 2003-Glargine U-100 Bed vs. NPH Bed: 3 vs. 2.8; 4.Hsia 2011-Glargine U-100 Bed vs. NPH Bed: 1.7 vs. -0.2)** | | | | | | | | | | | |
| 4 | randomised trials | not serious^a^ | not serious | not serious | very serious^l^ | none | 799 | 816 | - | MD **0.33 higher**  (0.11 lower to 0.77 higher) | ⨁⨁◯◯  Low |
| **Weight change,kg Twelve months, favor NPH Bed (Glargine U-100 Bed vs. NPH Bed: 2.57 vs. 2.34)** | | | | | | | | | | | |
| 1 | randomised trials | not serious^f^ | not serious | not serious | very serious^l^ | none | 214 | 208 | - | MD **0.23 higher**  (0.19 higher to 0.27 higher) | ⨁⨁◯◯  Low |
| **Weight change,kg Nine months, favor Glargine U-100 Bed (Glargine U-100 Bed vs. NPH Bed: 2.6 vs. 3.5; Home 2015-Glargine U-100 Bed vs. NPH Bed: 2.6 vs. 3.5)** | | | | | | | | | | | |
| 2 | randomised trials | not serious^f^ | not serious | not serious | very serious^l^ | none | 413 | 398 | - | MD **0.36 lower**  (1.45 lower to 0.73 higher) | ⨁⨁◯◯  Low |

**CI:** confidence interval; **MD:** mean difference; **RR:** risk ratio.

**Explanations**

a. Downgraded by 0.5 level due to ROB: there are some concerning risks of biases in 3 domains.

b. Downgraded by one level due to imprecision: confidence interval go through 1.

c. Downgraded by 1 level due to ROB and imprecision: downgraded by 0.5 due to ROB: there are some concerning risks of biases in 2 domains,downgraded by 0.5 due to imprecision: low sample size (n = 130).

d. Downgraded by 0.5 level due to heterogeneity. I^2^=60%-80%.

e. Downgraded by two levels due to imprecision: wide confidence interval.

f. Downgraded by 0.5 level due to ROB: there are some concerning risks of biases in 2 domains.

g. Downgraded by 2 levels due to ROB and imprecision: downgraded by 0.5 due to ROB: there are some concerning risks of biases in 2 domains,downgraded by 1.5 due to imprecision: wide confidence interval and low event rate.

h. Downgraded by 0.5 level due to imprecision: low event rate.

i. Downgraded by 1 level due to ROB and imprecision: downgraded by 0.5 due to ROB: there are some concerning risks of biases in 2 domains,downgraded by 0.5 due to imprecision: low event rate.

j. Downgraded by2 levels due to imprecision: confidence interval go through 0.5/1/1.5.

k. Downgraded by one level due to heterogeneity. I^2^=98%.

l. Downgraded by 2 levels due to imprecision: confidence interval go through 2%/3%/5%.

### Detemir vs. NPH

| **Certainty assessment** | | | | | | | **№ of patients** | | **Effect** | | **Certainty** |
| --- | --- | --- | --- | --- | --- | --- | --- | --- | --- | --- | --- |
| **№ of studies** | **Study design** | **Risk of bias** | **Inconsistency** | **Indirectness** | **Imprecision** | **Other considerations** | **Detemir Bedtime** | **NPH Bedtime** | **Relative**  **(95% CI)** | **Absolute**  **(95% CI)** |
| **HbA1c Change,% Six months, favor NPH Bed** | | | | | | | | | | | |
| 1 | randomised trials | not serious^a^ | not serious | not serious | not serious | none | 169 | 164 | - | MD **0.26 higher**  (0.04 higher to 0.48 higher) | ⨁⨁⨁⨁  High |
| **Hypoglycemia<3.0 Six months, favor Detemir Bed** | | | | | | | | | | | |
| 1 | randomised trials | not serious^a^ | not serious | not serious | very serious^b^ | none | 27/169 (16.0%) | 53/164 (32.3%) | **RR 0.49**  (0.33 to 0.75) | **165 fewer per 1,000**  (from 217 fewer to 81 fewer) | ⨁⨁◯◯  Low |
| **Severe Hypoglycemia Six months** | | | | | | | | | | | |
| 1 | randomised trials | not serious^a^ | not serious | not serious | very serious^c^ | none | 2/169 (1.2%) | 0/164 (0.0%) | **RR 4.85**  (0.23 to 100.32) | **0 fewer per 1,000**  (from 0 fewer to 0 fewer) | ⨁⨁◯◯  Low |
| **FPG Change,mmol/L Six months, favor Detemir Bed** | | | | | | | | | | | |
| 1 | randomised trials | not serious^a^ | not serious | not serious | very serious^d^ | none | 169 | 164 | - | MD **0.03 lower**  (0.71 lower to 0.65 higher) | ⨁⨁◯◯  Low |
| **Nocturnal hypoglycemia<3.0 Six months, favor Detemir Bed** | | | | | | | | | | | |
| 1 | randomised trials | not serious^a^ | not serious | not serious | very serious^e^ | none | 8/169 (4.7%) | 22/164 (13.4%) | **RR 0.35**  (0.16 to 0.77) | **87 fewer per 1,000**  (from 113 fewer to 31 fewer) | ⨁⨁◯◯  Low |
| **Weight change,kg Six months, favor Detemir Bed (1.Davies 2008-Detemir Bed vs. NPH Bed: 1.2 vs. 2.8; 2.Tsimikas 2006-Detemir Bed vs. NPH Bed: 0.7 vs. 1.6)** | | | | | | | | | | | |
| 1 | randomised trials | not serious^f^ | not serious | not serious | not serious | none | 169 | 164 | - | MD **0.9 lower**  (1.62 lower to 0.18 lower) | ⨁⨁⨁⨁  High |

**CI:** confidence interval; **MD:** mean difference; **RR:** risk ratio.

**Explanations**

a. Downgraded by 0.5 level due to ROB: there are some concerning risks of biases in 2 domains.

b. Downgraded by 2 levels due to ROB and imprecision: downgraded by 0.5 due to ROB: there are some concerning risks of biases in 2 domains,downgraded by 1.5 due to imprecision: wide confidence interval and low event rate.

c. Downgraded by two levels due to imprecision: wide confidence interval.

d. Downgraded by 2 levels due to ROB and imprecision: downgraded by 0.5 due to ROB: there are some concerning risks of biases in 2 domains,downgraded by 1.5 due to imprecision: confidence interval go through 0.5 and low sample size (n = 333).

e. Downgraded by two levels due to imprecision: wide confidence interval.

f. Downgraded by 0.5 level due to ROB: there are some concerning risks of biases in 4 domains.

## **2. One insulin morning vs. another insulin morning injection**

### (1) Degludec U-100 Morning vs. Glarginge U-100 Morning time

| **Certainty assessment** | | | | | | | **№ of patients** | | **Effect** | | **Certainty** |
| --- | --- | --- | --- | --- | --- | --- | --- | --- | --- | --- | --- |
| **№ of studies** | **Study design** | **Risk of bias** | **Inconsistency** | **Indirectness** | **Imprecision** | **Other considerations** | **Degludec U-100 Morning time** | **Glarginge U-100 Morning time** | **Relative**  **(95% CI)** | **Absolute**  **(95% CI)** |
| **HbA1c Endpoint,% Six months, favor Glarginge U-100 Morning** | | | | | | | | | | | |
| 1 | randomised trials | not serious^a^ | not serious | not serious | very serious^b^ | none | 32 | 12 | - | MD **0.2 higher**  (0.35 lower to 0.75 higher) | ⨁⨁◯◯  Low |
| **FPG Change,mmol/L Six months, favor Degludec U-100 Morning** | | | | | | | | | | | |
| 1 | randomised trials | not serious^a^ | not serious | not serious | very serious^b^ | none | 32 | 12 | - | MD **1.97 lower**  (2.66 lower to 1.28 lower) | ⨁⨁◯◯  Low |

**CI:** confidence interval; **MD:** mean difference; **RR:** risk ratio.

**Explanations**

a. Downgraded by 0.5 level due to ROB: there are some concerning risks of biases in 2 domains

b. Downgraded by 2 levels due to imprecision: low sample size (n = 44).

### (2) Detemir Morning time vs. NPH Morning time

One RCT with 86 patients met our preplanned study section criteria [ NCT00506662]. The main evidence favored detemir morning time were total hypoglycaemic episodes (rate/week) at 7 months (mean ± SD), detemir morning time vs. NPH morning time: 0.079 ± 0.359 vs. 0.146 ± 0.743, minor hypoglycaemic episodes (rate/week), detemir Morning time vs. NPH morning time: 0 vs. 0.125 ± 0.733. The main evidence favored NPH morning time was no data.

## **3. One insulin morning vs. another insulin bedtime injection**

### (1) Detemir Morning time vs. NPH Bedtime

| **Certainty assessment** | | | | | | | **№ of patients** | | **Effect** | | **Certainty** |
| --- | --- | --- | --- | --- | --- | --- | --- | --- | --- | --- | --- |
| **№ of studies** | **Study design** | **Risk of bias** | **Inconsistency** | **Indirectness** | **Imprecision** | **Other considerations** | **Detemir Morning time** | **NPH Bedtime** | **Relative**  **(95% CI)** | **Absolute**  **(95% CI)** |
| **HbA1c Change,% Six months, favor NPH Bed** | | | | | | | | | | | |
| 1 | randomised trials | not serious^a^ | not serious | not serious | serious^b^ | none | 165 | 164 | - | MD **0.16 higher**  (0.07 lower to 0.39 higher) | ⨁⨁⨁◯  Moderate |
| **Hypoglycemia<3.0 Six months, favor Detemir Morning** | | | | | | | | | | | |
| 1 | randomised trials | not serious^a^ | not serious | not serious | very serious^c^ | none | 32/165 (19.4%) | 53/164 (32.3%) | **RR 0.60**  (0.41 to 0.88) | **129 fewer per 1,000**  (from 191 fewer to 39 fewer) | ⨁⨁◯◯  Low |
| **Severe Hypoglycemia Six months** | | | | | | | | | | | |
| 1 | randomised trials | not serious^a^ | not serious | not serious |  | none | 0/165 (0.0%) | 0/164 (0.0%) | not estimable |  | - |
| **Nocturnal hypoglycemia<3.0 Six months, favor Detemir Morning** | | | | | | | | | | | |
| 1 | randomised trials | not serious^a^ | not serious | not serious | very serious^d^ | none | 4/165 (2.4%) | 22/164 (13.4%) | **RR 0.18**  (0.06 to 0.51) | **110 fewer per 1,000**  (from 126 fewer to 66 fewer) | ⨁⨁◯◯  Low |
| **FPG Change,mmol/L Six months, favor NPH Bed** | | | | | | | | | | | |
| 1 | randomised trials | not serious^a^ | not serious | not serious | very serious^e^ | none | 165 | 164 | - | MD **0.86 higher**  (0.14 higher to 1.58 higher) | ⨁⨁◯◯  Low |
| **Weight change,kg Six months, favor Detemir Morning (Detemir Morning vs. NPH Bed: 1.2 vs. 1.6)** | | | | | | | | | | | |
| 1 | randomised trials | not serious^a^ | not serious | not serious | very serious^f^ | none | 165 | 164 | - | MD **0.4 lower**  (1.12 lower to 0.32 higher) | ⨁⨁◯◯  Low |

**CI:** confidence interval; **MD:** mean difference; **RR:** risk ratio.

**Explanations**

a. Downgraded by 0.5 level due to ROB: there are some concerning risks of biases in 2 domains.

b. Downgraded by 1 level due to ROB and imprecision: downgraded by 0.5 due to ROB: there are some concerning risks of biases in 2 domains,downgraded by 0.5 due to imprecision: low sample size (n = 329).

c. Downgraded by two levels due to imprecision: wide confidence interval.

d. Downgraded by 2 levels due to ROB and imprecision: downgraded by 0.5 due to ROB: there are some concerning risks of biases in 2 domains,downgraded by 1.5 due to imprecision: wide confidence interval and low event rate.

e. Downgraded by 2 levels due to imprecision: confidence interval go through 0.5/1/1.5.

f. Downgraded by 2 levels due to imprecision: confidence interval go through 2%/3%/5%.

### (2) Glargine U-100 Morning time vs. NPH Bedtime

| **Certainty assessment** | | | | | | | **№ of patients** | | **Effect** | | **Certainty** |
| --- | --- | --- | --- | --- | --- | --- | --- | --- | --- | --- | --- |
| **№ of studies** | **Study design** | **Risk of bias** | **Inconsistency** | **Indirectness** | **Imprecision** | **Other considerations** | **Glargine U-100 Morning time** | **NPH Bedtime** | **Relative**  **(95% CI)** | **Absolute**  **(95% CI)** |
| **HbA1c＜7.0 % Six months, favor Glargine U-100 Morning** | | | | | | | | | | | |
| 2 | randomised trials | not serious^a^ | not serious | not serious | very serious^b^ | none | 109/261 (41.8%) | 81/262 (30.9%) | **RR 1.34**  (1.07 to 1.69) | **105 more per 1,000**  (from 22 more to 213 more) | ⨁⨁◯◯  Low |
| **HbA1c Change,% Six months, favor Glargine U-100 Morning** | | | | | | | | | | | |
| 2 | randomised trials | not serious^a^ | not serious | not serious | serious^c^ | none | 261 | 262 | - | MD **0.47 lower**  (1.18 lower to 0.23 higher) | ⨁⨁⨁◯  Moderate |
| **Severe Hypoglycemia Six months, favor Glargine U-100 Morning** | | | | | | | | | | | |
| 2 | randomised trials | not serious^a^ | not serious | not serious | very serious^d^ | none | 5/261 (1.9%) | 6/262 (2.3%) | **RR 0.82**  (0.25 to 2.65) | **4 fewer per 1,000**  (from 17 fewer to 38 more) | ⨁⨁◯◯  Low |
| **FPG Endpoint,mmol/L Six months, favor NPH Bed** | | | | | | | | | | | |
| 2 | randomised trials | not serious^a^ | not serious | not serious | serious^c^ | none | 261 | 262 | - | MD **0.1 higher**  (0.2 lower to 0.41 higher) | ⨁⨁⨁◯  Moderate |
| **Nocturnal hypoglycemia<3.9 Six months, favor Glargine U-100 Morning** | | | | | | | | | | | |
| 1 | randomised trials | not serious^a^ | not serious | not serious | very serious^e^ | none | 39/236 (16.5%) | 89/232 (38.4%) | **RR 0.43**  (0.31 to 0.60) | **219 fewer per 1,000**  (from 265 fewer to 153 fewer) | ⨁⨁◯◯  Low |
| **Weight change,kg Six months, favor NPH Bed (Glargine U-100 Morning vs. NPH Bed: 3.9 vs. 2.9)** | | | | | | | | | | | |
| 2 | randomised trials | not serious^a^ | not serious^f^ | not serious | very serious^g^ | none | 261 | 262 | - | MD **1.93 higher**  (0.28 lower to 4.14 higher) | ⨁⨁◯◯  Low |
| **Weight endpoint, kg Six months, favor NPH Bed (Glargine U-100 Morning vs. NPH Bed: 85.8 vs. 82.4)** | | | | | | | | | | | |
| 1 | randomised trials | not serious^h^ | not serious | not serious | very serious^g^ | none | 25 | 30 | - | MD **3.4 higher**  (5.44 lower to 12.2 higher) | ⨁⨁◯◯  Low |
| **FPG change, mmol/L Six months, favor Glargine U-100 Morning** | | | | | | | | | | | |
| 1 | randomised trials | not serious^h^ | not serious | not serious | very serious^i^ | none | 25 | 30 | - | MD **0.05 lower**  (1.1 lower to 1 higher) | ⨁⨁◯◯  Low |

**CI:** confidence interval; **MD:** mean difference; **RR:** risk ratio.

**Explanations**

a. Downgraded by 0.5 level due to ROB: there are some concerning risks of biases in 3 domains.

b. Downgraded by 2 levels due to imprecision: confidence interval goes through 0.4 and 1.

c. Downgraded by 1 level due to ROB and imprecision: downgraded by 0.5 due to ROB: there are some concerning risks of biases in 3 domains, downgraded by 0.5 due to imprecision: low sample size (n = 523).

d. Downgraded by two levels due to imprecision: wide confidence interval.

e. Downgraded by 2 levels due to ROB and imprecision: downgraded by 0.5 due to ROB: there are some concerning risks of biases in 3 domains, downgraded by 1.5 due to imprecision: wide confidence interval and low event rate.

f. Downgraded by 0.5 level due to heterogeneity. I^2^ = 60%-80%.

g. Downgraded by 2 levels due to imprecision: confidence interval goes through 2%/3%/5%.

h. Downgraded by 0.5 level due to ROB: there are high risk of bias in 1 domain but a low sample size.

i. Downgraded by 2 levels due to imprecision: confidence interval goes through 0.5 and low sample size(n = 55).

## **4. One insulin morning time vs. its bedtime injection**

### Detemir Morning time vs. Detemir Bedtime

| **Certainty assessment** | | | | | | | **№ of patients** | | **Effect** | | **Certainty** |
| --- | --- | --- | --- | --- | --- | --- | --- | --- | --- | --- | --- |
| **№ of studies** | **Study design** | **Risk of bias** | **Inconsistency** | **Indirectness** | **Imprecision** | **Other considerations** | **Detemir Morning time** | **Detemir Bedtime** | **Relative**  **(95% CI)** | **Absolute**  **(95% CI)** |
| **HbA1c Change,% Six months, favor Detemir Morning** | | | | | | | | | | | |
| 1 | randomised trials | not serious^a^ | not serious | not serious | serious^b^ | none | 165 | 169 | - | MD **0.1 lower**  (0.32 lower to 0.12 higher) | ⨁⨁⨁◯  Moderate |
| **Hypoglycemia<3.0 Six months, favor Detemir Bed** | | | | | | | | | | | |
| 1 | randomised trials | not serious^a^ | not serious | not serious | very serious^c^ | none | 32/165 (19.4%) | 27/169 (16.0%) | **RR 1.21**  (0.76 to 1.93) | **34 more per 1,000**  (from 38 fewer to 149 more) | ⨁⨁◯◯  Low |
| **Severe Hypoglycemia Six months, favor Detemir Morning** | | | | | | | | | | | |
| 1 | randomised trials | not serious^a^ | not serious | not serious | very serious^d^ | none | 0/165 (0.0%) | 2/169 (1.2%) | **RR 0.20**  (0.01 to 4.23) | **9 fewer per 1,000**  (from 12 fewer to 38 more) | ⨁⨁◯◯  Low |
| **FPG Change,mmol/L Six months, favor Detemir Bed** | | | | | | | | | | | |
| 1 | randomised trials | not serious^a^ | not serious | not serious | very serious^e^ | none | 165 | 169 | - | MD **0.89 higher**  (0.31 higher to 1.47 higher) | ⨁⨁◯◯  Low |
| **Nocturnal hypoglycemia<3.0 Six months, favor Detemir Morning** | | | | | | | | | | | |
| 1 | randomised trials | not serious^a^ | not serious | not serious | very serious^d^ | none | 4/165 (2.4%) | 8/169 (4.7%) | **RR 0.51**  (0.16 to 1.67) | **23 fewer per 1,000**  (from 40 fewer to 32 more) | ⨁⨁◯◯  Low |
| **Weight change,kg Six months, favor Detemir Bed (Detemir Morning vs. Detemir Bed: 1.2 vs. 0.7)** | | | | | | | | | | | |
| 1 | randomised trials | not serious^a^ | not serious | not serious | very serious^f^ | none | 165 | 169 | - | MD **0.5 higher**  (0.22 lower to 1.22 higher) | ⨁⨁◯◯  Low |

**CI:** confidence interval; **MD:** mean difference; **RR:** risk ratio.

**Explanations**

a. Downgraded by 0.5 level due to ROB: there are some concerning risks of biases in 2 domains.

b. Downgraded by 1 level due to ROB and imprecision: downgraded by 0.5 due to ROB: there are some concerning risks of biases in 2 domains,downgraded by 0.5 due to imprecision: low sample size(n = 334).

c. Downgraded by 2 levels due to ROB and imprecision: downgraded by 0.5 due to ROB: there are some concerning risks of biases in 2 domains,downgraded by 1.5 due to imprecision: wide confidence interval and low event rate.

d. Downgraded by two levels due to imprecision: wide confidence interval.

e. Downgraded by 2 levels due to imprecision: confidence interval go through 0.5 and 1.

f. Downgraded by 2 levels due to imprecision: confidence interval go through 2%/3%/5%.

### Glargine U-100 Morning time vs. Glargine U-100 Bedtime

| **Certainty assessment** | | | | | | | **№ of patients** | | **Effect** | | **Certainty** |
| --- | --- | --- | --- | --- | --- | --- | --- | --- | --- | --- | --- |
| **№ of studies** | **Study design** | **Risk of bias** | **Inconsistency** | **Indirectness** | **Imprecision** | **Other considerations** | **Glargine U-100 Morning time** | **Glargine U-100 Bedtime** | **Relative**  **(95% CI)** | **Absolute**  **(95% CI)** |
| **HbA1c < 7.0 % Six months, favor Glargine U-100 Morning** | | | | | | | | | | | |
| 2 | randomised trials | not serious^a^ | not serious | not serious | very serious^b^ | none | 109/261 (41.8%) | 82/257 (31.9%) | **RR 1.30**  (1.04 to 1.63) | **96 more per 1,000**  (from 13 more to 201 more) | ⨁⨁◯◯  Low |
| **HbA1c Change,% Six months, favor Glargine U-100 Morning** | | | | | | | | | | | |
| 2 | randomised trials | not serious^a^ | not serious | not serious | serious^c^ | none | 261 | 257 | - | MD **0.54 lower**  (1.16 lower to 0.09 higher) | ⨁⨁⨁◯  Moderate |
| **Severe Hypoglycemia Six months, favor Glargine U-100 Bed** | | | | | | | | | | | |
| 2 | randomised trials | not serious^a^ | not serious | not serious | very serious^d^ | none | 5/261 (1.9%) | 4/257 (1.6%) | **RR 1.20**  (0.33 to 4.42) | **3 more per 1,000**  (from 10 fewer to 53 more) | ⨁⨁◯◯  Low |
| **FPG Endpoint,mmol/L Six months, favor Glargine U-100 Morning** | | | | | | | | | | | |
| 2 | randomised trials | not serious^a^ | not serious^e^ | not serious | very serious^f^ | none | 261 | 257 | - | MD **0.1 lower**  (0.82 lower to 0.62 higher) | ⨁⨁◯◯  Low |
| **Nocturnal hypoglycemia<3.9 Six months, favor Glargine U-100 Morning** | | | | | | | | | | | |
| 1 | randomised trials | not serious^a^ | not serious | not serious | very serious^b^ | none | 39/236 (16.5%) | 52/227 (22.9%) | **RR 0.72**  (0.50 to 1.05) | **64 fewer per 1,000**  (from 115 fewer to 11 more) | ⨁⨁◯◯  Low |
| **Weight change,kg Six months, favor Glargine U-100 Bed (Fritsche 2003: Glargine U-100 Morning vs. Glargine U-100 Bed: 3.9 vs. 3.7; Hsia 2011: Glargine U-100 Morning vs. Glargine U-100 Bed: 3.1 vs. 1.7)** | | | | | | | | | | | |
| 2 | randomised trials | not serious^a^ | not serious | not serious | very serious^g^ | none | 261 | 257 | - | MD **0.33 higher**  (0.41 lower to 1.08 higher) | ⨁⨁◯◯  Low |

**CI:** confidence interval; **MD:** mean difference; **RR:** risk ratio.

**Explanations**

a. Downgraded by 0.5 level due to ROB: there are some concerning risks of biases in 3 domains.

b. Downgraded by 2 levels due to ROB and imprecision: downgraded by 0.5 due to ROB: there are some concerning risks of biases in 3 domains,downgraded by 1.5 due to imprecision: wide confidence interval and low event rate.

c. Downgraded by 1 level due to ROB and imprecision: downgraded by 0.5 due to ROB: there are some concerning risks of biases in 3 domains,downgraded by 0.5 due to imprecision: low sample size (n = 518).

d. Downgraded by two levels due to imprecision: wide confidence interval.

e. Downgraded by 0.5 level due to heterogeneity. I^2^ = 60%-80%.

f. Downgraded by 2 levels due to ROB and imprecision: downgraded by 0.5 due to ROB: there are some concerning risks of biases in 3 domains, downgraded by 1.5 due to imprecision: confidence interval go through 0.5 and low sample size (n = 518).

g. Downgraded by one level due to imprecision: confidence interval go through 2%/3% and 5%.

# Q2. What is the optimal starting dose (U/kg/day) for initiation of the five basal insulins and optimal time of injection (morning versus bedtime administration) to control glycemia with acceptable side effects for the target population?

## 1. Detemir once 0.12U/kg/day vs. Detemir twice 0.12U/kg/day

| **Certainty assessment** | | | | | | | **№ of patients** | | **Effect** | | **Certainty** |
| --- | --- | --- | --- | --- | --- | --- | --- | --- | --- | --- | --- |
| **№ of studies** | **Study design** | **Risk of bias** | **Inconsistency** | **Indirectness** | **Imprecision** | **Other considerations** | **Detemir once 0.12U/kg/day** | **Detemir twice 0.12U/kg/day** | **Relative**  **(95% CI)** | **Absolute**  **(95% CI)** |
| **HbA1c<7% , favor Detemir twice** | | | | | | | | | | | |
| 1 | randomised trials | not serious^a^ | not serious | not serious | very serious^b^ | none | 5/25 (20.0%) | 7/25 (28.0%) | **RR 0.71**  (0.26 to 1.95) | **81 fewer per 1,000**  (from 207 fewer to 266 more) | ⨁⨁◯◯  Low |
| **HbA1c Change，% favor Detemir once** | | | | | | | | | | | |
| 1 | randomised trials | not serious^a^ | not serious | not serious | very serious^b^ | none | 25 | 25 | - | MD **0.3 lower**  (1.26 lower to 0.66 higher) | ⨁⨁◯◯  Low |
| **HbA1c Endpoint,% favor Detemir twice** | | | | | | | | | | | |
| 1 | randomised trials | not serious^a^ | not serious | not serious | very serious^b^ | none | 25 | 25 | - | MD **0.2 higher**  (0.38 lower to 0.78 higher) | ⨁⨁◯◯  Low |
| **FPG Endpoint, mmol/L favor Detemir once** | | | | | | | | | | | |
| 1 | randomised trials | not serious^a^ | not serious | not serious | very serious^c^ | none | 25 | 25 | - | MD **0.04 lower**  (1.2 lower to 1.12 higher) | ⨁⨁◯◯  Low |
| **Hypoglycemia** | | | | | | | | | | | |
| 1 | randomised trials | not serious^a^ | not serious | not serious | very serious^d^ | none | 0/25 (0.0%) | 0/25 (0.0%) | **RR 0.07**  (0.00 to 1.11) | **0 fewer per 1,000**  (from 0 fewer to --) | ⨁⨁◯◯  Low |
| **Weight change,kg favor Detemir twice (once vs. twice: 0.4 vs. -0.1)** | | | | | | | | | | | |
| 1 | randomised trials | not serious^a^ | not serious | not serious | very serious^e^ | none | 25 | 25 | - | MD **0.5 higher**  (0.55 lower to 1.55 higher) | ⨁⨁◯◯  Low |
| **Weight Endpoint,kg favor Detemir once** | | | | | | | | | | | |
| 1 | randomised trials | not serious^a^ | not serious | not serious | very serious^e^ | none | 25 | 25 | - | MD **4.4 lower**  (12.72 lower to 3.92 higher) | ⨁⨁◯◯  Low |

**CI:** confidence interval; **MD:** mean difference; **RR:** risk ratio.

**Explanations**

a. Downgraded by 0.5 level due to ROB: there are some concerning risks of biases in 2 domains

b. Downgraded by two levels due to imprecision: sample size = 50 and confidence interval go through 0.4.

c. Downgraded by two levelsdue to imprecision: confidence interval go through 0.5 and 1.

d. Downgraded by two levels due to imprecision: very low event rate.

e. Downgraded by two levelsdue to imprecision: confidence interval go through 2%/3% and 5%.

## **2. Glargine U-100 0.2 U/kg vs. Glargine U-100 0.3 U/kg**

| **Certainty assessment** | | | | | | | **№ of patients** | | **Effect** | | **Certainty** |
| --- | --- | --- | --- | --- | --- | --- | --- | --- | --- | --- | --- |
| **№ of studies** | **Study design** | **Risk of bias** | **Inconsistency** | **Indirectness** | **Imprecision** | **Other considerations** | **Glargine U-100 0.2 U/kg** | **Glargine U-100 0.3 U/kg** | **Relative**  **(95% CI)** | **Absolute**  **(95% CI)** |
| **HbA1c<7% favor 0.3, but two groups are very similar** | | | | | | | | | | | |
| 1 | randomised trials | not serious^a^ | not serious | not serious | not serious | none | 172/429 (40.1%) | 178/437 (40.7%) | **RR 0.98**  (0.84 to 1.16) | **8 fewer per 1,000**  (from 65 fewer to 65 more) | ⨁⨁⨁⨁  High |
| **FPG<5.6 mmol/L favor 0.3, but two groups are very similar** | | | | | | | | | | | |
| 1 | randomised trials | serious^b^ | not serious | not serious | serious^c^ | none | 81/429 (18.9%) | 84/437 (19.2%) | **RR 0.98**  (0.75 to 1.29) | **4 fewer per 1,000**  (from 48 fewer to 56 more) | ⨁⨁◯◯  Low |
| **FPG<6.1 mmol/L favor 0.3** | | | | | | | | | | | |
| 1 | randomised trials | not serious^a^ | not serious | not serious | serious^c^ | none | 125/429 (29.1%) | 140/437 (32.0%) | **RR 0.91**  (0.74 to 1.11) | **29 fewer per 1,000**  (from 83 fewer to 35 more) | ⨁⨁⨁◯  Moderate |
| **FPG<7.0 mmol/L favor 0.3, but two groups are very similar** | | | | | | | | | | | |
| 1 | randomised trials | not serious^a^ | not serious | not serious | not serious | none | 230/429 (53.6%) | 242/437 (55.4%) | **RR 0.97**  (0.86 to 1.09) | **17 fewer per 1,000**  (from 78 fewer to 50 more) | ⨁⨁⨁⨁  High |
| **Hypoglycemia favor 0.2** | | | | | | | | | | | |
| 1 | randomised trials | serious^b^ | not serious | not serious | serious^c^ | none | 37/429 (8.6%) | 48/437 (11.0%) | **RR 0.79**  (0.52 to 1.18) | **23 fewer per 1,000**  (from 53 fewer to 20 more) | ⨁⨁◯◯  Low |
| **Severe Hypoglycemia** | | | | | | | | | | | |
| 1 | randomised trials | not serious^a^ | not serious | not serious | serious^d^ | none | 0/429 (0.0%) | 0/437 (0.0%) | **RR 0.01**  (0.00 to 0.17) | **0 fewer per 1,000**  (from 0 fewer to --) | ⨁⨁⨁◯  Moderate |
| **Nocturnal Hypoglycemia favor 0.2** | | | | | | | | | | | |
| 1 | randomised trials | not serious^a^ | not serious | not serious | very serious^e^ | none | 5/429 (1.2%) | 12/437 (2.7%) | **RR 0.42**  (0.15 to 1.19) | **16 fewer per 1,000**  (from 23 fewer to 5 more) | ⨁⨁◯◯  Low |
| **Weight change, kg favor 0.2 U/kg Group (group 0.2 vs. group 0.3: 0 vs. 0.4)** | | | | | | | | | | | |
| 1 | randomised trials | not serious^a^ | not serious | not serious | very serious^f^ | none | 429 | 437 | - | MD **0.4 lower**  (0.74 lower to 0.06 lower) | ⨁⨁◯◯  Low |
| **HbA1c Change, %, favor Glargine U-100 0.3 U/kg** | | | | | | | | | | | |
| 1 | randomised trials | not serious^g^ | not serious | not serious | very serious^f^ | none | 429 | 437 | - | MD **0.05 higher**  (0.08 lower to 0.18 higher) | ⨁⨁◯◯  Low |
| **FPG Change, mmol/L, favor Glargine U-100 0.2 U/kg** | | | | | | | | | | | |
| 1 | randomised trials | not serious^g^ | not serious | not serious | very serious^f^ | none | 429 | 437 | - | MD **0.02 lower**  (0.3 lower to 0.26 higher) | ⨁⨁◯◯  Low |

**CI:** confidence interval; **MD:** mean difference; **RR:** risk ratio.

**Explanations**

a. Downgraded by 0.5 level due to ROB: there are some concerning risks of biases in 3 domains

b. Downgraded by 1 level due to ROB and imprecision: downgraded by 0.5 due to ROB: there are some concerning risks of biases in 3 domains,downgraded by 0.5 due to imprecision: low event rate

c. Downgraded by one level due to imprecision: wide confidence interval and small sample size.

d. Downgraded by two levelsdue to imprecision: wide confidence interval and small sample size.

e. Downgraded by two levelsdue to imprecision: wide confidence interval.

f. Downgraded by two levelsdue to imprecision: confidence interval go through 2%/3% and 5%.

g. Downgraded by 0.5 level due to ROB: there are some concerning risks of biases in 2 domains.

# Q4. After initiation of any of the five basal insulins, what range of target FPG can lead to the ideal HbA1c level (i.e., < 7.0%) in the target population?

## Insulin Glargine U-100 3.9 < FBG ≤ 5.6 mmol/L vs. 3.9 < FBG ≤ 6.1 mmol/L

| **Certainty assessment** | | | | | | | **№ of patients** | | **Effect** | | **Certainty** |
| --- | --- | --- | --- | --- | --- | --- | --- | --- | --- | --- | --- |
| **№ of studies** | **Study design** | **Risk of bias** | **Inconsistency** | **Indirectness** | **Imprecision** | **Other considerations** | **Insulin Glargine U-100 3.9 < FBG ≤ 5.6 mmol/L** | **3.9 < FBG ≤ 6.1 mmol/L** | **Relative**  **(95% CI)** | **Absolute**  **(95% CI)** |
| **HbA1c < 7% Six months, favor 6.1 mmol/ L** | | | | | | | | | | | |
| 2 | randomised trials | not serious^a^ | not serious | not serious | very serious^b^ | none | 57/136 (41.9%) | 189/419 (45.1%) | **RR 0.87**  (0.46 to 1.65) | **59 fewer per 1,000**  (from 244 fewer to 293 more) | ⨁⨁◯◯  Low |
| **HbA1c change,% Six months, favor 6.1 mmol/ L** | | | | | | | | | | | |
| 2 | randomised trials | not serious^a^ | serious^c^ | not serious | very serious^d^ | none | 129 | 393 | - | MD **0.36 higher**  (0.46 lower to 1.18 higher) | ⨁◯◯◯  Very low |
| **Hypoglycemia any Six months,favor 6.1 mmol/ L** | | | | | | | | | | | |
| 1 | randomised trials | not serious^a^ | not serious | not serious | serious^e^ | none | 72/126 (57.1%) | 169/393 (43.0%) | **RR 1.33**  (1.10 to 1.61) | **142 more per 1,000**  (from 43 more to 262 more) | ⨁⨁⨁◯  Moderate |
| **Hypoglycaemia ≤3.9 mmol/L Six months, favor 6.1 mmol/L** | | | | | | | | | | | |
| 1 | randomised trials | not serious^a^ | not serious | not serious | serious^e^ | none | 49/126 (38.9%) | 108/393 (27.5%) | **RR 1.42**  (1.08 to 1.86) | **115 more per 1,000**  (from 22 more to 236 more) | ⨁⨁⨁◯  Moderate |
| **Hypoglycaemia ≤3.0 mmol/L Six months, favor 6.1 mmol/L** | | | | | | | | | | | |
| 1 | randomised trials | not serious^a^ | not serious | not serious | very serious^b^ | none | 6/126 (4.8%) | 8/393 (2.0%) | **RR 2.34**  (0.83 to 6.61) | **27 more per 1,000**  (from 3 fewer to 114 more) | ⨁⨁◯◯  Low |
| **Severe Hypoglycemia Six months** | | | | | | | | | | | |
| 1 | randomised trials | not serious^a^ | not serious | not serious | very serious^f^ | none | 0/126 (0.0%) | 1/393 (0.3%) | **RR 1.03**  (0.04 to 25.23) | **0 fewer per 1,000**  (from 2 fewer to 62 more) | ⨁⨁◯◯  Low |
| **Nocturnal Hypoglycemia ≤3.9 mmol/L Six months, favor 6.1 mmol/ L** | | | | | | | | | | | |
| 1 | randomised trials | not serious^a^ | not serious | not serious | very serious^f^ | none | 25/126 (19.8%) | 42/393 (10.7%) | **RR 1.86**  (1.18 to 2.92) | **92 more per 1,000**  (from 19 more to 205 more) | ⨁⨁◯◯  Low |

**CI:** confidence interval; **MD:** mean difference; **RR:** risk ratio.

**Explanations**

a. Downgraded by 0.5 level due to ROB: there is high risk of bias in one risk domains but sample size more than 50.

b. Downgraded by two levels due to imprecision: wide confidence interval.

c. Downgraded by one level due to heterogeneity. I^2^=93%.

d. Downgraded by two levelsdue to imprecision: confidence interval go through 0.4 and 1.

e. Downgraded by 1.5 levels due to imprecision: wide confidence interval and low event rate.

f. Downgraded by 2 levels due to imprecision: wide confidence interval and low event rate.

## Insulin Glargine U-100 3.9 < FBG ≤ 5.6 mmol/L compared to 3.9 < FBG ≤ 7.0 mmol/L

| **Certainty assessment** | | | | | | | **№ of patients** | | **Effect** | | **Certainty** |
| --- | --- | --- | --- | --- | --- | --- | --- | --- | --- | --- | --- |
| **№ of studies** | **Study design** | **Risk of bias** | **Inconsistency** | **Indirectness** | **Imprecision** | **Other considerations** | **Insulin Glargine U-100 3.9 < FBG ≤ 5.6 mmol/L** | **3.9 < FBG ≤ 7.0 mmol/L** | **Relative**  **(95% CI)** | **Absolute**  **(95% CI)** |
| **HbA1c < 7% Six months, favor 5.6 mmol/ L** | | | | | | | | | | | |
| 2 | randomised trials | not serious^a^ | not serious | not serious | serious^b^ | none | 57/136 (41.9%) | 155/430 (36.0%) | **RR 1.17**  (0.93 to 1.47) | **61 more per 1,000**  (from 25 fewer to 169 more) | ⨁⨁⨁◯  Moderate |
| **HbA1c change,% Six months, favor 7.0 mmol/ L** | | | | | | | | | | | |
| 2 | randomised trials | not serious^a^ | serious^c^ | not serious | serious^d^ | none | 129 | 412 | - | MD **0.08 higher**  (0.52 lower to 0.68 higher) | ⨁⨁◯◯  Low |
| **Hypoglycemia any Six months, favor 7.0 mmol/ L** | | | | | | | | | | | |
| 1 | randomised trials | not serious^a^ | not serious | not serious | not serious^e^ | none | 72/126 (57.1%) | 145/395 (36.7%) | **RR 1.56**  (1.28 to 1.90) | **206 more per 1,000**  (from 103 more to 330 more) | ⨁⨁⨁⨁  High |
| **Severe Hypoglycemia Six months** | | | | | | | | | | | |
| 1 | randomised trials | not serious^a^ | not serious | not serious | very serious^f^ | none | 0/126 (0.0%) | 1/395 (0.3%) | **RR 1.04**  (0.04 to 25.35) | **0 fewer per 1,000**  (from 2 fewer to 62 more) | ⨁⨁◯◯  Low |
| **Nocturnal Hypoglycemia ≤3.9 mmol/ Six months, favor 7.0 mmol/ L** | | | | | | | | | | | |
| 1 | randomised trials | not serious^a^ | not serious | not serious | serious^b^ | none | 25/126 (19.8%) | 34/395 (8.6%) | **RR 2.31**  (1.43 to 3.71) | **113 more per 1,000**  (from 37 more to 233 more) | ⨁⨁⨁◯  Moderate |
| **Hypoglycemia ≤3.9 mmol/L Six months, favor 7.0 mmol/ L** | | | | | | | | | | | |
| 1 | randomised trials | not serious^a^ | not serious | not serious | serious^b^ | none | 49/126 (38.9%) | 92/395 (23.3%) | **RR 1.67**  (1.26 to 2.22) | **156 more per 1,000**  (from 61 more to 284 more) | ⨁⨁⨁◯  Moderate |
| **Hypoglycemia <3.0 mmol/L Six months, favor 7.0 mmol/ L** | | | | | | | | | | | |
| 1 | randomised trials | not serious^a^ | not serious | not serious | very serious^f^ | none | 6/126 (4.8%) | 15/395 (3.8%) | **RR 1.25**  (0.50 to 3.16) | **9 more per 1,000**  (from 19 fewer to 82 more) | ⨁⨁◯◯  Low |

**CI:** confidence interval; **MD:** mean difference; **RR:** risk ratio.

**Explanations**

a. Downgraded by 0.5 level due to ROB: there is high risk of bias in one risk domains, but sample size more than 50.

b. Downgraded by 1.5 levels due to imprecision: wide confidence interval and low event rate.

c. Downgraded by one level due to heterogeneity. I^2^=91%.

d. Downgraded by 1.5 levels due to imprecision: confidence interval go through 0.4 and small sample size (n = 541).

e. Downgraded by 0.5 level due to imprecision: low event rate.

f. Downgraded by two levels due to imprecision: wide confidence interval.

## Insulin Glargine U-100 3.9 < FBG ≤ 6.1 mmol/L vs. 3.9 < FBG ≤ 7.0 mmol/L

| **Certainty assessment** | | | | | | | **№ of patients** | | **Effect** | | **Certainty** |
| --- | --- | --- | --- | --- | --- | --- | --- | --- | --- | --- | --- |
| **№ of studies** | **Study design** | **Risk of bias** | **Inconsistency** | **Indirectness** | **Imprecision** | **Other considerations** | **Insulin Glargine U-100 3.9 < FBG ≤ 6.1 mmol/L** | **3.9 < FBG ≤ 7.0 mmol/L** | **Relative**  **(95% CI)** | **Absolute**  **(95% CI)** |
| **HbA1c < 7% Six months, favor 6.1 mmol/ L** | | | | | | | | | | | |
| 2 | randomised trials | not serious^a^ | not serious | not serious | serious^b^ | none | 189/419 (45.1%) | 155/430 (36.0%) | **RR 1.24**  (1.05 to 1.45) | **87 more per 1,000**  (from 18 more to 162 more) | ⨁⨁⨁◯  Moderate |
| **HbA1c change,% Six months, favor 6.1 mmol/ L** | | | | | | | | | | | |
| 2 | randomised trials | not serious^a^ | not serious | not serious | not serious | none | 393 | 412 | - | MD **0.18 lower**  (0.29 lower to 0.07 lower) | ⨁⨁⨁⨁  High |
| **Hypoglycemia any Six months, favor 7.0 mmol/ L** | | | | | | | | | | | |
| 1 | randomised trials | not serious^a^ | not serious | not serious | serious^b^ | none | 169/393 (43.0%) | 145/395 (36.7%) | **RR 1.17**  (0.99 to 1.39) | **62 more per 1,000**  (from 4 fewer to 143 more) | ⨁⨁⨁◯  Moderate |
| **Severe Hypoglycemia Six months** | | | | | | | | | | | |
| 1 | randomised trials | not serious^a^ | not serious | not serious | very serious^c^ | none | 1/393 (0.3%) | 1/395 (0.3%) | **RR 1.01**  (0.06 to 16.01) | **0 fewer per 1,000**  (from 2 fewer to 38 more) | ⨁⨁◯◯  Low |
| **Nocturnal Hypoglycemia ≤3.9 mmol/L Six months, favor 7.0 mmol/ L** | | | | | | | | | | | |
| 1 | randomised trials | not serious^a^ | not serious | not serious | serious^b^ | none | 42/393 (10.7%) | 34/395 (8.6%) | **RR 1.24**  (0.81 to 1.91) | **21 more per 1,000**  (from 16 fewer to 78 more) | ⨁⨁⨁◯  Moderate |
| **Hypoglycemia<3.9 mmol/L Six months, favor 7.0 mmol/ L** | | | | | | | | | | | |
| 1 | randomised trials | not serious^a^ | not serious | not serious | serious^b^ | none | 108/393 (27.5%) | 92/395 (23.3%) | **RR 1.18**  (0.93 to 1.50) | **42 more per 1,000**  (from 16 fewer to 116 more) | ⨁⨁⨁◯  Moderate |
| **Hypoglycemia<3.0 mmol/L Six months, favor 6.1 mmol/ L** | | | | | | | | | | | |
| 1 | randomised trials | not serious^a^ | not serious | not serious | very serious^c^ | none | 8/393 (2.0%) | 15/395 (3.8%) | **RR 0.54**  (0.23 to 1.25) | **17 fewer per 1,000**  (from 29 fewer to 9 more) | ⨁⨁◯◯  Low |

**CI:** confidence interval; **MD:** mean difference; **RR:** risk ratio.

**Explanations**

a. Downgraded by 0.5 level due to ROB: there is high risk of bias in one risk domains but sample size more than 50.

b. Downgraded by 1.5 levels due to imprecision: wide confidence interval and low event rate.

c. Downgraded by two levels due to imprecision: wide confidence interval.

## Insulin Detemir 3.9 < FBG ≤ 5.0 mmol/L compared to 4.4 < FBG ≤ 6.1 mmol/L

| **Certainty assessment** | | | | | | | **№ of patients** | | **Effect** | | **Certainty** |
| --- | --- | --- | --- | --- | --- | --- | --- | --- | --- | --- | --- |
| **№ of studies** | **Study design** | **Risk of bias** | **Inconsistency** | **Indirectness** | **Imprecision** | **Other considerations** | **Insulin Detemir 3.9 < FBG ≤ 5.0 mmol/L** | **4.4 < FBG ≤ 6.1 mmol/L** | **Relative**  **(95% CI)** | **Absolute**  **(95% CI)** |
| **HbA1c < 7% Six months, favor 3.9-5.0 mmol/L** | | | | | | | | | | | |
| 1 | randomised trials | not serious^a^ | not serious | not serious | very serious^b^ | none | 78/121 (64.5%) | 66/122 (54.1%) | **RR 1.19**  (0.97 to 1.47) | **103 more per 1,000**  (from 16 fewer to 254 more) | ⨁⨁◯◯  Low |
| **HbA1c endpoint,% Six months, favor 3.9-5.0 mmol/L** | | | | | | | | | | | |
| 1 | randomised trials | not serious^a^ | not serious | not serious | serious^c^ | none | 121 | 122 | - | MD **0.23 lower**  (0.42 lower to 0.04 lower) | ⨁⨁⨁◯  Moderate |
| **Hypoglycemia NR Six months, favor 4.4-6.1 mmol/L** | | | | | | | | | | | |
| 1 | randomised trials | not serious^a^ | not serious | not serious | very serious^b^ | none | 63/121 (52.1%) | 50/122 (41.0%) | **RR 1.27**  (0.97 to 1.67) | **111 more per 1,000**  (from 12 fewer to 275 more) | ⨁⨁◯◯  Low |
| **Nocturnal Hypoglycemia NR Six months, favor 4.4-6.1 mmol/L** | | | | | | | | | | | |
| 1 | randomised trials | not serious^a^ | not serious | not serious | very serious^d^ | none | 37/121 (30.6%) | 25/122 (20.5%) | **RR 1.49**  (0.96 to 2.32) | **100 more per 1,000**  (from 8 fewer to 270 more) | ⨁⨁◯◯  Low |

**CI:** confidence interval; **MD:** mean difference; **RR:** risk ratio.

**Explanations**

a. Downgraded by 0.5 level due to ROB: there are some concerning risks of biases in 2 domains

b. Downgraded by 2 levels due to ROB and imprecision: downgraded by 0.5 due to ROB: there are some concerning risks of biases in 2 domains,downgraded by 1.5 due to imprecision: wide confidence interval and low event rate.

c. Downgraded by 1 level due to ROB and imprecision: downgraded by 0.5 due to ROB: there are some concerning risks of biases in 2 domains,downgraded by 0.5 due to imprecision: small sample size (n = 243).

d. Downgraded by two levels due to imprecision: wide confidence interval and low event rate.

# Appendix Table S4. The clinical thresholds of trial, small, moderate, and large effects for relevant outcomes

**Large effects：**

**HbA1c <7%:** >100 per 1000

**Mean HbA1c change value** (from baseline to the end of the study), %: >1.5

**Hypoglycemia<3.0 mmol/L: >**80 per 1000

**Hypoglycemia<3.9 mmol/L: >**150 per 1000

**Nocturnal hypoglycemia, hypoglycemia<3.0 or 3.9 mmol/L:** >80 per 1000

**Severe hypoglycemia, hypoglycemia<3.0 or 3.9** **mmol/L:** >20 per 1000

**Mean fasting plasma glucose** **(FPG) change value** (from baseline to the end of the study): >1.5 mmol/L

**Mean weight change** (from baseline to the end of the study): >5%

**Moderate effects：**

**HbA1c <7%:** 50-100 per 1000

**Mean HbA1c change value (from baseline to the end of the study), %**: 1.0-1.5

**Hypoglycemia<3.0 mmol/L:** 50-80 per 1000

**Hypoglycemia<3.9 mmol/L:** 100-150 per 1000

**Nocturnal hypoglycemia, hypoglycemia<3.0 or 3.9 mmol/L:** 50-80 per 1000

**Severe hypoglycemia, hypoglycemia<3.0 or 3.9 mmol/L:** 10-20 per 1000

**Mean FPG change value** (from baseline to the end of the study): 1-1.5 mmol/L

**Mean weight change** (from baseline to the end of the study): 3%-5%

**Small effects:**

**HbA1c <7%:** 30-50 per 1000

**Mean HbA1c change value (from baseline to the end of the study), %**: 0.4-1.0

**Hypoglycemia<3.0 mmol/L:** 20**-**50 per 1000

**Hypoglycemia<3.9 mmol/L:** 50-100 per 1000

**Nocturnal hypoglycemia, hypoglycemia<3.0 or 3.9 mmol/L:** 20**-**50 per 1000

**Severe hypoglycemia, hypoglycemia<3.0 or 3.9 mmol/L:** 5-10 per 1000

**Mean FPG change value** (from baseline to the end of the study): 0.5-1.0 mmol/L

**Mean weight change** (from baseline to the end of the study): 2%-3%

**Trivial effects:**

**HbA1c <7%:** <30 per 1000

**Mean HbA1c change value (from baseline to the end of the study), %**: <0.4

**Hypoglycemia<3.0 mmol/L: <**20 per 1000

**Hypoglycemia<3.9 mmol/L: <**50 per 1000

**Nocturnal hypoglycemia, hypoglycemia<3.0 or 3.9 mmol/L: <**20 per 1000

**Severe hypoglycemia, hypoglycemia<3.0 or 3.9 mmol/L:** <5 per 1000

**Mean FPG change value** (from baseline to the end of the study): <0.5 mmol/L

**Mean weight change** (from baseline to the end of the study): <2%

# Appendix Table S5. Patient-reported outcomes

| **Study (Trial name);**  **Country** | **I (N)/ C(N)** | **Trial Duration** | **Patient-reported outcomes^a^** |
| --- | --- | --- | --- |
| **Degludec U-100 Bedtime vs. Glargine U-100 Bedtime** | | | |
| Zinman 2012 (BEGIN Once Long);  Austria, Belgium, Canada, Czech Republic, Denmark, Finland, France, Germany, Norway, Serbia and Montenegro, Spain, and USA | I: 773;  C: 257 | 52 weeks | 773 (100%) / 257 (100%) were assessed at 52 weeks.  A significant group effect on SF-36 questionnaire:  On overall physical: ETD = 1.0, 95% CI 0.1-2.0, P = 0.033, in favor of Degludec U-100 group;  On physical functioning: ETD = 1.4, 95 % CI 0.3 - 2.4, P = 0.016, in favor of Degludec U-100 group.  No significant differences were observed between treatments in other SF-36 domains. |
| Pan 2016;  Brazil, Canada, China, South Africa, Ukraine, and USA | I: 555;  C: 278 | 26 weeks | 555 (100%) / 278 (100%) were assessed at 26 weeks.  A significant group effect on TRIM-D Device:  Degludec U-100 group: 74.3;  Glargine U-100 group: 71.6;  ETD = 2.2, 95 % CI 0.2 - 4.3, in favor of Degludec U-100 group.  No significant group effect results for PRO measured by SF-36 domains. |
| **Glargine U-300 Bedtime vs. Glargine U-100 Bedtime** | | | |
| Bolli 2015, Bolli 2017 (EDITION 3);  North America, Europe and Japan | I: 439;  C: 439 | 24 weeks;  48 weeks | 432 (100%) / 430 (100%) were assessed at 24 weeks.  Not reported whether there were differences between the two groups.  Treatment satisfaction measured by the DTSQs:  Glargine U-300: 31.9±4.7;  Glargine U-100: 31.9±4.8.  Fear of hypoglycaemia assessed on the HFS-II:  Glargine U-300: 0.43±0.48;  Glargine U-100: 0.48±0.52.  There was no change in EQ-5D utility index score from baseline to month 6 in either treatment group.  432 (100%) / 430 (100%) were assessed at 48 weeks.  Not reported whether there were differences between the two groups.  Overall treatment satisfaction measured by the DTSQs:  Glargine U-300: 32.1±4.7;  Glargine U-100: 31.7±5.0.  The total HFS-II score:  Glargine U-300: 0.42±0.47;  Glargine U-100: 0.47±0.53.  EQ-5D utility index score remained stable in both treatment groups throughout the 12 month study. |
| **Glargine U-100 Bedtime vs. NPH Bedtime** | | | |
| Eliaschewitz 2006;  Argentina, Brazil, Chile, Colombia, Guatemala, Mexico,Paraguay, Peru, Uruguay and Venezuela | I: 231;  C: 250 | 24 weeks | 231 (100%) / 250 (100%) were assessed at 24 weeks.  A significant group effect on DTSQc:  Glargine U-100: 12.5±6.3;  NPH: 16.0±3.3, p < 0.02; in favor of Glargine U-100 group. |
| Hsia 2011;  USA | I: 30;  C: 30 | 26 weeks | 30 (100%) / 30 (100%) were assessed at 26 weeks.  No significant group effect results for PRO measured by DTSQ. |
| Hermanns 2015;  Germany | I: 176;  C: 167 | 24 weeks | 118 (67%) / 111 (66.1%) were assessed at 24 weeks.  No significant group effect results for PRO measured by DRQoL, ITEQ, DTSQs, PAID, SF-12® and EQ-5D.  The total score for DRQoL at the end of 24 weeks:  Glargine U-100: 69.7±8.45;  NPH: 69.8±9.81, p-value = 0.97.  The total score for ITEQ at the end of 24 weeks:  Glargine U-100: 74.2±11.8;  NPH: 73.0±13.7, p-value = 0.42.  The total score for DTSQs at the end of 24 weeks:  Glargine U-100: 3.2±8.04;  NPH: 2.1±7.36, p-value = 0.22.  The total score for PAID at the end of 24 weeks:  Glargine U-100: -5.1±11.4;  NPH: -4.4±14.8, p-value = 0.61.  The total score for SF-12® mental health at the end of 24 weeks:  Glargine U-100: 0.5 ± 7.91;  NPH: 0.8 ± 9.21, p-value = 0.77.  The total score for EQ-5D descriptive at the end of 24 weeks:  Glargine U-100: -0.009±0.1727;  NPH: 0.001±0.1606, p-value = 0.62. |
| Home 2015;  Europe (nine sites), Asia (three sites), the Middle East (two sites) and South America (two sites) | I: 352;  C: 349 | 36 weeks | 352 (100%) / 249 (100%) were assessed at 36 weeks.  No significant group effect results for PRO measured by DTSQ.  Glargine U-100: 31.8±4.7;  NPH: 31.0±5.7;  ETD = 0.7, 95 % CI -0.1 - 1.4, p = 0.097. |
| **Glargine U-100 Morning time vs. Glargine U-100 Bedtime** | | | |
| Hsia 2011;  USA | I: 25;  C: 30 | 26 weeks | 25 (100%) / 30 (100%) were assessed at 26 weeks.  No significant group effect results for PRO measured by DTSQ. |
| **Degludec U-100 Morning time vs. Glarging U-100 Morning time** | | | |
| Aso 2017;  Japan | I: 32;  C: 12 | 24 weeks | 32 (100%) / 12 (100%) were assessed at 24 weeks.  A significant group effect on QoL:  Glargine U-100: no changes in any factors;  Degludec U-100: 51.5±26.1, p = 0.0197, in favor of Degludec U-100 group. |
| **Glarging U-100 Morning time vs. NPH Bedtime** | | | |
| Hsia 2011;  USA | I: 25;  C: 30 | 26 weeks | 25 (100%) / 30 (100%) were assessed at 26 weeks.  No significant group effect results for PRO measured by DTSQ. |
| **Glargine U-100 Bedtime vs. Detemir Bedtime** | | | |
| Meneghini 2013  Argentina, India, Republic of Korea, Thailand and USA | I: 227;  C: 226 | 26 weeks | Patient-reported outcomes indicated overall satisfaction with treatment in both insulin arms, with similar improvements in efficacy and productivity scores following initiation of either insulin. Median DiabMedSat scores for efficacy increased from 53 to 72 for both insulins, and scores increased very little for burden (82 to 85) and symptoms (72 to 76) for both insulins.  DPM scores increased for life productivity (75 to 83) and work productivity (85 to 90) for both insulins. |
| C: comparator, CI: confidence interval; DTR-QOL: Diabetes Therapy-Related Quality of life Questionnaire; DRQoL: Diabetes Related Quality of Life; DTSQ: Diabetes Treatment Satisfaction Questionnaire; DTSQc: Diabetes Treatment Satisfaction Questionnaire Change; DTSQs: Diabetes Treatment Satisfaction Questionnaire status version; EQ-5D: the EuroQol 5 Dimensions questionnaire; ETD: estimated treatment difference; HFS-II: the hypoglycaemia fear scale; I: intervention; ITEQ: Insulin Treatment Experience Questionnaire score; NPH: Insulin Protamine Hagedorn; PAID: Problem Areas in Diabetes questionnaire score; PRO: Participant-reported outcomes; SD: standard deviation; SE: standard error; SF-36: Short-Form 36 version 2.0 questionnaire; TRIM-D Device: the Treatment Related Impact Measures for Diabetes and Devices. | | | |

^a^ Data is the mean ± SD.

# Appendix Table S6. The summarized initial dose for the 5 types of basal insulins from 35 included studies

| **Author Year** | **Country** | **Initial dose** |
| --- | --- | --- |
| **Ultra-long-active basal insullin: Glargine U-300** | | |
| Ling 2021 | Hong Kong, China | 0.20 U/kg/day |
| Bolli 2021 | USA, Bulgaria, Croatia, Czechia, Denmark, France, Greece, Hungary, Israel, Italy, Romania, Serbia, Slovakia, Sweden, Switzerland, UK | 0.20 U/kg/day |
| Haluzík 2020 | USA, Bulgaria, Croatia, Czechia, Denmark, France, Greece, Hungary, Israel, Italy, Romania, Serbia, Slovakia, Sweden, Switzerland, UK | 0.20 U/kg/day |
| Ji 2020 | Asia Pacific (including China, South Korea and Taiwan) | 0.20 U/kg/day |
| Cheng 2020 | USA, Bulgaria, Croatia, Czechia, Denmark, France, Greece, Hungary, Israel, Italy, Romania, Serbia, Slovakia, Sweden, Switzerland, UK | 0.20 U/kg/day |
| Rosenstock 2018 | USA, Bulgaria, Croatia, Czechia, Denmark, France, Greece, Hungary, Israel, Italy, Romania, Serbia, Slovakia, Sweden, Switzerland, UK | 0.20 U/kg/day |
| Bolli 2017 | 2 in North America, 12 in Europe, and Japan | 0.20 U/kg/day |
| Bolli 2015 | 15 countries (2 in North America, 12 in Europe, and Japan) | 0.20 U/kg/day |
| **Ultra-long-active basal insullin: Degludec U-100** | | |
| Bolli 2021 | USA, Bulgaria, Croatia, Czechia, Denmark, France, Greece, Hungary, Israel, Italy, Romania, Serbia, Slovakia, Sweden, Switzerland, UK | 0.20 U/kg/day |
| Haluzík 2020 | USA, Bulgaria, Croatia, Czechia, Denmark, France, Greece, Hungary, Israel, Italy, Romania, Serbia, Slovakia, Sweden, Switzerland, UK | 0.20 U/kg/day |
| Cheng 2020 | USA, Bulgaria, Croatia, Czechia, Denmark, France, Greece, Hungary, Israel, Italy, Romania, Serbia, Slovakia, Sweden, Switzerland, UK | 0.20 U/kg/day |
| Rosenstock 2018 | USA, Bulgaria, Croatia, Czechia, Denmark, France, Greece, Hungary, Israel, Italy, Romania, Serbia, Slovakia, Sweden, Switzerland, UK | 0.20 U/kg/day |
| Aso 2017 | Japan | 0.10 U/kg/day |
| Pan 2016 | Brazil, Canada, China, South Africa, Ukraine, and the USA. | 0.14 U/kg/day |
| Onishi 2013 | Hong Kong, Japan, Malaysia, South Korea, Taiwan and Thailand | 0.14U/kg/day |
| Zinman 2012 | Austria, Belgium, Canada, Czech Republic, Denmark, Finland, France, Germany, Norway, Serbia and Montenegro, Spain, and the USA. | 0.12 U/kg/day |
| **Long-active basal insullin: Detemir** | | |
| Elisha 2015 | Canada | 0.12 ± 0.6 U/kg/day |
| Cander 2014 | Turkey | 0.12 U/kg/day |
| Meneghini 2013 | Argentina, India, Republic of Korea, Thailand and USA | 0.12 U/kg/day |
| Blonde 2009 | The USA. | 0.1–0.2 U/kg/day |
| Rosenstock 2008 | 80 sites in Europe and the USA. | 0.14 U/kg/day |
| NCT00506662 | 35 sites in France and 22 sites in UK | 0.20 U/kg/day |
| **Long-active basal insullin: Glargine U-100** | | |
| Yuan 2021 | China | 0.20 U/kg/day |
| Yang 2019 | China | 0.20 U/kg/day |
| Aso 2017 | Japan | 0.10 U/kg/day |
| Pan 2007 | Asian（China,Hong Kong, Indonesia,South Korea, Malaysia, Pakistan,Philippines, Taiwan, Thailand, Singapore） | 0.15 U/kg/day |
| Bolli 2017 | 15 countries (2 in North America, 12 in Europe, and Japan) | 0.20 U/kg/day |
| Pan 2016 | Brazil, Canada, China, South Africa, Ukraine, and USA | 0.14 U/kg/day |
| Hermanns 2015 | Germany | 0.11 U/kg/day |
| Elisha 2015 | Canada | 0.11 ± 0.5 U/kg/day |
| Bolli 2015 | 15 countries (2 in North America, 12 in Europe, and Japan) | 0.20 U/kg/day |
| Home 2015 | Europe (nine), Asia (three), the Middle East (two) and South America (two) | 0.20 U/kg/day |
| Cander 2014 | Turkey | 0.12 U/kg/day |
| Meneghini 2013 | Argentina, India, Republic of Korea, Thailand and USA | 0.12 U/kg/day |
| Onishi 2013 | Hong Kong, Japan, Malaysia, South Korea, Taiwan and Thailand | 0.14U/kg/day |
| Zinman 2012 | Austria, Belgium, Canada, Czech Republic, Denmark, Finland, France, Germany, Norway, Serbia and Montenegro, Spain, and the USA | 0.11 U/kg.day |
| Hsia 2011 | USA | 0.12 U/kg/day to 0.19 U/kg/day |
| Rosenstock 2008 | 80 sites in Europe and the USA | 0.14 U/kg/day |
| Yki-Jarvinen 2006 | Finland and UK | 0.11 U/kg/day to 0.22 U/kg/day |
| **Intermediate-active basal insullin NPH** | | |
| Ling 2021 | Hong Kong, China | 0.20 U/kg/day |
| Hermanns 2015 | Germany | 0.11 U/kg/day |
| Home 2015 | Europe (nine), Asia (three), the Middle East (two) and South America (two) | 0.20 U/kg/day |
| Hsia 2011 | The USA. | 0.12 U/kg/day to 0.19 U/kg/day |
| Pan 2007 | Asian（China,Hong Kong, Indonesia,South Korea, Malaysia, Pakistan,Philippines, Taiwan, Thailand, Singapore） | 0.15 U/kg/day |
| Yki-Jarvinen 2006 | Finland and UK | 0.11 U/kg/day to 0.21 U/kg/day |
| Fritsche 2003 | European countries | 0.23 U/kg/day |
| NCT00506662 | 35 sites in France and 22 sites in the UK | 0.20 U/kg/day |

# Appendix Table S7. The summarized endpoint insulin dose for the 5 types of basal insulins from 35 included studies

| **Author Year** | **Country** | **Duration** | **Mean dose** |
| --- | --- | --- | --- |
| **Ultra-long-active basal insullin: Glargine U-300** | | | |
| Bolli 2021 | Italy | 24 w | 0.48 ± 0.23 U/kg/day to 0.58 ± 0.26 U/kg/day |
| Haluzik 2020 | Czech Republic | 24 w | 0.61 ± 0.018 U/kg/day to 0.47 ± 0.036 U/kg/day |
| Ji 2020 | Asia Pacific (including China, South Korea and Taiwan) | 26 w | 0.34 U/kg/day |
| Cheng 2020 | USA, Bulgaria, Croatia, Czechia, Denmark, France, Greece, Hungary, Israel, Italy, Romania, Serbia, Slovakia, Sweden, Switzerland, UK | 12 w | 0.48 ± 0.21 U/kg/day |
| Rosenstock 2018 | USA, Bulgaria, Croatia, Czechia, Denmark, France, Greece, Hungary, Israel, Italy, Romania, Serbia, Slovakia, Sweden, Switzerland, UK | 24 w | 0.54 ± 0.26 U/kg/day |
| Bolli 2017 | 2 in North America, 12 in Europe, and Japan | 24 w | 0.62 ± 0.29 U/kg/day |
| Bolli 2015 | 15 countries (2 in North America, 12 in Europe, and Japan) | 24 w | 0.62 ± 0.29 U/kg/day |
| **Ultra-long-active basal insullin: Degludec U-100** | | | |
| Bolli 2021 | USA, Bulgaria, Croatia, Czechia, Denmark, France, Greece, Hungary, Israel, Italy, Romania, Serbia, Slovakia, Sweden, Switzerland, UK | 24 w | 0.38 ± 0.20 U/kg/day to 0.45 ± 0.25 U/kg/day |
| Haluzik 2020 | USA, Bulgaria, Croatia, Czechia, Denmark, France, Greece, Hungary, Israel, Italy, Romania, Serbia, Slovakia, Sweden, Switzerland, UK | 24 w | 0.35 ± 0.034 U/kg/day to 0.44 ± 0.016 U/kg/day |
| Cheng 2020 | USA, Bulgaria, Croatia, Czechia, Denmark, France, Greece, Hungary, Israel, Italy, Romania, Serbia, Slovakia, Sweden, Switzerland, UK | 12 w | 0.37 ± 0.20 U/kg/day |
| Rosenstock 2018 | USA, Bulgaria, Croatia, Czechia, Denmark, France, Greece, Hungary, Israel, Italy, Romania, Serbia, Slovakia, Sweden, Switzerland, UK | 24 w | 0.43 ± 0.24 U/kg/day |
| Pan 2016 | Brazil, Canada, China, South Africa, Ukraine, and the USA. | 26 w | 0.49 U/kg/day |
| Onishi 2013 | Hong Kong, Japan, Malaysia, South Korea, Taiwan and Thailand | 26 w | 0.28 U/kg/day |
| Zinman 2012 | Austria, Belgium, Canada, Czech Republic, Denmark, Finland, France, Germany, Norway, Serbia and Montenegro, Spain, and the USA. | 52 w | 0.59 U/kg/day |
| **Long-active basal insullin: Detemir** | | | |
| Elisha 2015 | Canada | 24 w | 0.60 ± 0.50 U/kg/day |
| Cander 2014 | Turkey | 12 w | 0.29 U/kg/day to 0.34 U/kg/day |
| Cander 2014 | Turkey | 12 w | Median: 0.19 U/kg/day to 0.22 U/kg/day |
| Meneghini 2013 | Argentina, India, Republic of Korea, Thailand and USA | 26 w | 0.70 ± 0.34 U/kg/day |
| Blonde 2009 | USA | 20 w | 0.51 U/kg/day to 0.57 U/kg/day |
| Rosenstock 2008 | 80 sites in Europe and the USA | 52 w | 0.78 U/kg/day |
| Tsimikas 2006 | Denmark, France, Italy, The Netherlands, Norway, and Spain and the USA. | 20 w | 0.40 ± 0.20 U/kg/day to 0.50 ± 0.30 U/kg/day |
| **Long-active basal insullin: Glargine U-100** | | | |
| Yuan 2021 | China | 24 w | 0.20 ± 0.1 U/kg/day to 0.30 ± 0.1 U/kg/day |
| Ji 2020 | Mainland China | 16 w | 0.36 U/kg/day to 0.40 U/kg/day |
| Ji 2020 | Asia Pacific(including China, South Korea and Taiwan) | 26 w | 0.29 U/kg/day |
| Yang 2019 | China | 24 w | 0.23 ± 0.11 U/kg/day to 0.28 ± 0.13 U/kg/day |
| Onishi 2013 | Hong Kong, Japan, Malaysia, South Korea, Taiwan and Thailand | 26 w | 0.35 U/kg/day |
| Bolli 2017 | 2 in North America, 12 in Europe, and Japan | 24 w | 0.53 ± 0.24 U/kg/day |
| Pan 2016 | Brazil, Canada, China, South Africa, Ukraine, and the USA. | 26 w | 0.50 U/kg/day |
| Hermanns 2015 | Germany | 24 w | 0.28 ± 0.23 U/kg/day |
| Bolli 2015 | 15 countries (2 in North America, 12 in Europe, and Japan) | 24 w | 0.53 ± 0.24 U/kg/day |
| Home 2015 | Europe (nine), Asia (three), the Middle East (two) and South America (two) | 36 w | 0.39 U/kg/day |
| Elisha 2015 | Canada | 24 w | 0.50 ± 0.30 U/kg/day |
| Cander 2014 | Turkey | 12 w | 0.25 U/kg/day |
| Meneghini 2013 | Argentina, India, Republic of Korea, Thailand and USA | 26 w | 0.61 ± 0.28 U/kg/day |
| Zinman 2012 | Austria, Belgium, Canada, Czech Republic, Denmark, Finland, France, Germany, Norway, Serbia and Montenegro, Spain, and the USA. | 52 w | 0.60 U/kg/day |
| Hsia 2011 | The USA. | 26 w | 0.20 ± 0.11 U/kg/day or 0.21 ± 0.16 U/kg/day |
| Rosenstock 2008 | 80 sites in Europe and the USA. | 52 w | 0.44 U/kg/day |
| Yki-Jarvinen 2006 | Finland and UK | 36 w | 0.69 ± 0.05 U/kg/day |
| Riddle 2003 | 80 sites in the USA. and Canada | 24 w | 0.48 ± 0.01 U/kg/day |
| Fritsche 2003 | European countries | 24 w | 0.48 ± 0.26 U/kg/day to 0.50 ± 0.30 U/kg/day |
| Yki-Jarvinen 2000 | Germany and Finland | 52 w | 0.27 ± 0.01 U/kg/day |
| **Intermediate-active basal insulin: NPH** | | | |
| Home 2015 | Europe (nine), Asia (three), the Middle East (two) and South America (two) | 36 w | 0.36 U/kg/day |
| Hermanns 2015 | Germany | 24 w | 0.27 ± 0.17 U/kg/day |
| Hsia 2011 | The USA. | 26 w | 0.19 ± 0.20 U/kg/day |
| Tsimikas 2006 | Denmark, France, Italy, The Netherlands, Norway, and Spain and the USA. | 20 w | 0.40 ± 0.20 U/kg/day |
| Yki-Jarvinen 2006 | Finland and UK | 36 w | 0.66 ± 0.04 U/kg/day |
| Riddle 2003 | 80 sites in the USA. and Canada | 24 w | 0.42 ± 0.01 U/kg/day |
| Fritsche 2003 | European countries | 24 w | 0.46 ± 0.27 U/kg/day |
| Yki-Jarvinen 2000 | Germany and Finland | 52 w | 0.25 ± 0.01 U/kg/day |
